# Multi-locus analysis of human infective *Cryptosporidium *species and subtypes using ten novel genetic loci

**DOI:** 10.1186/1471-2180-10-213

**Published:** 2010-08-09

**Authors:** Maha Bouzid, Kevin M Tyler, Richard Christen, Rachel M Chalmers, Kristin Elwin, Paul R Hunter

**Affiliations:** 1 Biomedical Research Centre, School of Medicine, Health Policy and Practice, University of East Anglia, Norwich NR4 7TJ, UK; 2 CNRS UMR 6543. Institute of Signalling, Developmental Biology and Cancer Centre de Biochimie, Faculté des Sciences, 06108 Nice cedex 2, France; 3 UK Cryptosporidium Reference Unit, Public Health Wales, Microbiology ABM, Singleton Hospital, Swansea SA2 8QA, UK

## Abstract

**Background:**

*Cryptosporidium *is a protozoan parasite that causes diarrheal illness in a wide range of hosts including humans. Two species, *C. parvum *and *C. hominis *are of primary public health relevance. Genome sequences of these two species are available and show only 3-5% sequence divergence. We investigated this sequence variability, which could correspond either to sequence gaps in the published genome sequences or to the presence of species-specific genes. Comparative genomic tools were used to identify putative species-specific genes and a subset of these genes was tested by PCR in a collection of *Cryptosporidium *clinical isolates and reference strains.

**Results:**

The majority of the putative species-specific genes examined were in fact common to *C. parvum *and *C. hominis*. PCR product sequence analysis revealed interesting SNPs, the majority of which were species-specific. These genetic loci allowed us to construct a robust and multi-locus analysis. The Neighbour-Joining phylogenetic tree constructed clearly discriminated the previously described lineages of *Cryptosporidium *species and subtypes.

**Conclusions:**

Most of the genes identified as being species specific during bioinformatics in *Cryptosporidium *sp. are in fact present in multiple species and only appear species specific because of gaps in published genome sequences. Nevertheless SNPs may offer a promising approach to studying the taxonomy of closely related species of Cryptosporidia.

## Background

At least eight *Cryptosporidium *species infect humans [1]; however, only two species are of major significance to public health by causing the majority of human cases both sporadic and outbreak related cases, *C. hominis *and *C. parvum *[2-5]. *Cryptosporidium parvum *is zoonotic and infects a wide range of animal hosts including humans, whereas *C. hominis *is generally restricted to humans [6]. Therefore, the main phenotypic difference between *C. hominis *and *C. parvum *is the host range [1-3]. In addition, these two *Cryptosporidium *species differ in geographical and temporal distribution and pathogenicity [7,8]. Differential risk factors and transmission routes have also been identified [3,7,9]. However human infections are not solely linked to these two species and other species and genotypes have been associated with illness [10]. These additional species and genotypes are therefore considered emergent. This was the case of the rabbit genotype, the aetiological agent in an outbreak of waterborne human cryptosporidiosis in Northamptonshire, East Midlands, England [11,12]. Subsequent characterization studies revealed that the rabbit genotype, which caused this outbreak, corresponds to *Cryptosporidium cuniculus *(Inman and Takeuchi, 1979) [13].

The public health relevance of *C. parvum *and *C. hominis *has driven a bias in *Cryptosporidium *research towards these two species. Indeed, the genomes of *C. parvum *and *C. hominis *(IOWA and TU502 reference strains, respectively) have been sequenced [14,15]. The genome sequencing of *C. muris*, a less relevant *Cryptosporidium *species from a public health perspective, is underway [16]. The genomic data for all 3 genome representatives is available online http://CryptoDB.org. The genome sizes for *C. parvum *and *C. hominis *are 9.11 and 9.16 Mb, respectively. The GC content is ~ 30% and the coding region is of about 6 Mb [15]. The number of published genes is slightly higher in *C. hominis *than in *C. parvum*: 3,994 genes versus 3,952 genes. The significance of these 42 missing genes is not clear. The average gene length is comparable between the 2 species: 1.57 kb and 1.72 kb, for *C. hominis *and *C. parvum*, respectively. Genome comparison showed that *C. hominis *and *C. parvum *are very similar. This high level of sequence similarity limited the ability of comparative genomics to improve annotation, identify conserved non-coding sequence elements and study gene and protein evolution [16]. More importantly, this high sequence similarity hindered better understanding of host specificity and virulence mechanisms as was anticipated from the genome projects [17]. In fact, *C. hominis *and *C. parvum *genomes exhibit only 3-5% sequence divergence, with no large insertions, deletions or rearrangements [15]. The authors stated that the gene complements of the two species are essentially identical because the few *C. parvum *genes not found in *C. hominis *are proximal to known sequence gaps. However, uncertainty about the amount of sequence variation between *C. parvum *and *C. hominis *persists due to the incomplete status of the *C. hominis *genome. Nevertheless, it has been concluded that the phenotypic differences between *C. hominis *and *C. parvum *are caused by polymorphisms in coding regions and differences in gene regulation [15,18]. The role of this minimal genetic variability between *C. hominis *and *C. parvum *in the phenotypic differences is now much more accessible for investigation. In fact, these genes may include hitherto valuable epidemiological markers and previously unnoticed genetic determinants of host specificity and virulence. In addition, such markers would also serve as typing targets.

The aim of this study was to survey the published *C. parvum *and *C. hominis *genomes for incomplete regions and missing genes in order to identify novel genotyping markers. These genes are likely to contribute to the phenotypic differences between *C. parvum *and *C. hominis *and therefore might be potential genetic determinants of host tropism.

## Results

Initial screening by Reciprocal Blast and retention of coding sequences showing a level of similarity below 10% (and supported by significant p values) identified 117 and 272 putative species-specific genes for *C. hominis *and *C. parvum*, respectively. The majority of *C. parvum *putative specific genes were annotated, while *C. hominis *putative specific genes corresponded mainly to hypothetical proteins. Subsequently, the secondary screen decreased the number of the predicted genes to 93 and 211 genes for *C. hominis *and *C. parvum*, respectively.

**Figure 1:**
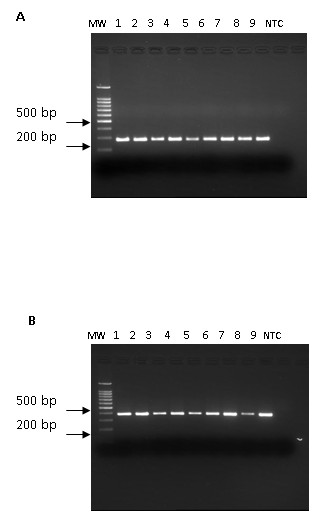
**Amplification of *Cryptosporidium *DNA from clinical isolates and reference strains**. A: amplification of 266 bp of Cgd2_80 gene, B: amplification of 368 bp of Chro.50330 gene. Both *Cryptosporidium *species and all isolates were PCR positive. MW: molecular weight, 1: Cp2, 2: Cp3, 3: Cp4, 4: Ch2, 5:Ch3, 6: Ch4, 7: Iowa, 8: Moredun, 9: TU502, NTC: non template control.

Initially, a subset of ten genes was selected semi-randomly with preference to annotated genes (Table 1). This subset of genes was tested experimentally by PCR in a collection of *Cryptosporidium *clinical isolates and reference strains (Table 2). Surprisingly, 90% (9/10) of the genes tested were present in both *C. hominis and C. parvum*. PCR results for Cgd2_80 and Chro.50330 genes are shown in Figure 1. There was no discernable difference between PCR results of *C. parvum *and *C. hominis *clinical isolates and reference strains by agarose gel electrophoresis. DNA from isolate Cp4 did not amplify using Chro.30149 primers. Further testing of other putative species-specific genes confirmed the general trend. The majority of the predicted genes were therefore common to both *Cryptosporidium *species. Consequently, we considered whether the observed ubiquity of the predicted specific genes represented the closeness between *C. hominis *and *C. parvum *or whether these primers would also amplify orthologous genes from other *Cryptosporidium *species. *C. meleagridis *DNA was amplified by PCR for 8/10 genes (80%), only, *Cgd2_2430 *and *Chro.20156 *PCR reactions were negative (Table 3).

**Table 1:** List of *Cryptosporidium *genes selected for this study.

Primer name	Gene function (CryptoDB)	Sequence	**Tm (°C)**	**Annealing temperature (°C)**	Size of amplified fragment
cgd2_80 F	ABC transporter family protein	GGA TTG GGG GTG ATA TGT TG	68	60	266 bp
cgd2_80 R		ACC TCC AAG CTG TGT TCC AG	70		
cgd6_200 F	Oocyst wall protein 8	CGT TCC AAC AAT GGT GTG TC	68	60	447 bp
cgd6_200 R		GCA GCT GGA GTG CAA TCA TA	68		
cgd8_2370 F	Adenosine kinase like ribokinase	CAG GAA TTG CTC ACG GAA AT	66	60	685 bp
cgd8_2370 R		CCT TAA ATG CAT CCC CAC AG	68		
Chro.50317 F	RNA polymerase A/beta'/A'' subunit	GAT TTT GAT GGA GGG TCT CG	68	60	752 bp
Chro.50317 R		CTG GCA GCT TCA ACA CCA TA	68		
Chro.30149 F	Ubiquitin-protein ligase 1	GGG ATT AGA TGC AGG TGG TG	70	60	331 bp
Chro.30149 R		TGG ATG CTC CAG CAT TAC AT	66		
Chro.50457 F	Erythrocyte membrane-associated antigen	CCT TTG GAT TGT CCC GAA TA	66	60	394 bp
Chro.50457 R		CAA TGC CAT ATG ATT TGA GAA AAA	65		
cgd6_5020 F	Protein with WD40 repeats	AAC AGG AGC TGA CGA TTG CT	60.4	57	271 bp
cgd6_5020 R		ACA TTG TGC CAT TCC AAG GT	58.35		
cgd2_2430 F	Ximpact ortholog conserved protein seen in bacteria and eukaryotes	GTA ACG CAT GGC GAA CCT AT	60.4	57	389 bp
cgd2_2430 R		AAG ATC AGC CTT GCA GCA TT	58.35		
Chro.20156 F	Hypothetical protein	TTC GCT TGA AGC CGT AAA CT	58.35	57	247 bp
Chro.20156 R		GGC ATT GAT ACC AGG CAA GT	60.4		
Chro.50330 F	Leucyl tRNA synthetase	TCG GTA CAG CAT CAG GTT CA	60.4	57	368 bp
Chro.50330 R		GTT TTT GCT CCC CCA GTT TT	58.35		
Cry-15	Oocyst wall protein gene [16]	GTA GAT AAT GGA AGA GAT TGT G	57.08	60	555 bp
Cry-9		GGA CTG AAA TAC AGG CAT TAT CTT G	61.3		

Gene name and annotation is according to CryptoDB. For each gene, a set of primers was designed. Primer name is the gene name followed by F or R (for forward and reverse, respectively). For each gene, primer sequences, annealing temperature and PCR product size are detailed.

**Table 2:** Epidemiological and genotyping data of *Cryptosporidium *isolates tested.

Isolate	Original host	Origin	*COWP*- RFLP	18 s sequencing (genotyping)	*gp60 *sequencing (subtyping)
*C. parvum *IOWA	Bovine (passaged in calves)	Iowa, USA	*C parvum*		
*C. hominis *TU502	Human (passaged in pigs)	Uganda	*C hominis*		
*C. parvum Moredun*	Cervine (passaged in calves)	Scotland	*C parvum*		
Ch2	Human	Yorkshire, England	*C hominis*	*C. hominis ***GQ983348**	IbA10G2 **GQ983356**
Ch3	Human	North Wales	*C hominis*	*C. hominis ***GQ983350**	IbA10G2 **GQ983358**
Ch4	Human	Cumbria, England	*C hominis*	*C. hominis ***GQ983352**	IbA10G2 **GQ983360**
Cp2	Human	Devon, England	*C parvum*	*C parvum ***GQ983349**	IIaA18G3R1 **GQ983357**
Cp3	Human	Cumbria, England	*C parvum*	*C parvum ***GQ983351**	IIaA17G1R1 **GQ983359**
Cp4	Human	Grampian, Scotland	*C parvum*	*C. parvum ***GQ983353**	IIaA15G2R1 **GQ983361**
W7265 (W65)	Human	Leicestershire, England	*C parvum*	*C. parvum ***GU971620**	IIcA5G3 **GU971624**
W7266 (W66)	Human	Leicestershire, England	*C parvum*	*C. parvum ***GU971621**	IIcA5G3 **GU971625**
W7267 (W67)	Human	Leicestershire, England	*C parvum*	*C. parvum ***GU971622**	IIcA5G3 **GU971626**
W7270 (W70)	Human	Leicestershire, England	*C parvum*	*C. parvum ***GU971623**	IIcA5G3 **GU971627**
W17330 (rabbit 1)	Human	Northampton-shire, England	*C hominis*	Rabbit genotype **FJ262726**	VaA18 **FJ262732**
W18455 (rabbit 2)	Human	Shropshire, England	*C hominis*	Rabbit genotype **GU971628**	VaA23 **GU971631**
W17525 (rabbit 3)	Human	Suffolk, England	*C hominis*	Rabbit genotype **GU971629**	VaA32 **GU971632**
(W17435 (rabbit 4)	Human	Essex, England	*C hominis*	Rabbit genotype **GU971630**	VaA22 **GU971633**

Details of the host, the geographical origin and the genotyping data of *C. parvum *and *C. hominis *isolates and reference strains, which DNA was tested during this study.

**Table 3:** PCR results of other *Cryptosporidium *species.

	*C. andersoni*	*C. felis*	Cervine genotype	*C. meleagridis*	*C. baileyi*
**Cgd2_80**	**-**	**-**	**-**	**+**	**-**
**Cgd2_2430**	**+**	**-**	**-**	**-**	**-**
**Cgd6_200**	**-**	**-**	**-**	**+**	**-**
**Cgd6_5020**	**-**	**+**	**-**	**+**	**-**
**Cgd8_2370**	**-**	**-**	**-**	**+**	**-**
**Chro.20156**	**-**	**-**	**-**	**-**	**-**
**Chro.50317**	**-**	**-**	**-**	**+**	**-**
**Chro.50330**	**-**	**-**	**-**	**+**	**-**
**Chro.30149**	**-**	**-**	**+**	**+**	**-**
**Chro.50457**	**-**	**-**	**-**	**+**	**-**

DNA from *C. andersoni*, *C. felis*, cervine genotype, *C. meleagridis *and *C. baileyi *was tested by PCR using the newly designed primers.

Interestingly, for *Cgd2_2430 *gene, only *C. andersoni *DNA was amplified by PCR. For *Cgd6_5020*, only *C. felis *DNA was PCR positive and for *Chro.30149 *primers, cervine genotype DNA was amplified. *C. andersoni*, cervine genotype and *C. felis *DNA was amplified by 10% (1/10) of primers tested. *C. baileyi *DNA was not amplified by any of the primers tested (Table 3).

All positive PCR products were sequenced. PCR product sequences are available online [GenBank: GU904212-GU904405]. The alignments of PCR product sequences for each gene are shown [additional file 1]. One PCR product of *C. meleagridis *DNA using *Chro.50330 *primers did not generate good sequence and was therefore excluded from the analysis. In addition, PCR products for *C. andersoni*, *C. felis *and cervine genotype did not generate good quality sequences and they were not included in the analysis.

Sequence analysis of these novel genetic loci showed interesting genetic polymorphisms and 78 Single nucleotide polymorphisms (SNP) were detected. These SNPs were detected from a total number of 4150 nucleotides, corresponding to an average of 1 SNP every 53 bp. The number of SNPs was variable for each gene, ranging from 1 SNP every 30 bp for *Cgd2_2430 *to less than one SNP per 330 bp for *Chro.30149*. The SNP results for each gene are summarized in Table 4. Of the 78 SNPs, 61 (78.3%) were species-specific, thus defining an interesting feature of this subset of genes identified by comparative genomics. The proportion of species-specific SNPs ranged from 66.7% for *Cgd8_2370 *and *Chro.50317 *genes to 100% for *Chro.50330 *and *Chro.50457 *(Table 4). In addition, 64.2% (50/78) of the SNPs detected were synonymous, thus maintaining the protein sequence. The 28 non-synonymous SNPs were not evenly distributed between the loci. In fact, the proportion of non synonymous SNPs was low for the majority of the genes ranging from 0% to 25% for *Chro.50330 *and *Cgd6_200*, respectively (Table 4). On the contrary, for *Chro.50317 *and *Chro.20156 *genes, 66.7% and 83.4% of the SNPs were non-synonymous. The annotations of these genes are RNA polymerase and hypothetical proteins, respectively. The significance and effect of these mutations would need to be investigated experimentally. In addition to the 61 species-specific SNPs allowing discrimination between *C. hominis *and *C. parvum*, the sequence analysis showed 5 SNPs specific for *C. cuniculus *isolates and 3 SNPs specific for the anthroponotic *C. parvum *subtype. The newly identified SNPs were confirmed experimentally by PCR-RFLP, as sequence alignments were used to identify differential restriction endonuclease recognition sites between the main species tested (Data not shown).

**Table 4:** SNP analysis for the ten loci.

Gene name	Gene annotation	PCR product size	Number of SNPs detected	Average number of nucleotides per SNP	Number of Species specific SNPs (%)	Number of non synonymous SNPs (%)
*Cgd2_80*	ABC transporter family protein	266 bp	7	38	6 (85.5%)	1 (14.3%)
*Cgd2_2430*	Ximpact ortholog conserved protein seen in bacteria and eukaryotes	389 bp	13	30	9 (69.3%)	3 (23.1%)
*Cgd6_200*	Oocyst wall protein 8	447 bp	8	56	6 (75%)	2 (25%)
*Cgd6_5020*	Protein with WD40 repeats	271 bp	2	136	2 (100%)	1 (50%)
*Cgd8_2370*	Adenosine kinase like ribokinase	685 bp	12	58	8 (66.7%)	1 (8.4%)
*Chro.20156*	Hypothetical protein	247 bp	6	42	5 (83.4%)	5 (83.4%)
*Chro.50317*	RNA polymerase A/beta'/A'' subunit	752 bp	15	51	10 (66.7%)	10 (66.7%)
*Chro.50330*	Leucyl tRNA synthetase	368 bp	3	123	3 (100%)	0 (0%)
*Chro.30149*	Ubiquitin-protein ligase 1	331 bp	0	331		
*Chro.50457*	Erythrocyte membrane-associated antigen	394 bp	12	33	12 (100%)	5 (41.7%)

This table details the number of SNPs detected by PCR products sequence analysis of ten novel genetic loci. For each gene, the number and proportion of species-specific SNPs were provided. The effect of the genetic polymorphism on amino acid composition was also indicated.

SNP analysis was performed in a pair-wise manner between isolate groups and subtypes using the logical function "IF" of the Microsoft Excel software to discriminate between variables. When the SNPs were identical between the 2 groups, the value "0" was attributed, while if the 2 SNPs were different, the value "1" was assigned and the values summed for each group. The number of base pair differences between the groups is shown in Table 5. These scores represent the genetic variability between the main isolate groups. The newly identified SNPs showed clear genetic difference patterns between species and subtypes of *Cryptosporidium*. It is noticeable that the genetic differences of *C. hominis *and *C. parvum *to *C. meleagridis *were comparable (5.50 and 5.05%, respectively). This analysis showed a minimal genetic variability between *C. hominis *and *C. parvum *(1.72%) (Table 5). Interestingly, the genetic difference between *C. parvum *and *C. parvum *anthroponotic subtype was 0.13%, while a slightly higher genetic difference was observed between *C. hominis *and *C. cuniculus *isolates (0.27%).

**Table 5:** Genetic differences between *Cryptosporidium *isolates tested.

	*C. hominis*	*C. parvum*	Anthroponotic *C. parvum*	*C. cuniculus*	*C. meleagridis*
*C. hominis*	0				
*C. parvum*	77 (1.72%)	0			
Anthroponotic *C. parvum*	78 (1.75%)	5 (0.12%)	0		
*C. cuniculus*	12 (0.27%)	75 (1.68%)	76 (1.70%)	0	
*C. meleagridis*	157 (5.50%)	144 (5.05%)	144 (5.05%)	155 (5.50%)	0

Based on PCR product sequence analysis, the genetic differences (number and percentage of base pair polymorphisms) between the main strain groups was determined.

**Figure 2:**
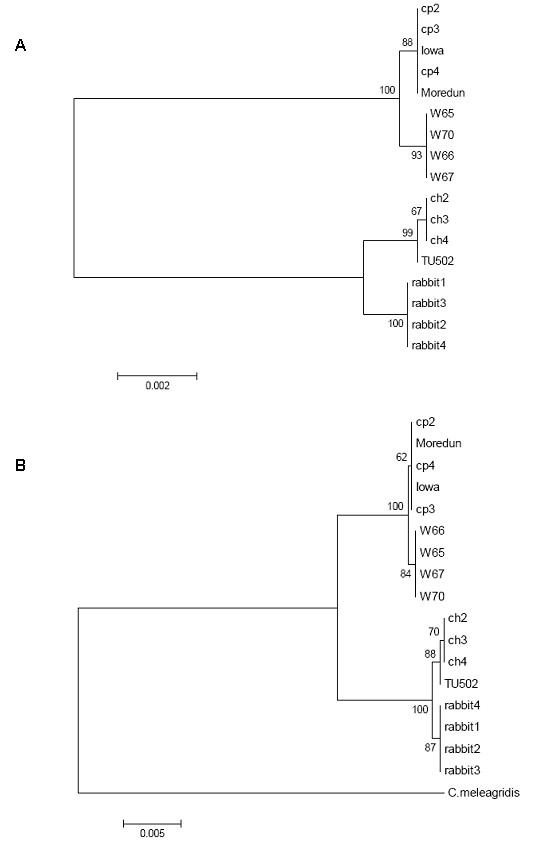
**Phylogenetic Tree based on the gene sequences of 10 new loci and the *COWP *gene sequence**. The trees were constructed using Neighbour-Joining algorithm of MEGA software. A: Phylogenetic tree constructed using *C. parvum*, *C. hominis *and *C. cuniculus *sequences. B: Phylogenetic tree with *C. meleagridis *as an out-group.

Sequences of the ten genetic loci and of the *COWP *(*Cryptosporidium *oocyst wall protein) gene were used for Multi-locus Analysis (MLA). All the retrieved sequences allowed comparison of a total 4469 bp. A Neighbour-Joining Tree was generated based on these sequences using MEGA software. The tree showed clear discrimination between *C. parvum *and *C. hominis *isolates (Figure 2A). Within each group, there were two clusters corresponding to isolate subtypes: *C. parvum *and *C. parvum *anthroponotic subtype and *C. hominis *and *C. cuniculus*. All groups and clusters were supported by high bootstrap values. Unweighted Pair Group Method with Arithmetic Mean (UPGMA) phylogenetic method was also tested to construct phylogenetic trees and gave the same topology with similar bootstrap values (data not shown). There was no discrimination between the different isolates belonging to the main species groups, despite distinct *gp60 *subtypes. However, TU502 strain showed some sequence divergence and was grouped separately within the *C. hominis *cluster. This is due to the presence of a unique SNP at position 132 on Cgd8_2370 gene, which was confirmed by 3 independent rounds of sequencing. *Cryptosporidium meleagridis *sequences were included in the MLA and used as an out group. *Cryptosporidium meleagridis *DNA did amplify 8/10 loci tested, however, for 2 loci (*Cgd8_2370 *and *Chro.50330 *genes) the generated sequences were not of high quality and were not used for analysis. Therefore, the differences between this strain and the other isolates were based only on 2853 bp comparisons for 7 genetic loci. The phylogenetic tree with *C. meleagridis *as the out group also allowed discrimination of *Cryptosporidium *species and subtypes in a similar manner than the tree presented in Figure 2A. The two phylogenetic trees showed similar bootstrap values (Figure 2A and 2B).

## Discussion

In this study, comparative genomic tools were used to identify putative species-specific genes for *C. hominis *and *C. parvum *based on published genome sequences. The initial bioinformatics primary and secondary screening allowed the identification of 93 and 211 genes for *C. hominis *and *C. parvum*, respectively. This finding is somewhat lower than the number of orthologous gene clusters for *C. parvum *and *C. hominis *reported previously in a study of the Apicomplexa [19]. Initially, 10 of these genes were tested by PCR in a collection of *Cryptosporidium *clinical isolates and reference strains. PCR screening of the predicted putative species-specific genes showed that the majority of the genes were not as predicted. In fact, 90% of the genes tested were present in both *C. hominis *and *C. parvum *isolates. This would suggest caution when using lineage-specific genes for taxonomic analysis at least until published genomes are known to be complete [19].

The discrepancy between bioinformatics and PCR is likely to be caused, at least in part, by the fact that the *C. hominis *TU502 genome is neither completed nor fully assembled, which is consistent with the smaller number of putative *C. hominis *specific genes as compared to those specific to *C. parvum*. However, this seems to be in disagreement with the finding that the *C. hominis *genome has 42 genes more than the *C. parvum *genome. Nevertheless, it is plausible that the status of the *C. hominis *genome had hindered the accuracy of the initial comparative genomic analysis because the selected genes may correspond to sequence gaps reported by the authors [15]. Further testing of an additional ten predicted putative specific genes for each species confirmed the general trend of similar amplification from both species. Therefore, the majority of the genes seem to be common to both species. However, an improved comparative genomic analysis has been made possible by the fast progress made towards the completion of *C. muris *genome. At the time of writing, 8.9 Mb from the *C. muris *genome have been made available for download from CryptoDB, of which 7.2 Mb corresponding to coding sequences. Based on these newly added genomic sequences, 7/10 (70%) of the selected putative species-specific genes appear to have orthologs in *C. muris*. This information, if known previously, would have decreased dramatically the number of putative species-specific genes predicted by comparative genomics. Despite this limitation, only one *C. parvum *and one *C. hominis *gene were shown experimentally by PCR to be putatively specific, the characterisation of these genes is ongoing.

We considered whether the observed ubiquity of the predicted specific genes represented the closeness between *C. hominis *and *C. parvum *or whether these primers would also amplify orthologous genes from other *Cryptosporidium *species by testing DNA from *C. andersoni*, *C. felis*, cervine genotype, *C. meleagridis *and *C. baileyi*. *Cryptosporidium meleagridis *DNA amplified using 80% of the primers tested, while, *C. andersoni*, cervine genotype and *C. felis *DNA amplified with only 10% of primers. This result is in accordance with the taxonomy and evolution of *Cryptosporidium *species [20]. In fact, amongst the species tested, *C. meleagridis *is the closest species to the cluster formed by *C. hominis*, *C. parvum *and *C. cuniculus *based on partial *SSU rRNA *gene [20]. *Cryptosporidium meleagridis *DNA did not amplify with primers of Cgd2_2430 and Chro.20156. This could be explained by either nucleotide mismatch in the primer region or that the genes were missing.

PCR screening and sequencing of genes found experimentally to be common to both species provided de novo sequence information at incomplete regions of the *Cryptosporidium *genomes and was used to examine polymorphism in these regions. PCR product sequence analysis revealed interesting genetic variation as SNPs. In this study, 78 SNPs were detected, 78.3% (61) of which were species-specific. The presence of species-specific SNPs was reported previously from several genetic markers and has been exploited for *Cryptosporidium *genotyping and subtyping [21]. PCR-RFLP of the *SSU rRNA *[22], *COWP *[23], *dihydrofolate reductase (DHFR) *gene [24], *thrombospondin related adhesive protein of Cryptosporidium-1 (TRAP-C1) *[25] and *TRAP-C2 *[26], *polythreonine (Poly-T) repeats *[27]*and heat shock protein 70 (HSP70) *[28] genes allow discrimination between *Cryptosporidium *species from various sources. In a similar manner, the newly identified SNPs could be also used for *Cryptosporidium *genotyping, especially by PCR-RFLP and/or sequencing. The majority of the SNPs detected (64.2%) were synonymous. It has long been assumed that synonymous SNPs are inconsequential as the primary sequence of the protein is preserved. However, it has been demonstrated that synonymous mutations can alter the structure, function and expression level of the protein by affecting messenger RNA splicing, stability, protein folding and structure [29]. In addition, Ge and colleagues [30] used a genome wide analysis and described a high number of nucleotide substitution patterns in *C. parvum *and *C. hominis *orthologous protein coding genes. The authors also reported a high number of non-synonymous SNPs in genes involved in host-parasite interactions, mainly genes with transmembrane domains or signal peptides [30].

The sequence analysis of *C. meleagridis *PCR products allowed data enrichment as this species is distant from *C. hominis *and *C. parvum*. In fact, among the genes assessed here, *C. meleagridis *species had 108 additional SNPs, 20 of which are in the *Chro.30149 *gene. For *Chro.30149 *gene, *C. meleagridis *has in average 1 SNP every 15 nucleotide. Surprisingly, all *C. meleagridis *SNPs are synonymous. Interestingly, no SNP was detected in this gene from *C. hominis *and *C. parvum *DNA. *Chro.30149 *has a predicted function as Ubiquitin ligase. This gene is a housekeeping gene and shows a low level of sequence divergence between species and isolates when compared to contingency genes consistently under environmental pressure and characterized by higher spontaneous mutation rates [31].

The newly identified SNPs were used to determine genetic differences between the main *Cryptosporidium *species and subtypes tested. This analysis showed that the genetic difference between *C. hominis *and *C. parvum *was only 1.72%. Within *C. parvum *group, the anthroponotic subtype isolates showed only 0.12% from the main zoonotic *C. parvum *isolates. The *C. cuniculus *isolates exhibited 0.27% genetic differences to *C. hominis *isolates. In addition, extremely low sequence variability between *C. hominis *and *C. cuniculus *was observed using the common genotyping loci [13]. Based on these data and supported by morphological analysis and experimental infection, rabbit genotype was considered synonymous with *C. cuniculus *[13].

In addition, sequence analysis allowed us to perform a robust and novel MLA. The Neighbour-Joining phylogenetic tree clearly grouped and discriminated with high bootstrap values the previously described lineages of *Cryptosporidium *subtypes. Therefore, these genetic loci represent potential powerful targets for *Cryptosporidium *genotyping and subtyping purposes. Especially since these genes are stable and slow mutating, unlike the currently used *Cryptosporidium *typing targets (*gp60*, mini- and microsatellites).

Mini and Microsatellites are repetitive versatile DNA repeats known to influence the structure and expression of protein-coding genes and to be responsive to environmental signals [32,33]. The microsatellites abundance and high variability made them the genetic markers of choice for several applications (individual identity, forensics, parentage, genetic structure, epidemiology and phylogenetics [34]. However, because of the instability of microsatellite markers, extra care should be taken when interpreting microsatellite-based typing data [35]. Similarly, *gp60 *is hypervariable and under selective pressure as it mediates parasite attachment to host cells [36]. In fact, discrepancies and limitations of these markers for *Cryptosporidium *typing have been reported. Hunter and colleagues [37] described the difficulty in interpreting the presence of different subtypes in outbreak setting and Widmer [38] reported that *gp60 *might not be a reliable marker of *C. parvum *and *C. hominis *population structure. The ten novel loci, described in this study, showed excellent discriminatory power and consistency to assess phylogenetic relationships at the species and infra-species levels. These findings suggest that these loci could be alternative valuable genotyping and subtyping targets for *Cryptosporidium*. However, their stability should be assessed in an extensive collection of isolates from different subtype families and geographical locations to validate their discriminatory power.

## Conclusions

In this study, comparative genomics were used to identify putative *C. parvum *and *C. hominis *species-specific genes. Despite the fact that the majority of the predicted genes were common to both species and some to *C. meleagridis*, experimental evidence was found for one specific gene for each species. The ten novel genetic loci studied showed an interesting polymorphism. In fact, sequence analysis of PCR products revealed multiple SNPs, the majority of which were species-specific. These SNPs were stable and consistent across *Cryptosporidium *species and subtypes. These results showed that the ten novel genetic loci can potentially be used to assess the phylogenetic distance and relationships at the species and infra-species level of human infective *Cryptosporidium *isolates. In addition, the paired SNP analysis was found to be a good strategy to assess the genetic divergence of the isolates tested.

## Methods

Reciprocal Blast was used to identify genes with high sequence variability between *C. parvum *and *C. hominis*. This is a variant of Blast (Basic local alignment search tool), originally described by Altschul and colleagues [39] and is a common computational tool for predicting putative orthologs http://www.ncbi.nlm.nih.gov/blast/blast_overview.shtml. Subsequently, each of the ~ 3900 genes of *C. parvum *and *C. hominis *was assigned a similarity score. Only sequences which returned genes with less than 10% sequence similarity from the other genome were considered. These coding sequences are putatively species-specific genes. A secondary screen was performed as follows: each gene was individually tested using Blastn algorithm http://blast.ncbi.nlm.nih.gov/Blast.cgi to confirm specificity and reveal any sequence similarity to genes from other *Cryptosporidium *species. Furthermore, orthology queries were performed using CryptoDB database. Whenever a gene showed sequence similarity, it was eliminated from the selection. This secondary screen increased the prediction stringency.

Amongst the putative species-specific genes, initially 10 genes were selected with preference to annotated genes and tested experimentally by PCR. For each gene, a pair of primers was designed using OligoPerfect™ Designer software http://www.invitrogen.com and supplied by Operon/Eurofins MWG (Cologne, Germany). Table 1 details the genes selected, the primer sequences and the PCR product sizes for each gene tested. In addition, reference primers Cry15 and Cry9 amplifying a 555 bp of the *COWP *gene [23] were used as a positive control. PCR conditions were carried out as described previously [40]. PCR screening of putative species genes was performed by testing a panel of DNA clinical samples isolated as described previously [41] and archived in the national collection at the UK *Cryptosporidium *Reference Unit (CRU) [42]. Each isolate was characterised initially by PCR-RFLP of the *Cryptosporidium *oocyst wall protein (*COWP*) gene [23] and by real-time PCR using simplex Lib 13 primers for *C. parvum *and *C. hominis *[43] prior to sequencing part of the *SSU rRNA *and *gp60 *genes [44,45]. A total number of 14 *Cryptosporidium *clinical isolates was tested (Table 2). This includes DNA from three *C. hominis *isolates (Ch2, Ch3 and Ch4), 3 *C. parvum *isolates (Cp2, Cp3, and Cp4) and 4 *C. parvum *anthroponotic subtype isolates (W7265, W7266, W7267 and W7270). The anthroponotic *C. parvum *group isolates were previously identified as *gp60 *subtype family IIc (CRU unpublished data). This subtype family was reported to infect only humans, and was never reported in an animal species [1]. The anthroponotic nature of the IIc subtype family was supported by extensive subtyping investigations of human and bovine cryptosporidiosis in Portugal, USA, Canada, UK, Ireland, Slovenia, the Netherlands and Australia [1,46-48]. In addition, the DNA of one rabbit genotype (*C. cuniculus*) isolate from the Northamptonshire outbreak [12] and three sporadic cases (Chalmers et al., manuscript in preparation) were also analysed. These DNA samples originated from patients with cryptosporidial diarrhoea from different geographical locations in UK and were chosen as a representative collection of the different strains circulating in the country. Furthermore, the genomic DNA of 3 reference strains *C. parvum *Iowa (ATCC/LGC Promochem, Teddington, UK), *C. parvum *Moredun (Moredun Research Institute, Midlothian, UK) and *C. hominis *TU502 (BEI Resources, Manassas, USA) were tested. Table 5 details the origin and the genotyping data of the tested isolates. In addition, we considered whether the designed primers would amplify orthologous genes from other *Cryptosporidium *species, therefore, DNA from other *Cryptosporidium *species and genotypes was kindly donated by CRU and tested; this includes *C. andersoni *(W13086), *C. felis *(W14508), cervine genotype (W15916), *C. meleagridis *(W10509) and *C. baileyi *(W14184).

Positive PCR products were purified using QIAquick^® ^PCR purification Kit (Qiagen Ltd., Crawley, UK). Purified PCR products were sequenced in both directions using PCR primers. We used 2 independent sequencing facilities: the genome lab, John Innes Centre http://www.jicgenomelab.co.uk and the sequencing service at the University of Dundee http://www.dnaseq.co.uk, both using Dye-terminator chemistry technology and Applied Biosystems automated capillary DNA sequencer (3770 and 3730 model, respectively). Sequences were assembled using CAP3 software http://pbil.univ-lyon1.fr/cap3.php[49] and aligned using AlignX^® ^application of Vector NTI Advance™ 10 software http://www.Invitrogen.com. Phylogenetic analysis was performed using MEGA (Molecular Evolutionary Genetic Analysis) software http://www.megasoftware.net[50].

## Authors' contributions

MB carried out the experimental testing of the predicted putative species-specific genes, sequence alignment and data analysis and drafted the manuscript. KMT conceived the study, provided technical guidance, coordinated the study and helped to draft the manuscript. RC performed the comparative genomic analysis. RMC participated in the design of the study and helped to draft the manuscript. KE carried out DNA extraction from clinical samples and genotyping and subtyping of the isolates at CRU and helped to draft the manuscript. PRH coordinated the study and carried out data analysis and MLA. All authors read and approved the final manuscript.

## Supplementary Material

Additional file 1 **Alignment of PCR product sequences of *Cryptosporidium *clinical isolates and reference strains**. This file shows the PCR product sequences for the ten novel genetic loci and the *COWP *gene. The sequences are available online (see result section). The alignment shows the position of each SNP detected. The totality of the SNPs was used for MLA and calculation of genetic differences between *Cryptosporidium *species and isotypes tested. Click here for file
