# Supplementary material for: Multi-locus analysis of human infective Cryptosporidium species and subtypes using ten novel genetic loci
Source: BMC Microbiol. 2010 Aug 9;10:213. doi: 10.1186/1471-2180-10-213 (PMC2928199; doi:10.1186/1471-2180-10-213)
Supplement: Additional file 1 — Alignment of PCR product sequences of Cryptosporidium clinical isolates and reference strains. This file shows the PCR product sequences for the ten novel genetic loci and the COWP gene. The sequences are available online (see result section). The alignment shows the position of each SNP detected. The totality of the SNPs was used for MLA and calculation of genetic differences between Cryptosporidium species and isotypes tested. [file 1471-2180-10-213-S1.DOC]

**Additional file** **1**

**Cgd2_80 gene PCR products**

1 60

ch2_cgd2_80 (1) TGATATGTTGATTATAGATACATGTATTGTGAAGTCAATTGTTTCCAGTTTAGCACCAGT

ch3_cgd2_80 (1) TGATATGTTGATTATAGATACATGTATTGTGAAGTCAATTGTTTCCAGTTTAGCACCAGT

ch4_cgd2_80 (1) TGATATGTTGATTATAGATACATGTATTGTGAAGTCAATTGTTTCCAGTTTAGCACCAGT

TU502_cgd2_80 (1) TGATATGTTGATTATAGATACATGTATTGTGAAGTCAATTGTTTCCAGTTTAGCACCAGT

rabbit1_cgd2_80 (1) TGATATGTTGATTATAGATACATGTATTGTGAAATCAATTGTTTCCAGTTTAGCACCAGT

rabbit2_cgd2_80 (1) TGATATGTTGATTATAGATACATGTATTGTGAAATCAATTGTTTCCAGTTTAGCACCAGT

rabbit3_cgd2_80 (1) TGATATGTTGATTATAGATACATGTATTGTGAAATCAATTGTTTCCAGTTTAGCACCAGT

rabbit4_cgd2_80 (1) TGATATGTTGATTATAGATACATGTATTGTGAAATCAATTGTTTCCAGTTTAGCACCAGT

cp2_cgd2_80 (1) TGATATGTTGATTATAGATACATGTATTGTGAAGTCAATTATTTCCAGTTTAGCACCAGT

cp3_cgd2_80 (1) TGATATGTTGATTATAGATACATGTATTGTGAAGTCAATTATTTCCAGTTTAGCACCAGT

cp4_cgd2_80 (1) TGATATGTTGATTATAGATACATGTATTGTGAAGTCAATTATTTCCAGTTTAGCACCAGT

Iowa_cgd2_80 (1) TGATATGTTGATTATAGATACATGTATTGTGAAGTCAATTATTTCCAGTTTAGCACCAGT

Moredun_cgd2_80 (1) TGATATGTTGATTATAGATACATGTATTGTGAAGTCAATTATTTCCAGTTTAGCACCAGT

w65_cgd2_80 (1) TGATATGTTGATTATAGATACATGTATTGTGAAGTCAATTATTTCCAGTTTAGCACCAGT

w66_cgd2_80 (1) TGATATGTTGATTATAGATACATGTATTGTGAAGTCAATTATTTCCAGTTTAGCACCAGT

w67_cgd2_80 (1) TGATATGTTGATTATAGATACATGTATTGTGAAGTCAATTATTTCCAGTTTAGCACCAGT

w70_cgd2_80 (1) TGATATGTTGATTATAGATACATGTATTGTGAAGTCAATTATTTCCAGTTTAGCACCAGT

c.meleagridis_cgd2_80 (1) TGATATGTTGATTATAGATACATGTATTGTGAAGTCAATTATTTCCAGTTTAGCACCAGT

61 120

ch2_cgd2_80 (61) ATTGACAATCATAGGTCAGACAATATTTATTGTATATACATTTCCTTATTTTACGCCATT

ch3_cgd2_80 (61) ATTGACAATCATAGGTCAGACAATATTTATTGTATATACATTTCCTTATTTTACGCCATT

ch4_cgd2_80 (61) ATTGACAATCATAGGTCAGACAATATTTATTGTATATACATTTCCTTATTTTACGCCATT

TU502_cgd2_80 (61) ATTGACAATCATAGGTCAGACAATATTTATTGTATATACATTTCCTTATTTTACGCCATT

rabbit1_cgd2_80 (61) ATTGACAATCATAGGTCAGACAATATTTATTGTATATACATTTCCTTATTTTACGCCATT

rabbit2_cgd2_80 (61) ATTGACAATCATAGGTCAGACAATATTTATTGTATATACATTTCCTTATTTTACGCCATT

rabbit3_cgd2_80 (61) ATTGACAATCATAGGTCAGACAATATTTATTGTATATACATTTCCTTATTTTACGCCATT

rabbit4_cgd2_80 (61) ATTGACAATCATAGGTCAGACAATATTTATTGTATATACATTTCCTTATTTTACGCCATT

cp2_cgd2_80 (61) ATTGACAATCATAGGTCAGACAATATTTATTGTATATACATTTCCTTATTTTACACCATT

cp3_cgd2_80 (61) ATTGACAATCATAGGTCAGACAATATTTATTGTATATACATTTCCTTATTTTACACCATT

cp4_cgd2_80 (61) ATTGACAATCATAGGTCAGACAATATTTATTGTATATACATTTCCTTATTTTACACCATT

Iowa_cgd2_80 (61) ATTGACAATCATAGGTCAGACAATATTTATTGTATATACATTTCCTTATTTTACACCATT

Moredun_cgd2_80 (61) ATTGACAATCATAGGTCAGACAATATTTATTGTATATACATTTCCTTATTTTACACCATT

w65_cgd2_80 (61) ATTGACAATCATAGGTCAGACAATATTTATTGTATATACATTTCCTTATTTTACACCATT

w66_cgd2_80 (61) ATTGACAATCATAGGTCAGACAATATTTATTGTATATACATTTCCTTATTTTACACCATT

w67_cgd2_80 (61) ATTGACAATCATAGGTCAGACAATATTTATTGTATATACATTTCCTTATTTTACACCATT

w70_cgd2_80 (61) ATTGACAATCATAGGTCAGACAATATTTATTGTATATACATTTCCTTATTTTACACCATT

c.meleagridis_cgd2_80 (61) ATTGACAATCATAGGTCAGACAATATTTATTGTATATACATTTCCTTATTTTACACCATT

121 180

ch2_cgd2_80 (121) TTTTCTTATATGGATAATGCTTATTATAAAACCAATATGTCTCAAATTTATTTCATCTTA

ch3_cgd2_80 (121) TTTTCTTATATGGATAATGCTTATTATAAAACCAATATGTCTCAAATTTATTTCATCTTA

ch4_cgd2_80 (121) TTTTCTTATATGGATAATGCTTATTATAAAACCAATATGTCTCAAATTTATTTCATCTTA

TU502_cgd2_80 (121) TTTTCTTATATGGATAATGCTTATTATAAAACCAATATGTCTCAAATTTATTTCATCTTA

rabbit1_cgd2_80 (121) TTTTCTTATATGGATAATGCTTATTATAAAACCAATATGTCTCAAATTTATTTCATCTTA

rabbit2_cgd2_80 (121) TTTTCTTATATGGATAATGCTTATTATAAAACCAATATGTCTCAAATTTATTTCATCTTA

rabbit3_cgd2_80 (121) TTTTCTTATATGGATAATGCTTATTATAAAACCAATATGTCTCAAATTTATTTCATCTTA

rabbit4_cgd2_80 (121) TTTTCTTATATGGATAATGCTTATTATAAAACCAATATGTCTCAAATTTATTTCATCTTA

cp2_cgd2_80 (121) TTTTCTTATATGGATAATGCTTATTATAAAGCCGATATGCCTTAAATTTATTTCATCTTA

cp3_cgd2_80 (121) TTTTCTTATATGGATAATGCTTATTATAAAGCCGATATGCCTTAAATTTATTTCATCTTA

cp4_cgd2_80 (121) TTTTCTTATATGGATAATGCTTATTATAAAGCCGATATGCCTTAAATTTATTTCATCTTA

Iowa_cgd2_80 (121) TTTTCTTATATGGATAATGCTTATTATAAAGCCGATATGCCTTAAATTTATTTCATCTTA

Moredun_cgd2_80 (121) TTTTCTTATATGGATAATGCTTATTATAAAGCCGATATGCCTTAAATTTATTTCATCTTA

w65_cgd2_80 (121) TTTTCTTATATGGATAATGCTTATTATAAAGCCGATATGCCTTAAATTTATTTCATCTTA

w66_cgd2_80 (121) TTTTCTTATATGGATAATGCTTATTATAAAGCCGATATGCCTTAAATTTATTTCATCTTA

w67_cgd2_80 (121) TTTTCTTATATGGATAATGCTTATTATAAAGCCGATATGCCTTAAATTTATTTCATCTTA

w70_cgd2_80 (121) TTTTCTTATATGGATAATGCTTATTATAAAGCCGATATGCCTTAAATTTATTTCATCTTA

c.meleagridis_cgd2_80 (121) TTTTCTTATATGGATAATGCTTATTATAAAGCCGATATGCCTTAAATTTATTTCATCTTA

181 240

ch2_cgd2_80 (181) TAGGGAATATCAGAGGTTTTCAATATCTCTTTTTTCCTCAATATGTGGAATATTTTCTGG

ch3_cgd2_80 (181) TAGGGAATATCAGAGGTTTTCAATATCTCTTTTTTCCTCAATATGTGGAATATTTTCTGG

ch4_cgd2_80 (181) TAGGGAATATCAGAGGTTTTCAATATCTCTTTTTTCCTCAATATGTGGAATATTTTCTGG

TU502_cgd2_80 (181) TAGGGAATATCAGAGGTTTTCAATATCTCTTTTTTCCTCAATATGTGGAATATTTTCTGG

rabbit1_cgd2_80 (181) TAGGGAATATCAGAGGTTTTCAATATCTCTTTTTTCCTCAATATGTGGAATATTTTCTGG

rabbit2_cgd2_80 (181) TAGGGAATATCAGAGGTTTTCAATATCTCTTTTTTCCTCAATATGTGGAATATTTTCTGG

rabbit3_cgd2_80 (181) TAGGGAATATCAGAGGTTTTCAATATCTCTTTTTTCCTCAATATGTGGAATATTTTCTGG

rabbit4_cgd2_80 (181) TAGGGAATATCAGAGGTTTTCAATATCTCTTTTTTCCTCAATATGTGGAATATTTTCTGG

cp2_cgd2_80 (181) TAGGGAATATCAGAGGTTTTCAATATCTCTTTTTTCCTCAATATGTGGAATATTTTCTGG

cp3_cgd2_80 (181) TAGGGAATATCAGAGGTTTTCAATATCTCTTTTTTCCTCAATATGTGGAATATTTTCTGG

cp4_cgd2_80 (181) TAGGGAATATCAGAGGTTTTCAATATCTCTTTTTTCCTCAATATGTGGAATATTTTCTGG

Iowa_cgd2_80 (181) TAGGGAATATCAGAGGTTTTCAATATCTCTTTTTTCCTCAATATGTGGAATATTTTCTGG

Moredun_cgd2_80 (181) TAGGGAATATCAGAGGTTTTCAATATCTCTTTTTTCCTCAATATGTGGAATATTTTCTGG

w65_cgd2_80 (181) TAGGGAATATCAGAGGTTTTCAATATCTCTTTTTTCCTCAATATGTGGAATATTTTCTGG

w66_cgd2_80 (181) TAGGGAATATCAGAGGTTTTCAATATCTCTTTTTTCCTCAATATGTGGAATATTTTCTGG

w67_cgd2_80 (181) TAGGGAATATCAGAGGTTTTCAATATCTCTTTTTTCCTCAATATGTGGAATATTTTCTGG

w70_cgd2_80 (181) TAGGGAATATCAGAGGTTTTCAATATCTCTTTTTTCCTCAATATGTGGAATATTTTCTGG

c.meleagridis_cgd2_80 (181) TAGGGAATATCAGAGGTTTGCAATATCTCCTTTTTCCTCAATATGTGGAATATTTTCTGG

241

ch2_cgd2_80 (241) AACACAGC

ch3_cgd2_80 (241) AACACAGC

ch4_cgd2_80 (241) AACACAGC

TU502_cgd2_80 (241) AACACAGC

rabbit1_cgd2_80 (241) AACACAGC

rabbit2_cgd2_80 (241) AACACAGC

rabbit3_cgd2_80 (241) AACACAGC

rabbit4_cgd2_80 (241) AACACAGC

cp2_cgd2_80 (241) AACACAGC

cp3_cgd2_80 (241) AACACAGC

cp4_cgd2_80 (241) AACACAGC

Iowa_cgd2_80 (241) AACACAGC

Moredun_cgd2_80 (241) AACACAGC

w65_cgd2_80 (241) AACACAGC

w66_cgd2_80 (241) AACACAGC

w67_cgd2_80 (241) AACACAGC

w70_cgd2_80 (241) AACACAGC

c.meleagridis_cgd2_80 (241) AACACAGC

**Cgd6_200 gene PCR products**

1 60

ch2_cgd6_200 (1) GGTGTGTCCAACAGGATATACTTTGGATTCAAATAGACAATGCGTTGCTAGAGAGGAGAT

ch3_cgd6_200 (1) GGTGTGTCCAACAGGATATACTTTGGATTCAAATAGACAATGCGTTGCTAGAGAGGAGAT

ch4_cgd6_200 (1) GGTGTGTCCAACAGGATATACTTTGGATTCAAATAGACAATGCGTTGCTAGAGAGGAGAT

TU502_cgd6_200 (1) GGTGTGTCCAACAGGATATACTTTGGATTCAAATAGACAATGCGTTGCTAGAGAGGAGAT

rabbit1_cgd6_200 (1) GGTGTGTCCAACAGGATATACTTTGGATTCAAATAGACAATGCGTTGCTAGAGAGGAGAT

rabbit2_cgd6_200 (1) GGTGTGTCCAACAGGATATACTTTGGATTCAAATAGACAATGCGTTGCTAGAGAGGAGAT

rabbit3_cgd6_200 (1) GGTGTGTCCAACAGGATATACTTTGGATTCAAATAGACAATGCGTTGCTAGAGAGGAGAT

rabbit4_cgd6_200 (1) GGTGTGTCCAACAGGATATACTTTGGATTCAAATAGACAATGCGTTGCTAGAGAGGAGAT

cp2_cgd6_200 (1) GGTGTGTCCAACAGGATATACTTTGGATTCAAATAGACAATGCGTTGCTAGAGAGGAGAT

cp3_cgd6_200 (1) GGTGTGTCCAACAGGATATACTTTGGATTCAAATAGACAATGCGTTGCTAGAGAGGAGAT

cp4_cgd6_200 (1) GGTGTGTCCAACAGGATATACTTTGGATTCAAATAGACAATGCGTTGCTAGAGAGGAGAT

Iowa_cgd6_200 (1) GGTGTGTCCAACAGGATATACTTTGGATTCAAATAGACAATGCGTTGCTAGAGAGGAGAT

Moredun_cgd6_200 (1) GGTGTGTCCAACAGGATATACTTTGGATTCAAATAGACAATGCGTTGCTAGAGAGGAGAT

w65_cgd6_200 (1) GGTGTGTCCAACAGGATATACTTTGGATTCAAATAGACAATGCGTTGCTAGAGAGGAGAT

w66_cgd6_200 (1) GGTGTGTCCAACAGGATATACTTTGGATTCAAATAGACAATGCGTTGCTAGAGAGGAGAT

w67_cgd6_200 (1) GGTGTGTCCAACAGGATATACTTTGGATTCAAATAGACAATGCGTTGCTAGAGAGGAGAT

w70_cgd6_200 (1) GGTGTGTCCAACAGGATATACTTTGGATTCAAATAGACAATGCGTTGCTAGAGAGGAGAT

C. meleagridis_cgd6_200 (1) GGTGTGTCCAACAGGATATACTTTGGATTCAAATAGGCAATGCGTTGCTAGAGAAGAGAT

61 120

ch2_cgd6_200 (61) AATGCCAGAAAGAGTCTGCTTGAATGGAGGAGAATTAATGAGTGATTTGAACTGTATGAA

ch3_cgd6_200 (61) AATGCCAGAAAGAGTCTGCTTGAATGGAGGAGAATTAATGAGTGATTTGAACTGTATGAA

ch4_cgd6_200 (61) AATGCCAGAAAGAGTCTGCTTGAATGGAGGAGAATTAATGAGTGATTTGAACTGTATGAA

TU502_cgd6_200 (61) AATGCCAGAAAGAGTCTGCTTGAATGGAGGAGAATTAATGAGTGATTTGAACTGTATGAA

rabbit1_cgd6_200 (61) AATGCCAGAAAGAGTCTGCTTGAATGGAGGAGAATTAATGAGTGATTTGAACTGTATGAA

rabbit2_cgd6_200 (61) AATGCCAGAAAGAGTCTGCTTGAATGGAGGAGAATTAATGAGTGATTTGAACTGTATGAA

rabbit3_cgd6_200 (61) AATGCCAGAAAGAGTCTGCTTGAATGGAGGAGAATTAATGAGTGATTTGAACTGTATGAA

rabbit4_cgd6_200 (61) AATGCCAGAAAGAGTCTGCTTGAATGGAGGAGAATTAATGAGTGATTTGAACTGTATGAA

cp2_cgd6_200 (61) AATGCCAGAAAGAGTCTGCTTGAATGGAGGAGAATTAATGAGCGATTTTAATTGTATGAA

cp3_cgd6_200 (61) AATGCCAGAAAGAGTCTGCTTGAATGGAGGAGAATTAATGAGCGATTTTAATTGTATGAA

cp4_cgd6_200 (61) AATGCCAGAAAGAGTCTGCTTGAATGGAGGAGAATTAATGAGCGATTTTAATTGTATGAA

Iowa_cgd6_200 (61) AATGCCAGAAAGAGTCTGCTTGAATGGAGGAGAATTAATGAGCGATTTTAATTGTATGAA

Moredun_cgd6_200 (61) AATGCCAGAAAGAGTCTGCTTGAATGGAGGAGAATTAATGAGCGATTTTAATTGTATGAA

w65_cgd6_200 (61) AATGCCAGAAAGAGTCTGCTTGAATGGAGGAGAATTAATGAGTGATTTTAATTGTATGAA

w66_cgd6_200 (61) AATGCCAGAAAGAGTCTGCTTGAATGGAGGAGAATTAATGAGTGATTTTAATTGTATGAA

w67_cgd6_200 (61) AATGCCAGAAAGAGTCTGCTTGAATGGAGGAGAATTAATGAGTGATTTTAATTGTATGAA

w70_cgd6_200 (61) AATGCCAGAAAGAGTCTGCTTGAATGGAGGAGAATTAATGAGTGATTTTAATTGTATGAA

C. meleagridis_cgd6_200 (61) AATGCCAGAAAGAGTCTGCTTGAATGGAGGAGAATTAATGAATGATTTTAATTGTTTGAA

121 180

ch2_cgd6_200 (121) GACAACAAACCCAATCATGAAATGTCCAGTGGAATATACATTGGTAGGTGAAAATATGTG

ch3_cgd6_200 (121) GACAACAAACCCAATCATGAAATGTCCAGTGGAATATACATTGGTAGGTGAAAATATGTG

ch4_cgd6_200 (121) GACAACAAACCCAATCATGAAATGTCCAGTGGAATATACATTGGTAGGTGAAAATATGTG

TU502_cgd6_200 (121) GACAACAAACCCAATCATGAAATGTCCAGTGGAATATACATTGGTAGGTGAAAATATGTG

rabbit1_cgd6_200 (121) GACAACAAACCCAATCATGAAATGTCCAGTGGAATATACATTGGTAGGTGAAAATATGTG

rabbit2_cgd6_200 (121) GACAACAAACCCAATCATGAAATGTCCAGTGGAATATACATTGGTAGGTGAAAATATGTG

rabbit3_cgd6_200 (121) GACAACAAACCCAATCATGAAATGTCCAGTGGAATATACATTGGTAGGTGAAAATATGTG

rabbit4_cgd6_200 (121) GACAACAAACCCAATCATGAAATGTCCAGTGGAATATACATTGGTAGGTGAAAATATGTG

cp2_cgd6_200 (121) GACAACAAACCCAATCATGAAATGTCCAGTGGAATATACATTGGTAGGTGAAAATATGTG

cp3_cgd6_200 (121) GACAACAAACCCAATCATGAAATGTCCAGTGGAATATACATTGGTAGGTGAAAATATGTG

cp4_cgd6_200 (121) GACAACAAACCCAATCATGAAATGTCCAGTGGAATATACATTGGTAGGTGAAAATATGTG

Iowa_cgd6_200 (121) GACAACAAACCCAATCATGAAATGTCCAGTGGAATATACATTGGTAGGTGAAAATATGTG

Moredun_cgd6_200 (121) GACAACAAACCCAATCATGAAATGTCCAGTGGAATATACATTGGTAGGTGAAAATATGTG

w65_cgd6_200 (121) GACAACAAACCCAATCATGAAATGTCCAGTGGAATATACATTGGTAGGTGAAAATATGTG

w66_cgd6_200 (121) GACAACAAACCCAATCATGAAATGTCCAGTGGAATATACATTGGTAGGTGAAAATATGTG

w67_cgd6_200 (121) GACAACAAACCCAATCATGAAATGTCCAGTGGAATATACATTGGTAGGTGAAAATATGTG

w70_cgd6_200 (121) GACAACAAACCCAATCATGAAATGTCCAGTGGAATATACATTGGTAGGTGAAAATATGTG

C. meleagridis_cgd6_200 (121) GACAACAAACCCAATAATGAAATGCCCAGTAGAATATACATTGGTAGGTGAAAATATGTG

181 240

ch2_cgd6_200 (181) TCAAAAGAATGTTGAGATCGATCCAGTTGCTGTATGCCCATCAGGATTTACTTTAACTGA

ch3_cgd6_200 (181) TCAAAAGAATGTTGAGATCGATCCAGTTGCTGTATGCCCATCAGGATTTACTTTAACTGA

ch4_cgd6_200 (181) TCAAAAGAATGTTGAGATCGATCCAGTTGCTGTATGCCCATCAGGATTTACTTTAACTGA

TU502_cgd6_200 (181) TCAAAAGAATGTTGAGATCGATCCAGTTGCTGTATGCCCATCAGGATTTACTTTAACTGA

rabbit1_cgd6_200 (181) TCAAAAGAATGTTGAGATCGATCCAGTTGCTGTATGCCCATCAGGATTTACTTTAACTGA

rabbit2_cgd6_200 (181) TCAAAAGAATGTTGAGATCGATCCAGTTGCTGTATGCCCATCAGGATTTACTTTAACTGA

rabbit3_cgd6_200 (181) TCAAAAGAATGTTGAGATCGATCCAGTTGCTGTATGCCCATCAGGATTTACTTTAACTGA

rabbit4_cgd6_200 (181) TCAAAAGAATGTTGAGATCGATCCAGTTGCTGTATGCCCATCAGGATTTACTTTAACTGA

cp2_cgd6_200 (181) TCAAAAGAATGTTGAGATCGACCCAGTTGCTGTATGCCCATCAGGATTTACTTTAACTGA

cp3_cgd6_200 (181) TCAAAAGAATGTTGAGATCGACCCAGTTGCTGTATGCCCATCAGGATTTACTTTAACTGA

cp4_cgd6_200 (181) TCAAAAGAATGTTGAGATCGACCCAGTTGCTGTATGCCCATCAGGATTTACTTTAACTGA

Iowa_cgd6_200 (181) TCAAAAGAATGTTGAGATCGACCCAGTTGCTGTATGCCCATCAGGATTTACTTTAACTGA

Moredun_cgd6_200 (181) TCAAAAGAATGTTGAGATCGACCCAGTTGCTGTATGCCCATCAGGATTTACTTTAACTGA

w65_cgd6_200 (181) TCAAAAGAATGTTGAGATCGACCCAGTTGCTGTATGCCCATCAGGATTTACTTTAACTGA

w66_cgd6_200 (181) TCAAAAGAATGTTGAGATCGACCCAGTTGCTGTATGCCCATCAGGATTTACTTTAACTGA

w67_cgd6_200 (181) TCAAAAGAATGTTGAGATCGACCCAGTTGCTGTATGCCCATCAGGATTTACTTTAACTGA

w70_cgd6_200 (181) TCAAAAGAATGTTGAGATCGACCCAGTTGCTGTATGCCCATCAGGATTTACTTTAACTGA

C. meleagridis_cgd6_200 (181) TCAAAAGAATGTTGAGATCGATCCAGTTGCTGTTTGCCCATCAGGATTCACTTTAACTGA

241 300

ch2_cgd6_200 (241) CGGTCAAATGTGCAGCGGCTCAAAAACTGTTGCACCCATCAAGAAATGTATGCAAGGAGT

ch3_cgd6_200 (241) CGGTCAAATGTGCAGCGGCTCAAAAACTGTTGCACCCATCAAGAAATGTATGCAAGGAGT

ch4_cgd6_200 (241) CGGTCAAATGTGCAGCGGCTCAAAAACTGTTGCACCCATCAAGAAATGTATGCAAGGAGT

TU502_cgd6_200 (241) CGGTCAAATGTGCAGCGGCTCAAAAACTGTTGCACCCATCAAGAAATGTATGCAAGGAGT

rabbit1_cgd6_200 (241) CGGTCAAATGTGCAGTGGCTCAAAAACTGTTGCACCCATCAAGAAATGTATGCAAGGAGT

rabbit2_cgd6_200 (241) CGGTCAAATGTGCAGTGGCTCAAAAACTGTTGCACCCATCAAGAAATGTATGCAAGGAGT

rabbit3_cgd6_200 (241) CGGTCAAATGTGCAGTGGCTCAAAAACTGTTGCACCCATCAAGAAATGTATGCAAGGAGT

rabbit4_cgd6_200 (241) CGGTCAAATGTGCAGTGGCTCAAAAACTGTTGCACCCATCAAGAAATGTATGCAAGGAGT

cp2_cgd6_200 (241) CGGTCAAATGTGCAGTGGCTCAAAGACTGTTGCACCAATCAAGAAATGTATGCAAGGAGT

cp3_cgd6_200 (241) CGGTCAAATGTGCAGTGGCTCAAAGACTGTTGCACCAATCAAGAAATGTATGCAAGGAGT

cp4_cgd6_200 (241) CGGTCAAATGTGCAGTGGCTCAAAGACTGTTGCACCAATCAAGAAATGTATGCAAGGAGT

Iowa_cgd6_200 (241) CGGTCAAATGTGCAGTGGCTCAAAGACTGTTGCACCAATCAAGAAATGTATGCAAGGAGT

Moredun_cgd6_200 (241) CGGTCAAATGTGCAGTGGCTCAAAGACTGTTGCACCAATCAAGAAATGTATGCAAGGAGT

w65_cgd6_200 (241) CGGTCAAATGTGCAGTGGCTCAAAGACTGTTGCACCAATCAAGAAATGTATGCAAGGAGT

w66_cgd6_200 (241) CGGTCAAATGTGCAGTGGCTCAAAGACTGTTGCACCAATCAAGAAATGTATGCAAGGAGT

w67_cgd6_200 (241) CGGTCAAATGTGCAGTGGCTCAAAGACTGTTGCACCAATCAAGAAATGTATGCAAGGAGT

w70_cgd6_200 (241) CGGTCAAATGTGCAGTGGCTCAAAGACTGTTGCACCAATCAAGAAATGTATGCAAGGAGT

C. meleagridis_cgd6_200 (241) CGGTCAAATGTGCAGTGGTTCAAAGACTGTTGCACCAATCAAGAAATGTATGCAAGGAGT

301 360

ch2_cgd6_200 (301) TTTAAATGAAATGCAAACTGAATGTATCTTACAAAAGAGTGTATCCCCAATTAGTACCTG

ch3_cgd6_200 (301) TTTAAATGAAATGCAAACTGAATGTATCTTACAAAAGAGTGTATCCCCAATTAGTACCTG

ch4_cgd6_200 (301) TTTAAATGAAATGCAAACTGAATGTATCTTACAAAAGAGTGTATCCCCAATTAGTACCTG

TU502_cgd6_200 (301) TTTAAATGAAATGCAAACTGAATGTATCTTACAAAAGAGTGTATCCCCAATTAGTACCTG

rabbit1_cgd6_200 (301) TTTAAATGAAATGCAAACTGAATGTATCTTACAAAAGAGTGTATCCCCAATTAGTACCTG

rabbit2_cgd6_200 (301) TTTAAATGAAATGCAAACTGAATGTATCTTACAAAAGAGTGTATCCCCAATTAGTACCTG

rabbit3_cgd6_200 (301) TTTAAATGAAATGCAAACTGAATGTATCTTACAAAAGAGTGTATCCCCAATTAGTACCTG

rabbit4_cgd6_200 (301) TTTAAATGAAATGCAAACTGAATGTATCTTACAAAAGAGTGTATCCCCAATTAGTACCTG

cp2_cgd6_200 (301) TTTAAATGAAATGCAAAATGAATGTATCTTACAAAAGAGTGTATCCCCAATTAGTACCTG

cp3_cgd6_200 (301) TTTAAATGAAATGCAAAATGAATGTATCTTACAAAAGAGTGTATCCCCAATTAGTACCTG

cp4_cgd6_200 (301) TTTAAATGAAATGCAAAATGAATGTATCTTACAAAAGAGTGTATCCCCAATTAGTACCTG

Iowa_cgd6_200 (301) TTTAAATGAAATGCAAAATGAATGTATCTTACAAAAGAGTGTATCCCCAATTAGTACCTG

Moredun_cgd6_200 (301) TTTAAATGAAATGCAAAATGAATGTATCTTACAAAAGAGTGTATCCCCAATTAGTACCTG

w65_cgd6_200 (301) TTTAAATGAAATGCAAAATGAATGTATCTTACAAAAGAGTGTATCCCCAATTAGTACCTG

w66_cgd6_200 (301) TTTAAATGAAATGCAAAATGAATGTATCTTACAAAAGAGTGTATCCCCAATTAGTACCTG

w67_cgd6_200 (301) TTTAAATGAAATGCAAAATGAATGTATCTTACAAAAGAGTGTATCCCCAATTAGTACCTG

w70_cgd6_200 (301) TTTAAATGAAATGCAAAATGAATGTATCTTACAAAAGAGTGTATCCCCAATTAGTACCTG

C. meleagridis_cgd6_200 (301) CTTAAATGAAATGCAAACAGAATGCATCTTGCAAAAGAGTGTATCCCCAATTAGTACCTG

361 420

ch2_cgd6_200 (361) TCCATCACCTGATTTCACTTTAGTTAGTAATGAAAGATGTGTAAGAGAAGTTTTATATGA

ch3_cgd6_200 (361) TCCATCACCTGATTTCACTTTAGTTAGTAATGAAAGATGTGTAAGAGAAGTTTTATATGA

ch4_cgd6_200 (361) TCCATCACCTGATTTCACTTTAGTTAGTAATGAAAGATGTGTAAGAGAAGTTTTATATGA

TU502_cgd6_200 (361) TCCATCACCTGATTTCACTTTAGTTAGTAATGAAAGATGTGTAAGAGAAGTTTTATATGA

rabbit1_cgd6_200 (361) TCCATCACCTGATTTCACTTTAGTTAGTAATGAAAGATGTGTAAGAGAAGTTTTATATGA

rabbit2_cgd6_200 (361) TCCATCACCTGATTTCACTTTAGTTAGTAATGAAAGATGTGTAAGAGAAGTTTTATATGA

rabbit3_cgd6_200 (361) TCCATCACCTGATTTCACTTTAGTTAGTAATGAAAGATGTGTAAGAGAAGTTTTATATGA

rabbit4_cgd6_200 (361) TCCATCACCTGATTTCACTTTAGTTAGTAATGAAAGATGTGTAAGAGAAGTTTTATATGA

cp2_cgd6_200 (361) TCCATCACCTGATTTCACTTTAGTTAGTAATGAAAGATGTGTAAGAGAAGTTTTATATGA

cp3_cgd6_200 (361) TCCATCACCTGATTTCACTTTAGTTAGTAATGAAAGATGTGTAAGAGAAGTTTTATATGA

cp4_cgd6_200 (361) TCCATCACCTGATTTCACTTTAGTTAGTAATGAAAGATGTGTAAGAGAAGTTTTATATGA

Iowa_cgd6_200 (361) TCCATCACCTGATTTCACTTTAGTTAGTAATGAAAGATGTGTAAGAGAAGTTTTATATGA

Moredun_cgd6_200 (361) TCCATCACCTGATTTCACTTTAGTTAGTAATGAAAGATGTGTAAGAGAAGTTTTATATGA

w65_cgd6_200 (361) TCCATCACCTGATTTCACTTTAGTTAGTAATGAAAGATGTGTAAGAGAAGTTTTATATGA

w66_cgd6_200 (361) TCCATCACCTGATTTCACTTTAGTTAGTAATGAAAGATGTGTAAGAGAAGTTTTATATGA

w67_cgd6_200 (361) TCCATCACCTGATTTCACTTTAGTTAGTAATGAAAGATGTGTAAGAGAAGTTTTATATGA

w70_cgd6_200 (361) TCCATCACCTGATTTCACTTTAGTTAGTAATGAAAGATGTGTAAGAGAAGTTTTATATGA

C. meleagridis_cgd6_200 (361) TCCATCACCTGATTTCACTTTAGTTAGTAATGAAAGATGTGTAAGAGAAGTTTTATATGA

**Cgd8_2370 gene PCR products**

1 60

ch2_cgd8_2370 (1) ACGGAAATTGGAATTGAATTTGAATTCCATATTACTAACAAGGCGAATCTGGAAACAGCA

ch3_cgd8_2370 (1) ACGGAAATTGGAATTGAATTTGAATTCCATATTACTAACAAGGCGAATCTGGAAACAGCA

ch4_cgd8_2370 (1) ACGGAAATTGGAATTGAATTTGAATTCCATATTACTAACAAGGCGAATCTGGAAACAGCA

TU502_cgd8_2370 (1) ACGGAAATTGGAATTGAATTTGAATTCCATATTACTAACAAGGCGAATCTGGAAACAGCA

rabbit1_cgd8_2370 (1) ACGGAAATTGGAATTGAATTTGAATTCCATATTACTAACAAGGCGAATCTGGAAACAGCA

rabbit2_cgd8_2370 (1) ACGGAAATTGGAATTGAATTTGAATTCCATATTACTAACAAGGCGAATCTGGAAACAGCA

rabbit3_cgd8_2370 (1) ACGGAAATTGGAATTGAATTTGAATTCCATATTACTAACAAGGCGAATCTGGAAACAGCA

rabbit4_cgd8_2370 (1) ACGGAAATTGGAATTGAATTTGAATTCCATATTACTAACAAGGCGAATCTGGAAACAGCA

cp2_cgd8_2370 (1) ACGGAAATTGGAATTGAATTTGAATTCCATATTACTAACAAGGCGAATCTGGAAACAGCA

cp3_cgd8_2370 (1) ACGGAAATTGGAATTGAATTTGAATTCCATATTACTAACAAGGCGAATCTGGAAACAGCA

cp4_cgd8_2370 (1) ACGGAAATTGGAATTGAATTTGAATTCCATATTACTAACAAGGCGAATCTGGAAACAGCA

Iowa_cgd8_2370 (1) ACGGAAATTGGAATTGAATTTGAATTCCATATTACTAACAAGGCGAATCTGGAAACAGCA

Moredun_cgd8_2370 (1) ACGGAAATTGGAATTGAATTTGAATTCCATATTACTAACAAGGCGAATCTGGAAACAGCA

w65_cgd8_2370 (1) ACGGAAATTGGAATTGAATTTGAATTCCATATTACTAACAAGGCGAATCTGGAAACAGCA

w66_cgd8_2370 (1) ACGGAAATTGGAATTGAATTTGAATTCCATATTACTAACAAGGCGAATCTGGAAACAGCA

w67_cgd8_2370 (1) ACGGAAATTGGAATTGAATTTGAATTCCATATTACTAACAAGGCGAATCTGGAAACAGCA

w70_cgd8_2370 (1) ACGGAAATTGGAATTGAATTTGAATTCCATATTACTAACAAGGCGAATCTGGAAACAGCA

c.meleagridis_cgd8_2370 (1) ACGGAAATTGGAATTGAATTTGAGTTCCATATTGCTAAAAAGGCGAATCTGGAAACAGCA

61 120

ch2_cgd8_2370 (61) AAATGCGTAGTTTTTGTAACTGAAGAAGAGAGAACGCTTCTAGCAGGATTAGGAGCTGCT

ch3_cgd8_2370 (61) AAATGCGTAGTTTTTGTAACTGAAGAAGAGAGAACGCTTCTAGCAGGATTAGGAGCTGCT

ch4_cgd8_2370 (61) AAATGCGTAGTTTTTGTAACTGAAGAAGAGAGAACGCTTCTAGCAGGATTAGGAGCTGCT

TU502_cgd8_2370 (61) AAATGCGTAGTTTTTGTAACTGAAGAAGAGAGAACGCTTCTAGCAGGATTAGGAGCTGCT

rabbit1_cgd8_2370 (61) AAATGCGTAGTATTTGTAACTGAAGAAGAGAGAACGCTTCTCGCAGGATTAGGAGCTGCT

rabbit2_cgd8_2370 (61) AAATGCGTAGTATTTGTAACTGAAGAAGAGAGAACGCTTCTCGCAGGATTAGGAGCTGCT

rabbit3_cgd8_2370 (61) AAATGCGTAGTATTTGTAACTGAAGAAGAGAGAACGCTTCTCGCAGGATTAGGAGCTGCT

rabbit4_cgd8_2370 (61) AAATGCGTAGTATTTGTAACTGAAGAAGAGAGAACGCTTCTCGCAGGATTAGGAGCTGCT

cp2_cgd8_2370 (61) AAATGCGTAGTTTTTGTAACTGAAGAAGAGAGAACGCTTCTCGCAGGATTAGGAGCTGCT

cp3_cgd8_2370 (61) AAATGCGTAGTTTTTGTAACTGAAGAAGAGAGAACGCTTCTCGCAGGATTAGGAGCTGCT

cp4_cgd8_2370 (61) AAATGCGTAGTTTTTGTAACTGAAGAAGAGAGAACGCTTCTCGCAGGATTAGGAGCTGCT

Iowa_cgd8_2370 (61) AAATGCGTAGTTTTTGTAACTGAAGAAGAGAGAACGCTTCTCGCAGGATTAGGAGCTGCT

Moredun_cgd8_2370 (61) AAATGCGTAGTTTTTGTAACTGAAGAAGAGAGAACGCTTCTCGCAGGATTAGGAGCTGCT

w65_cgd8_2370 (61) AAATGCGTAGTTTTTGTAACTGAAGAAGAGAGAACGCTTCTCGCAGGATTAGGAGCTGCT

w66_cgd8_2370 (61) AAATGCGTAGTTTTTGTAACTGAAGAAGAGAGAACGCTTCTCGCAGGATTAGGAGCTGCT

w67_cgd8_2370 (61) AAATGCGTAGTTTTTGTAACTGAAGAAGAGAGAACGCTTCTCGCAGGATTAGGAGCTGCT

w70_cgd8_2370 (61) AAATGCGTAGTTTTTGTAACTGAAGAAGAGAGAACGCTTCTCGCAGGATTAGGAGCTGCT

c.meleagridis_cgd8_2370 (61) AAATGCGTAGTTTTTGTAACTGAAGAAGAGAGAACACTTCTCGCGGGATTAGGAGCTGCT

121 180

ch2_cgd8_2370 (121) AAGGAATATTCAATTACCACTTTTGAGTCAGAAAATATTCAACATGCTTTAAAGACCGCA

ch3_cgd8_2370 (121) AAGGAATATTCAATTACCACTTTTGAGTCAGAAAATATTCAACATGCTTTAAAGACCGCA

ch4_cgd8_2370 (121) AAGGAATATTCAATTACCACTTTTGAGTCAGAAAATATTCAACATGCTTTAAAGACCGCA

TU502_cgd8_2370 (121) AAGGAATATTCGATTACCACTTTTGAGTCAGAAAATATTCAACATGCTTTAAAGACCGCA

rabbit1_cgd8_2370 (121) AAGGAATATTCGATTACCACTTTTGAGTCAGAAAATATTCAACATGCTTTAAAGACCGCA

rabbit2_cgd8_2370 (121) AAGGAATATTCGATTACCACTTTTGAGTCAGAAAATATTCAACATGCTTTAAAGACCGCA

rabbit3_cgd8_2370 (121) AAGGAATATTCGATTACCACTTTTGAGTCAGAAAATATTCAACATGCTTTAAAGACCGCA

rabbit4_cgd8_2370 (121) AAGGAATATTCGATTACCACTTTTGAGTCAGAAAATATTCAACATGCTTTAAAGACCGCA

cp2_cgd8_2370 (121) AAGGAATATTCGATTACCACTTTTGAGTCAGAAAATATTCAACATGCTTTAAAGACCGCA

cp3_cgd8_2370 (121) AAGGAATATTCGATTACCACTTTTGAGTCAGAAAATATTCAACATGCTTTAAAGACCGCA

cp4_cgd8_2370 (121) AAGGAATATTCGATTACCACTTTTGAGTCAGAAAATATTCAACATGCTTTAAAGACCGCA

Iowa_cgd8_2370 (121) AAGGAATATTCGATTACCACTTTTGAGTCAGAAAATATTCAACATGCTTTAAAGACCGCA

Moredun_cgd8_2370 (121) AAGGAATATTCGATTACCACTTTTGAGTCAGAAAATATTCAACATGCTTTAAAGACCGCA

w65_cgd8_2370 (121) AAGGAATATTCGATTACCACTTTTGAGTCAGAAAATATTCAACATGCTTTAAAGACCGCA

w66_cgd8_2370 (121) AAGGAATATTCGATTACCACTTTTGAGTCAGAAAATATTCAACATGCTTTAAAGACCGCA

w67_cgd8_2370 (121) AAGGAATATTCGATTACCACTTTTGAGTCAGAAAATATTCAACATGCTTTAAAGACCGCA

w70_cgd8_2370 (121) AAGGAATATTCGATTACCACTTTTGAGTCAGAAAATATTCAACATGCTTTAAAGACCGCA

c.meleagridis_cgd8_2370 (121) AAGGAATATTCTATTTCCACTTTTGAGTCAGAAAAGATTCAAAATGCTTTAAAGACCGCA

181 240

ch2_cgd8_2370 (181) AATATATTCGCAACAAGTGGATTCTTTGTTGAAGTTTGTTTCCAGGCAATTCTTAAATCT

ch3_cgd8_2370 (181) AATATATTCGCAACAAGTGGATTCTTTGTTGAAGTTTGTTTCCAGGCAATTCTTAAATCT

ch4_cgd8_2370 (181) AATATATTCGCAACAAGTGGATTCTTTGTTGAAGTTTGTTTCCAGGCAATTCTTAAATCT

TU502_cgd8_2370 (181) AATATATTCGCAACAAGTGGATTCTTTGTTGAAGTTTGTTTCCAGGCAATTCTTAAATCT

rabbit1_cgd8_2370 (181) AATATATTCGCAACAAGTGGATTCTTTGTTGAAGTTTGTTTCCAGGCAATTCTTAAATCT

rabbit2_cgd8_2370 (181) AATATATTCGCAACAAGTGGATTCTTTGTTGAAGTTTGTTTCCAGGCAATTCTTAAATCT

rabbit3_cgd8_2370 (181) AATATATTCGCAACAAGTGGATTCTTTGTTGAAGTTTGTTTCCAGGCAATTCTTAAATCT

rabbit4_cgd8_2370 (181) AATATATTCGCAACAAGTGGATTCTTTGTTGAAGTTTGTTTCCAGGCAATTCTTAAATCT

cp2_cgd8_2370 (181) AATATATTCGCAACAAGTGGATTCTTTGTTGAAGTTTGTTTCCAGGCAATTCTTAAATCT

cp3_cgd8_2370 (181) AATATATTCGCAACAAGTGGATTCTTTGTTGAAGTTTGTTTCCAGGCAATTCTTAAATCT

cp4_cgd8_2370 (181) AATATATTCGCAACAAGTGGATTCTTTGTTGAAGTTTGTTTCCAGGCAATTCTTAAATCT

Iowa_cgd8_2370 (181) AATATATTCGCAACAAGTGGATTCTTTGTTGAAGTTTGTTTCCAGGCAATTCTTAAATCT

Moredun_cgd8_2370 (181) AATATATTCGCAACAAGTGGATTCTTTGTTGAAGTTTGTTTCCAGGCAATTCTTAAATCT

w65_cgd8_2370 (181) AATATATTCGCAACAAGTGGATTCTTTGTTGAAGTTTGTTTCCAGGCAATTCTTAAATCT

w66_cgd8_2370 (181) AATATATTCGCAACAAGTGGATTCTTTGTTGAAGTTTGTTTCCAGGCAATTCTTAAATCT

w67_cgd8_2370 (181) AATATATTCGCAACAAGTGGATTCTTTGTTGAAGTTTGTTTCCAGGCAATTCTTAAATCT

w70_cgd8_2370 (181) AATATATTCGCAACAAGTGGATTCTTTGTTGAAGTTTGTTTCCAGGCAATTCTTAAATCT

c.meleagridis_cgd8_2370 (181) AGTATATTCGCAACAAGTGGATTCTTTGTTGAAGTTTGTTTCCAGGCAATTCTTAAATCT

241 300

ch2_cgd8_2370 (241) GCTCAATATATTCATCAATTTAGATCTAACGAATGTTCTTTTGTATTTGGACTTTCAGCT

ch3_cgd8_2370 (241) GCTCAATATATTCATCAATTTAGATCTAACGAATGTTCTTTTGTATTTGGACTTTCAGCT

ch4_cgd8_2370 (241) GCTCAATATATTCATCAATTTAGATCTAACGAATGTTCTTTTGTATTTGGACTTTCAGCT

TU502_cgd8_2370 (241) GCTCAATATATTCATCAATTTAGATCTAACGAATGTTCTTTTGTATTTGGACTTTCAGCT

rabbit1_cgd8_2370 (241) GCTCAATATATTCATCAATTTAGATCTAACGAATGTTCTTTTGTATTTGGACTTTCAGCT

rabbit2_cgd8_2370 (241) GCTCAATATATTCATCAATTTAGATCTAACGAATGTTCTTTTGTATTTGGACTTTCAGCT

rabbit3_cgd8_2370 (241) GCTCAATATATTCATCAATTTAGATCTAACGAATGTTCTTTTGTATTTGGACTTTCAGCT

rabbit4_cgd8_2370 (241) GCTCAATATATTCATCAATTTAGATCTAACGAATGTTCTTTTGTATTTGGACTTTCAGCT

cp2_cgd8_2370 (241) GCTCAATATATTCATCAATTTAGATCTAACGAATGTTCATTTGTATTTGGACTTTCAGCT

cp3_cgd8_2370 (241) GCTCAATATATTCATCAATTTAGATCTAACGAATGTTCATTTGTATTTGGACTTTCAGCT

cp4_cgd8_2370 (241) GCTCAATATATTCATCAATTTAGATCTAACGAATGTTCATTTGTATTTGGACTTTCAGCT

Iowa_cgd8_2370 (241) GCTCAATATATTCATCAATTTAGATCTAACGAATGTTCATTTGTATTTGGACTTTCAGCT

Moredun_cgd8_2370 (241) GCTCAATATATTCATCAATTTAGATCTAACGAATGTTCATTTGTATTTGGACTTTCAGCT

w65_cgd8_2370 (241) GCTCAATATATTCATCAATTTAGATCTAACGAATGTTCATTTGTATTTGGGCTTTCAGCT

w66_cgd8_2370 (241) GCTCAATATATTCATCAATTTAGATCTAACGAATGTTCATTTGTATTTGGGCTTTCAGCT

w67_cgd8_2370 (241) GCTCAATATATTCATCAATTTAGATCTAACGAATGTTCATTTGTATTTGGGCTTTCAGCT

w70_cgd8_2370 (241) GCTCAATATATTCATCAATTTAGATCTAACGAATGTTCATTTGTATTTGGGCTTTCAGCT

c.meleagridis_cgd8_2370 (241) GCTCAATATATTCATCAATTCAGGTCTGACGAATGTTCCTTTGTGTTCGGACTTTCAGCT

301 360

ch2_cgd8_2370 (301) ACTTATATTCCGGAAAAGTATATGAATGAGTTATTCCAACTTCTACCAATGATTGATTAT

ch3_cgd8_2370 (301) ACTTATATTCCGGAAAAGTATATGAATGAGTTATTCCAACTTCTACCAATGATTGATTAT

ch4_cgd8_2370 (301) ACTTATATTCCGGAAAAGTATATGAATGAGTTATTCCAACTTCTACCAATGATTGATTAT

TU502_cgd8_2370 (301) ACTTATATTCCGGAAAAGTATATGAATGAGTTATTCCAACTTCTACCAATGATTGATTAT

rabbit1_cgd8_2370 (301) ACTTATATTCCGGAAAAGTATATGAATGAGTTATTCCAACTTCTACCAATGATTGATTAT

rabbit2_cgd8_2370 (301) ACTTATATTCCGGAAAAGTATATGAATGAGTTATTCCAACTTCTACCAATGATTGATTAT

rabbit3_cgd8_2370 (301) ACTTATATTCCGGAAAAGTATATGAATGAGTTATTCCAACTTCTACCAATGATTGATTAT

rabbit4_cgd8_2370 (301) ACTTATATTCCGGAAAAGTATATGAATGAGTTATTCCAACTTCTACCAATGATTGATTAT

cp2_cgd8_2370 (301) ACTTATATTCCAGAAAAGTATATGAATGAGTTATTCCAACTTCTACCAATGATTGATTAT

cp3_cgd8_2370 (301) ACTTATATTCCAGAAAAGTATATGAATGAGTTATTCCAACTTCTACCAATGATTGATTAT

cp4_cgd8_2370 (301) ACTTATATTCCAGAAAAGTATATGAATGAGTTATTCCAACTTCTACCAATGATTGATTAT

Iowa_cgd8_2370 (301) ACTTATATTCCAGAAAAGTATATGAATGAGTTATTCCAACTTCTACCAATGATTGATTAT

Moredun_cgd8_2370 (301) ACTTATATTCCAGAAAAGTATATGAATGAGTTATTCCAACTTCTACCAATGATTGATTAT

w65_cgd8_2370 (301) ACTTATATTCCAGAAAAGTATATGAATGAGTTATTCCAACTTCTACCAATGATTGATTAT

w66_cgd8_2370 (301) ACTTATATTCCAGAAAAGTATATGAATGAGTTATTCCAACTTCTACCAATGATTGATTAT

w67_cgd8_2370 (301) ACTTATATTCCAGAAAAGTATATGAATGAGTTATTCCAACTTCTACCAATGATTGATTAT

w70_cgd8_2370 (301) ACTTATATTCCAGAAAAGTATATGAATGAGTTATTCCAACTTCTACCAATGATTGATTAT

c.meleagridis_cgd8_2370 (301) ACTTATATTCCAGAAAAGTATATAAATGAGTTAATTCAACTTTTTCCAATGATTGACTAT

361 420

ch2_cgd8_2370 (361) ATCATTGGAAACCAAGAAGAATTTGTCTCTTTATATAAAAGTATCAACAATATTCTTCAA

ch3_cgd8_2370 (361) ATCATTGGAAACCAAGAAGAATTTGTCTCTTTATATAAAAGTATCAACAATATTCTTCAA

ch4_cgd8_2370 (361) ATCATTGGAAACCAAGAAGAATTTGTCTCTTTATATAAAAGTATCAACAATATTCTTCAA

TU502_cgd8_2370 (361) ATCATTGGAAACCAAGAAGAATTTGTCTCTTTATATAAAAGTATCAACAATATTCTTCAA

rabbit1_cgd8_2370 (361) ATCATTGGAAACCAAGAAGAATTTGTCTCTTTATATAAAAGTATCAACAATATTCTTCAA

rabbit2_cgd8_2370 (361) ATCATTGGAAACCAAGAAGAATTTGTCTCTTTATATAAAAGTATCAACAATATTCTTCAA

rabbit3_cgd8_2370 (361) ATCATTGGAAACCAAGAAGAATTTGTCTCTTTATATAAAAGTATCAACAATATTCTTCAA

rabbit4_cgd8_2370 (361) ATCATTGGAAACCAAGAAGAATTTGTCTCTTTATATAAAAGTATCAACAATATTCTTCAA

cp2_cgd8_2370 (361) ATCATTGGAAACCAAGAAGAATTTGTCTCTTTATATAAAAGTATCAACAATATCCTTCAA

cp3_cgd8_2370 (361) ATCATTGGAAACCAAGAAGAATTTGTCTCTTTATATAAAAGTATCAACAATATCCTTCAA

cp4_cgd8_2370 (361) ATCATTGGAAACCAAGAAGAATTTGTCTCTTTATATAAAAGTATCAACAATATCCTTCAA

Iowa_cgd8_2370 (361) ATCATTGGAAACCAAGAAGAATTTGTCTCTTTATATAAAAGTATCAACAATATCCTTCAA

Moredun_cgd8_2370 (361) ATCATTGGAAACCAAGAAGAATTTGTCTCTTTATATAAAAGTATCAACAATATCCTTCAA

w65_cgd8_2370 (361) ATCATTGGAAACCAAGAAGAATTTGTCTCTTTATATAAAAGTATCAACAATATCCTTCAA

w66_cgd8_2370 (361) ATCATTGGAAACCAAGAAGAATTTGTCTCTTTATATAAAAGTATCAACAATATCCTTCAA

w67_cgd8_2370 (361) ATCATTGGAAACCAAGAAGAATTTGTCTCTTTATATAAAAGTATCAACAATATCCTTCAA

w70_cgd8_2370 (361) ATCATTGGAAACCAAGAAGAATTTGTCTCTTTATATAAAAGTATCAACAATATCCTTCAA

c.meleagridis_cgd8_2370 (361) ATCATTGGAAACCAAGAAGAATTTGTATCTCTATTTAAAAGTATCAACAGTATTTTTCAA

421 480

ch2_cgd8_2370 (421) ATTGAAGACGATGACCAACTATTACTTTCACAGGATAATATAAATCAACCAGAAAACGAT

ch3_cgd8_2370 (421) ATTGAAGACGATGACCAACTATTACTTTCACAGGATAATATAAATCAACCAGAAAACGAT

ch4_cgd8_2370 (421) ATTGAAGACGATGACCAACTATTACTTTCACAGGATAATATAAATCAACCAGAAAACGAT

TU502_cgd8_2370 (421) ATTGAAGACGATGACCAACTATTACTTTCACAGGATAATATAAATCAACCAGAAAACGAT

rabbit1_cgd8_2370 (421) ATTGAAGACGATGACCAACTATTACTTTCACAGGATAATATAAATCAACCAGAAAACGAT

rabbit2_cgd8_2370 (421) ATTGAAGACGATGACCAACTATTACTTTCACAGGATAATATAAATCAACCAGAAAACGAT

rabbit3_cgd8_2370 (421) ATTGAAGACGATGACCAACTATTACTTTCACAGGATAATATAAATCAACCAGAAAACGAT

rabbit4_cgd8_2370 (421) ATTGAAGACGATGACCAACTATTACTTTCACAGGATAATATAAATCAACCAGAAAACGAT

cp2_cgd8_2370 (421) ATTGAAGACGATGACCAACTATTACTTTCGCAGGATAATATAAATCAACCAGAAAACGAT

cp3_cgd8_2370 (421) ATTGAAGACGATGACCAACTATTACTTTCGCAGGATAATATAAATCAACCAGAAAACGAT

cp4_cgd8_2370 (421) ATTGAAGACGATGACCAACTATTACTTTCGCAGGATAATATAAATCAACCAGAAAACGAT

Iowa_cgd8_2370 (421) ATTGAAGACGATGACCAACTATTACTTTCGCAGGATAATATAAATCAACCAGAAAACGAT

Moredun_cgd8_2370 (421) ATTGAAGACGATGACCAACTATTACTTTCGCAGGATAATATAAATCAACCAGAAAACGAT

w65_cgd8_2370 (421) ATTGAAGACGATGACCAACTATTACTTTCGCAGGATAATATAAATCAACCAGAAAACGAT

w66_cgd8_2370 (421) ATTGAAGACGATGACCAACTATTACTTTCGCAGGATAATATAAATCAACCAGAAAACGAT

w67_cgd8_2370 (421) ATTGAAGACGATGACCAACTATTACTTTCGCAGGATAATATAAATCAACCAGAAAACGAT

w70_cgd8_2370 (421) ATTGAAGACGATGACCAACTATTACTTTCGCAGGATAATATAAATCAACCAGAAAACGAT

c.meleagridis_cgd8_2370 (421) ATTAAAGACGATGATCAACTATTACTTTCACAAGATAACATTGGTCAGCCAGAAAATGAT

481 540

ch2_cgd8_2370 (481) GCTTTAGAAAGAATTCTCACAGAAATTCATAAACATCTTAAACCCACATGTATTATACTA

ch3_cgd8_2370 (481) GCTTTAGAAAGAATTCTCACAGAAATTCATAAACATCTTAAACCCACATGTATTATACTA

ch4_cgd8_2370 (481) GCTTTAGAAAGAATTCTCACAGAAATTCATAAACATCTTAAACCCACATGTATTATACTA

TU502_cgd8_2370 (481) GCTTTAGAAAGAATTCTCACAGAAATTCATAAACATCTTAAACCCACATGTATTATACTA

rabbit1_cgd8_2370 (481) GCTTTAGAAAGAATTCTCACAGAAATTCATAAACATCTTAAACCCACATGTATTATACTA

rabbit2_cgd8_2370 (481) GCTTTAGAAAGAATTCTCACAGAAATTCATAAACATCTTAAACCCACATGTATTATACTA

rabbit3_cgd8_2370 (481) GCTTTAGAAAGAATTCTCACAGAAATTCATAAACATCTTAAACCCACATGTATTATACTA

rabbit4_cgd8_2370 (481) GCTTTAGAAAGAATTCTCACAGAAATTCATAAACATCTTAAACCCACATGTATTATACTA

cp2_cgd8_2370 (481) GCTTTAGAAAGAATTCTCACAGAAATTCATAAGCATCTTAAACCCACATGTATTATACTA

cp3_cgd8_2370 (481) GCTTTAGAAAGAATTCTCACAGAAATTCATAAGCATCTTAAACCCACATGTATTATACTA

cp4_cgd8_2370 (481) GCTTTAGAAAGAATTCTCACAGAAATTCATAAGCATCTTAAACCCACATGTATTATACTA

Iowa_cgd8_2370 (481) GCTTTAGAAAGAATTCTCACAGAAATTCATAAGCATCTTAAACCCACATGTATTATACTA

Moredun_cgd8_2370 (481) GCTTTAGAAAGAATTCTCACAGAAATTCATAAGCATCTTAAACCCACATGTATTATACTA

w65_cgd8_2370 (481) GCTTTAGAAAGAATTCTCACAGAAATTCATAAGCATCTTAAACCCACATGTATTATACTA

w66_cgd8_2370 (481) GCTTTAGAAAGAATTCTCACAGAAATTCATAAGCATCTTAAACCCACATGTATTATACTA

w67_cgd8_2370 (481) GCTTTAGAAAGAATTCTCACAGAAATTCATAAGCATCTTAAACCCACATGTATTATACTA

w70_cgd8_2370 (481) GCTTTAGAAAGAATTCTCACAGAAATTCATAAGCATCTTAAACCCACATGTATTATACTA

c.meleagridis_cgd8_2370 (481) GTTTTAGAAATCATTCTCACAGAAATTCATAAACATCTTAAGCCCACATGTATTATGCTA

541 600

ch2_cgd8_2370 (541) TGTACAAGAGCCCATTTACCCGTTATTTCATTCAACCCCAAAGATCCTAATAGTTATATA

ch3_cgd8_2370 (541) TGTACAAGAGCCCATTTACCCGTTATTTCATTCAACCCCAAAGATCCTAATAGTTATATA

ch4_cgd8_2370 (541) TGTACAAGAGCCCATTTACCCGTTATTTCATTCAACCCCAAAGATCCTAATAGTTATATA

TU502_cgd8_2370 (541) TGTACAAGAGCCCATTTACCCGTTATTTCATTCAACCCCAAAGATCCTAATAGTTATATA

rabbit1_cgd8_2370 (541) TGTACAAGAGCCCATTTACCCGTTATTTCATTCAACCCCAAAGATCCTAATAGTTATATA

rabbit2_cgd8_2370 (541) TGTACAAGAGCCCATTTACCCGTTATTTCATTCAACCCCAAAGATCCTAATAGTTATATA

rabbit3_cgd8_2370 (541) TGTACAAGAGCCCATTTACCCGTTATTTCATTCAACCCCAAAGATCCTAATAGTTATATA

rabbit4_cgd8_2370 (541) TGTACAAGAGCCCATTTACCCGTTATTTCATTCAACCCCAAAGATCCTAATAGTTATATA

cp2_cgd8_2370 (541) TGCACAAGAGCCCATTTACCCGTTATTTCATTCAACCCTAAAGATCCTAATAGTTGTATA

cp3_cgd8_2370 (541) TGCACAAGAGCCCATTTACCCGTTATTTCATTCAACCCTAAAGATCCTAATAGTTGTATA

cp4_cgd8_2370 (541) TGCACAAGAGCCCATTTACCCGTTATTTCATTCAACCCTAAAGATCCTAATAGTTGTATA

Iowa_cgd8_2370 (541) TGCACAAGAGCCCATTTACCCGTTATTTCATTCAACCCTAAAGATCCTAATAGTTGTATA

Moredun_cgd8_2370 (541) TGCACAAGAGCCCATTTACCCGTTATTTCATTCAACCCTAAAGATCCTAATAGTTGTATA

w65_cgd8_2370 (541) TGCACAAGAGCCCATTTACCCGTTATTTCATTCAACCCTAAAGATCCTAATAGTTGTATA

w66_cgd8_2370 (541) TGCACAAGAGCCCATTTACCCGTTATTTCATTCAACCCTAAAGATCCTAATAGTTGTATA

w67_cgd8_2370 (541) TGCACAAGAGCCCATTTACCCGTTATTTCATTCAACCCTAAAGATCCTAATAGTTGTATA

w70_cgd8_2370 (541) TGCACAAGAGCCCATTTACCCGTTATTTCATTCAACCCTAAAGATCCTAATAGTTGTATA

c.meleagridis_cgd8_2370 (541) TGTACAAGAGCTCATTTACCCGTTATTTCATTCAACCCTAAAGATCCTAATGGCTATATA

601 660

ch2_cgd8_2370 (601) AAATATCATGAATGTATTCACGTCCCTAAAGAAAGGCTCATTGATGTTAATGGCTGTGGG

ch3_cgd8_2370 (601) AAATATCATGAATGTATTCACGTCCCTAAAGAAAGGCTCATTGATGTTAATGGCTGTGGG

ch4_cgd8_2370 (601) AAATATCATGAATGTATTCACGTCCCTAAAGAAAGGCTCATTGATGTTAATGGCTGTGGG

TU502_cgd8_2370 (601) AAATATCATGAATGTATTCACGTCCCTAAAGAAAGGCTCATTGATGTTAATGGCTGTGGG

rabbit1_cgd8_2370 (601) AAATATCATGAATGTATTCACGTCCCTAAAGAAAGGCTCATTGATGTTAATGGCTGTGGG

rabbit2_cgd8_2370 (601) AAATATCATGAATGTATTCACGTCCCTAAAGAAAGGCTCATTGATGTTAATGGCTGTGGG

rabbit3_cgd8_2370 (601) AAATATCATGAATGTATTCACGTCCCTAAAGAAAGGCTCATTGATGTTAATGGCTGTGGG

rabbit4_cgd8_2370 (601) AAATATCATGAATGTATTCACGTCCCTAAAGAAAGGCTCATTGATGTTAATGGCTGTGGG

cp2_cgd8_2370 (601) AAATATCATGAATGTATTCACGTCCCTAAAGAAAGGCTCATTGATGTTAATGGCTGTGGG

cp3_cgd8_2370 (601) AAATATCATGAATGTATTCACGTCCCTAAAGAAAGGCTCATTGATGTTAATGGCTGTGGG

cp4_cgd8_2370 (601) AAATATCATGAATGTATTCACGTCCCTAAAGAAAGGCTCATTGATGTTAATGGCTGTGGG

Iowa_cgd8_2370 (601) AAATATCATGAATGTATTCACGTCCCTAAAGAAAGGCTCATTGATGTTAATGGCTGTGGG

Moredun_cgd8_2370 (601) AAATATCATGAATGTATTCACGTCCCTAAAGAAAGGCTCATTGATGTTAATGGCTGTGGG

w65_cgd8_2370 (601) AAATATCATGAATGTATTCACGTCCCTAAAGAAAGGCTCATTGATGTTAATGGCTGTGGG

w66_cgd8_2370 (601) AAATATCATGAATGTATTCACGTCCCTAAAGAAAGGCTCATTGATGTTAATGGCTGTGGG

w67_cgd8_2370 (601) AAATATCATGAATGTATTCACGTCCCTAAAGAAAGGCTCATTGATGTTAATGGCTGTGGG

w70_cgd8_2370 (601) AAATATCATGAATGTATTCACGTCCCTAAAGAAAGGCTCATTGATGTTAATGGCTGTGGG

c.meleagridis_cgd8_2370 (601) AAATATCATGAATGTATTCACGTCCCTAAAGAAAAGCTCGTTGATGTTAATGGCTGTGGG

661

ch2_cgd8_2370 (661) G

ch3_cgd8_2370 (661) G

ch4_cgd8_2370 (661) G

TU502_cgd8_2370 (661) G

rabbit1_cgd8_2370 (661) G

rabbit2_cgd8_2370 (661) G

rabbit3_cgd8_2370 (661) G

rabbit4_cgd8_2370 (661) G

cp2_cgd8_2370 (661) G

cp3_cgd8_2370 (661) G

cp4_cgd8_2370 (661) G

Iowa_cgd8_2370 (661) G

Moredun_cgd8_2370 (661) G

w65_cgd8_2370 (661) G

w66_cgd8_2370 (661) G

w67_cgd8_2370 (661) G

w70_cgd8_2370 (661) G

c.meleagridis_cgd8_2370 (661) G

**Cgd2_2430 gene PCR products**

1 60

ch2_cgd2_2430 (1) ACCTATAATTGATCGAAAAAGTGTTTTCCAAGCTCATGCATGTAAGGTAGAAACAGTTGA

ch3_cgd2_2430 (1) ACCTATAATTGATCGAAAAAGTGTTTTCCAAGCTCATGCATGTAAGGTAGAAACAGTTGA

ch4_cgd2_2430 (1) ACCTATAATTGATCGAAAAAGTGTTTTCCAAGCTCATGCATGTAAGGTAGAAACAGTTGA

TU502_cgd2_2430 (1) ACCTATAATTGATCGAAAAAGTGTTTTCCAAGCTCATGCATGTAAGGTAGAAACAGTTGA

rabbit1_cgd2_2430 (1) ACCTATAATTGATCGAAAAAGTGTTTTCCAAGCTCATGCGTGTAAGGTAGAAACAGTTGA

rabbit2_cgd2_2430 (1) ACCTATAATTGATCGAAAAAGTGTTTTCCAAGCTCATGCGTGTAAGGTAGAAACAGTTGA

rabbit3_cgd2_2430 (1) ACCTATAATTGATCGAAAAAGTGTTTTCCAAGCTCATGCGTGTAAGGTAGAAACAGTTGA

rabbit4_cgd2_2430 (1) ACCTATAATTGATCGAAAAAGTGTTTTCCAAGCTCATGCGTGTAAGGTAGAAACAGTTGA

cp2_cgd2_2430 (1) ACCTATAATTGATCGAAAAAGTGTTTTCCAAGCTCATGCGTGTAAGGTAGAAACAGTTGA

cp3_cgd2_2430 (1) ACCTATAATTGATCGAAAAAGTGTTTTCCAAGCTCATGCGTGTAAGGTAGAAACAGTTGA

cp4_cgd2_2430 (1) ACCTATAATTGATCGAAAAAGTGTTTTCCAAGCTCATGCGTGTAAGGTAGAAACAGTTGA

Iowa_cgd2_2430 (1) ACCTATAATTGATCGAAAAAGTGTTTTCCAAGCTCATGCGTGTAAGGTAGAAACAGTTGA

Moredun_cgd2_2430 (1) ACCTATAATTGATCGAAAAAGTGTTTTCCAAGCTCATGCGTGTAAGGTAGAAACAGTTGA

w65_cgd2_2430 (1) ACCTATAATTGATCGAAAAAGTGTTTTCCAAGCTCATGCGTGTAAGGTAGAAACAGTTGA

w66_cgd2_2430 (1) ACCTATAATTGATCGAAAAAGTGTTTTCCAAGCTCATGCGTGTAAGGTAGAAACAGTTGA

w67_cgd2_2430 (1) ACCTATAATTGATCGAAAAAGTGTTTTCCAAGCTCATGCGTGTAAGGTAGAAACAGTTGA

w70_cgd2_2430 (1) ACCTATAATTGATCGAAAAAGTGTTTTCCAAGCTCATGCGTGTAAGGTAGAAACAGTTGA

61 120

ch2_cgd2_2430 (61) ACAAGTTAAAAAGATTATTAAATGGTTGCTTTCAAACCCAAAGATTGCTAAAGCAACGCA

ch3_cgd2_2430 (61) ACAAGTTAAAAAGATTATTAAATGGTTGCTTTCAAACCCAAAGATTGCTAAAGCAACGCA

ch4_cgd2_2430 (61) ACAAGTTAAAAAGATTATTAAATGGTTGCTTTCAAACCCAAAGATTGCTAAAGCAACGCA

TU502_cgd2_2430 (61) ACAAGTTAAAAAGATTATTAAATGGTTGCTTTCAAACCCAAAGATTGCTAAAGCAACGCA

rabbit1_cgd2_2430 (61) ACAAGTTAAAAAGATTATTAAATGGTTGCTTTCAAACCCAAAGATTGCTAAAGCAACGCA

rabbit2_cgd2_2430 (61) ACAAGTTAAAAAGATTATTAAATGGTTGCTTTCAAACCCAAAGATTGCTAAAGCAACGCA

rabbit3_cgd2_2430 (61) ACAAGTTAAAAAGATTATTAAATGGTTGCTTTCAAACCCAAAGATTGCTAAAGCAACGCA

rabbit4_cgd2_2430 (61) ACAAGTTAAAAAGATTATTAAATGGTTGCTTTCAAACCCAAAGATTGCTAAAGCAACGCA

cp2_cgd2_2430 (61) ACAAGTTAAAAAAGTTATCAAATGGTTGCTTTCGAACCCAAAGATTGCTAAAGCAACGCA

cp3_cgd2_2430 (61) ACAAGTTAAAAAAGTTATCAAATGGTTGCTTTCGAACCCAAAGATTGCTAAAGCAACGCA

cp4_cgd2_2430 (61) ACAAGTTAAAAAAGTTATCAAATGGTTGCTTTCGAACCCAAAGATTGCTAAAGCAACGCA

Iowa_cgd2_2430 (61) ACAAGTTAAAAAAGTTATCAAATGGTTGCTTTCGAACCCAAAGATTGCTAAAGCAACGCA

Moredun_cgd2_2430 (61) ACAAGTTAAAAAAGTTATCAAATGGTTGCTTTCGAACCCAAAGATTGCTAAAGCAACGCA

w65_cgd2_2430 (61) ACAAGTTAAAAAAGTTATCAAATGGTTGCTTTCGAACCCAAAGATTGCTAAAGCAACGCA

w66_cgd2_2430 (61) ACAAGTTAAAAAAGTTATCAAATGGTTGCTTTCGAACCCAAAGATTGCTAAAGCAACGCA

w67_cgd2_2430 (61) ACAAGTTAAAAAAGTTATCAAATGGTTGCTTTCGAACCCAAAGATTGCTAAAGCAACGCA

w70_cgd2_2430 (61) ACAAGTTAAAAAAGTTATCAAATGGTTGCTTTCGAACCCAAAGATTGCTAAAGCAACGCA

121 180

ch2_cgd2_2430 (121) CAATATTTGGTCATATAGAATATTCAAAGAGAAAAATTTCGCAGAAGTATCTGAAGGATC

ch3_cgd2_2430 (121) CAATATTTGGTCATATAGAATATTCAAAGAGAAAAATTTCGCAGAAGTATCTGAAGGATC

ch4_cgd2_2430 (121) CAATATTTGGTCATATAGAATATTCAAAGAGAAAAATTTCGCAGAAGTATCTGAAGGATC

TU502_cgd2_2430 (121) CAATATTTGGTCATATAGAATATTCAAAGAGAAAAATTTCGCAGAAGTATCTGAAGGATC

rabbit1_cgd2_2430 (121) CAATATTTGGTCATATAGAATATTCAAGGAGAAAAATTTCGCAGAAGTATCTGAAGGATC

rabbit2_cgd2_2430 (121) CAATATTTGGTCATATAGAATATTCAAGGAGAAAAATTTCGCAGAAGTATCTGAAGGATC

rabbit3_cgd2_2430 (121) CAATATTTGGTCATATAGAATATTCAAGGAGAAAAATTTCGCAGAAGTATCTGAAGGATC

rabbit4_cgd2_2430 (121) CAATATTTGGTCATATAGAATATTCAAGGAGAAAAATTTCGCAGAAGTATCTGAAGGATC

cp2_cgd2_2430 (121) CAATATTTGGTCATATAGAATATTCAAGGAGAAAAATTTCGCAGAAGTAACTGAAGGATC

cp3_cgd2_2430 (121) CAATATTTGGTCATATAGAATATTCAAGGAGAAAAATTTCGCAGAAGTAACTGAAGGATC

cp4_cgd2_2430 (121) CAATATTTGGTCATATAGAATATTCAAGGAGAAAAATTTCGCAGAAGTAACTGAAGGATC

Iowa_cgd2_2430 (121) CAATATTTGGTCATATAGAATATTCAAGGAGAAAAATTTCGCAGAAGTAACTGAAGGATC

Moredun_cgd2_2430 (121) CAATATTTGGTCATATAGAATATTCAAGGAGAAAAATTTCGCAGAAGTAACTGAAGGATC

w65_cgd2_2430 (121) CAATATTTGGTCATATAGAATATTCAAGGAGAAAAATTTCGCAGAAGTAACTGAAGGATC

w66_cgd2_2430 (121) CAATATTTGGTCATATAGAATATTCAAGGAGAAAAATTTCGCAGAAGTAACTGAAGGATC

w67_cgd2_2430 (121) CAATATTTGGTCATATAGAATATTCAAGGAGAAAAATTTCGCAGAAGTAACTGAAGGATC

w70_cgd2_2430 (121) CAATATTTGGTCATATAGAATATTCAAGGAGAAAAATTTCGCAGAAGTAACTGAAGGATC

181 240

ch2_cgd2_2430 (181) TTTCCCCATTGGGTACGATATCATATCACAAGATCATGACTCGGATGGCGAGAATGCAGC

ch3_cgd2_2430 (181) TTTCCCCATTGGGTACGATATCATATCACAAGATCATGACTCGGATGGCGAGAATGCAGC

ch4_cgd2_2430 (181) TTTCCCCATTGGGTACGATATCATATCACAAGATCATGACTCGGATGGCGAGAATGCAGC

TU502_cgd2_2430 (181) TTTCCCCATTGGGTACGATATCATATCACAAGATCATGACTCGGATGGCGAGAATGCAGC

rabbit1_cgd2_2430 (181) TTTCCCCATCGGGTACGATATCATATCACAAGATCATGACTCGGATGGCGAGAATGCAGC

rabbit2_cgd2_2430 (181) TTTCCCCATCGGGTACGATATCATATCACAAGATCATGACTCGGATGGCGAGAATGCAGC

rabbit3_cgd2_2430 (181) TTTCCCCATCGGGTACGATATCATATCACAAGATCATGACTCGGATGGCGAGAATGCAGC

rabbit4_cgd2_2430 (181) TTTCCCCATCGGGTACGATATCATATCACAAGATCATGACTCGGATGGCGAGAATGCAGC

cp2_cgd2_2430 (181) TTTCCCCATTGGGTACGATATCATATCACAAGATCACGACTCGGATGGCGAGAATGCAGC

cp3_cgd2_2430 (181) TTTCCCCATTGGGTACGATATCATATCACAAGATCACGACTCGGATGGCGAGAATGCAGC

cp4_cgd2_2430 (181) TTTCCCCATTGGGTACGATATCATATCACAAGATCACGACTCGGATGGCGAGAATGCAGC

Iowa_cgd2_2430 (181) TTTCCCCATTGGGTACGATATCATATCACAAGATCACGACTCGGATGGCGAGAATGCAGC

Moredun_cgd2_2430 (181) TTTCCCCATTGGGTACGATATCATATCACAAGATCACGACTCGGATGGCGAGAATGCAGC

w65_cgd2_2430 (181) TTTCCCCATTGGGTACGATATCATATCACAAGATCACGACTCGGATGGCGAGAATGCAGC

w66_cgd2_2430 (181) TTTCCCCATTGGGTACGATATCATATCACAAGATCACGACTCGGATGGCGAGAATGCAGC

w67_cgd2_2430 (181) TTTCCCCATTGGGTACGATATCATATCACAAGATCACGACTCGGATGGCGAGAATGCAGC

w70_cgd2_2430 (181) TTTCCCCATTGGGTACGATATCATATCACAAGATCACGACTCGGATGGCGAGAATGCAGC

241 300

ch2_cgd2_2430 (241) AGGAGGCAGACTTCAACATCTTCTTGAAATTACTAATGCAAAGAATGTATTCGTCATGGT

ch3_cgd2_2430 (241) AGGAGGCAGACTTCAACATCTTCTTGAAATTACTAATGCAAAGAATGTATTCGTCATGGT

ch4_cgd2_2430 (241) AGGAGGCAGACTTCAACATCTTCTTGAAATTACTAATGCAAAGAATGTATTCGTCATGGT

TU502_cgd2_2430 (241) AGGAGGCAGACTTCAACATCTTCTTGAAATTACTAATGCAAAGAATGTATTCGTCATGGT

rabbit1_cgd2_2430 (241) AGGAGGTAGACTTCAACATCTTCTTGAAATTACTAATGCAAAGAATGTATTCGTCATGGT

rabbit2_cgd2_2430 (241) AGGAGGTAGACTTCAACATCTTCTTGAAATTACTAATGCAAAGAATGTATTCGTCATGGT

rabbit3_cgd2_2430 (241) AGGAGGTAGACTTCAACATCTTCTTGAAATTACTAATGCAAAGAATGTATTCGTCATGGT

rabbit4_cgd2_2430 (241) AGGAGGTAGACTTCAACATCTTCTTGAAATTACTAATGCAAAGAATGTATTCGTCATGGT

cp2_cgd2_2430 (241) AGGAGGCAGACTTCAACATCTTCTTGAAATTACTAATGCAAAGAATGTATTCGTTATGGT

cp3_cgd2_2430 (241) AGGAGGCAGACTTCAACATCTTCTTGAAATTACTAATGCAAAGAATGTATTCGTTATGGT

cp4_cgd2_2430 (241) AGGAGGCAGACTTCAACATCTTCTTGAAATTACTAATGCAAAGAATGTATTCGTTATGGT

Iowa_cgd2_2430 (241) AGGAGGCAGACTTCAACATCTTCTTGAAATTACTAATGCAAAGAATGTATTCGTTATGGT

Moredun_cgd2_2430 (241) AGGAGGCAGACTTCAACATCTTCTTGAAATTACTAATGCAAAGAATGTATTCGTTATGGT

w65_cgd2_2430 (241) AGGAGGCAGACTTCAACATCTTCTTGAAATTACTAATGCAAAGAATGTATTCGTTATGGT

w66_cgd2_2430 (241) AGGAGGCAGACTTCAACATCTTCTTGAAATTACTAATGCAAAGAATGTATTCGTTATGGT

w67_cgd2_2430 (241) AGGAGGCAGACTTCAACATCTTCTTGAAATTACTAATGCAAAGAATGTATTCGTTATGGT

w70_cgd2_2430 (241) AGGAGGCAGACTTCAACATCTTCTTGAAATTACTAATGCAAAGAATGTATTCGTTATGGT

301 360

ch2_cgd2_2430 (301) GTCTAGATGGTACGGGGGTGTTCAACTAGGGCCAGACAGGTTTAAGCATATTAATAATGC

ch3_cgd2_2430 (301) GTCTAGATGGTACGGGGGTGTTCAACTAGGGCCAGACAGGTTTAAGCATATTAATAATGC

ch4_cgd2_2430 (301) GTCTAGATGGTACGGGGGTGTTCAACTAGGGCCAGACAGGTTTAAGCATATTAATAATGC

TU502_cgd2_2430 (301) GTCTAGATGGTACGGGGGTGTTCAACTAGGGCCAGACAGGTTTAAGCATATTAATAATGC

rabbit1_cgd2_2430 (301) GTCTAGATGGTACGGGGGTGTTCAACTAGGGCCAGACAGGTTTAAGCATATTAATAATGC

rabbit2_cgd2_2430 (301) GTCTAGATGGTACGGGGGTGTTCAACTAGGGCCAGACAGGTTTAAGCATATTAATAATGC

rabbit3_cgd2_2430 (301) GTCTAGATGGTACGGGGGTGTTCAACTAGGGCCAGACAGGTTTAAGCATATTAATAATGC

rabbit4_cgd2_2430 (301) GTCTAGATGGTACGGGGGTGTTCAACTAGGGCCAGACAGGTTTAAGCATATTAATAATGC

cp2_cgd2_2430 (301) GTCTAGATGGTACGGGGGTATTCAACTAGGGCCAGACAGGTTTAAACATATTAATAATGC

cp3_cgd2_2430 (301) GTCTAGATGGTACGGGGGTATTCAACTAGGGCCAGACAGGTTTAAACATATTAATAATGC

cp4_cgd2_2430 (301) GTCTAGATGGTACGGGGGTATTCAACTAGGGCCAGACAGGTTTAAACATATTAATAATGC

Iowa_cgd2_2430 (301) GTCTAGATGGTACGGGGGTATTCAACTAGGGCCAGACAGGTTTAAACATATTAATAATGC

Moredun_cgd2_2430 (301) GTCTAGATGGTACGGGGGTATTCAACTAGGGCCAGACAGGTTTAAACATATTAATAATGC

w65_cgd2_2430 (301) GTCTAGATGGTACGGGGGTATTCAACTAGGGCCAGACAGGTTTAAACATATTAATAATGC

w66_cgd2_2430 (301) GTCTAGATGGTACGGGGGTATTCAACTAGGGCCAGACAGGTTTAAACATATTAATAATGC

w67_cgd2_2430 (301) GTCTAGATGGTACGGGGGTATTCAACTAGGGCCAGACAGGTTTAAACATATTAATAATGC

w70_cgd2_2430 (301) GTCTAGATGGTACGGGGGTATTCAACTAGGGCCAGACAGGTTTAAACATATTAATAATGC

361

ch2_cgd2_2430 (361) T

ch3_cgd2_2430 (361) T

ch4_cgd2_2430 (361) T

TU502_cgd2_2430 (361) T

rabbit1_cgd2_2430 (361) T

rabbit2_cgd2_2430 (361) T

rabbit3_cgd2_2430 (361) T

rabbit4_cgd2_2430 (361) T

cp2_cgd2_2430 (361) T

cp3_cgd2_2430 (361) T

cp4_cgd2_2430 (361) T

Iowa_cgd2_2430 (361) T

Moredun_cgd2_2430 (361) T

w65_cgd2_2430 (361) T

w66_cgd2_2430 (361) T

w67_cgd2_2430 (361) T

w70_cgd2_2430 (361) T

**Chro.30149 gene PCR products**

1 60

ch2_chro.30149 (1) CAGGTGGTGTAACTAGAGAATGGTATAATATTCTAGCTAGAGAAATGTTCAATCCTGATT

ch3_chro.30149 (1) CAGGTGGTGTAACTAGAGAATGGTATAATATTCTAGCTAGAGAAATGTTCAATCCTGATT

ch4_chro.30149 (1) CAGGTGGTGTAACTAGAGAATGGTATAATATTCTAGCTAGAGAAATGTTCAATCCTGATT

TU502_chro.30149 (1) CAGGTGGTGTAACTAGAGAATGGTATAATATTCTAGCTAGAGAAATGTTCAATCCTGATT

rabbit1_chro.30149 (1) CAGGTGGTGTAACTAGAGAATGGTATAATATTCTAGCTAGAGAAATGTTCAATCCTGATT

rabbit2_chro.30149 (1) CAGGTGGTGTAACTAGAGAATGGTATAATATTCTAGCTAGAGAAATGTTCAATCCTGATT

rabbit3_chro.30149 (1) CAGGTGGTGTAACTAGAGAATGGTATAATATTCTAGCTAGAGAAATGTTCAATCCTGATT

rabbit4_chro.30149 (1) CAGGTGGTGTAACTAGAGAATGGTATAATATTCTAGCTAGAGAAATGTTCAATCCTGATT

Iowa_chro.30149 (1) CAGGTGGTGTAACTAGAGAATGGTATAATATTCTAGCTAGAGAAATGTTCAATCCTGATT

moredun_chro.30149 (1) CAGGTGGTGTAACTAGAGAATGGTATAATATTCTAGCTAGAGAAATGTTCAATCCTGATT

cp2_chro.30149 (1) CAGGTGGTGTAACTAGAGAATGGTATAATATTCTAGCTAGAGAAATGTTCAATCCTGATT

cp3_chro.30149 (1) CAGGTGGTGTAACTAGAGAATGGTATAATATTCTAGCTAGAGAAATGTTCAATCCTGATT

c.meleagridis_chro.30149 (1) CAGGTGGTGTAACAAGGGAATGGTATAATATTCTAGCTAGGGAGATGTTTAATCCCGACT

61 120

ch2_chro.30149 (61) ATGCTTTATTTAGAAGAGAAGGATCAAAGAGTGAATTTAACCATCCAAATCCATTAAGTT

ch3_chro.30149 (61) ATGCTTTATTTAGAAGAGAAGGATCAAAGAGTGAATTTAACCATCCAAATCCATTAAGTT

ch4_chro.30149 (61) ATGCTTTATTTAGAAGAGAAGGATCAAAGAGTGAATTTAACCATCCAAATCCATTAAGTT

TU502_chro.30149 (61) ATGCTTTATTTAGAAGAGAAGGATCAAAGAGTGAATTTAACCATCCAAATCCATTAAGTT

rabbit1_chro.30149 (61) ATGCTTTATTTAGAAGAGAAGGATCAAAGAGTGAATTTAACCATCCAAATCCATTAAGTT

rabbit2_chro.30149 (61) ATGCTTTATTTAGAAGAGAAGGATCAAAGAGTGAATTTAACCATCCAAATCCATTAAGTT

rabbit3_chro.30149 (61) ATGCTTTATTTAGAAGAGAAGGATCAAAGAGTGAATTTAACCATCCAAATCCATTAAGTT

rabbit4_chro.30149 (61) ATGCTTTATTTAGAAGAGAAGGATCAAAGAGTGAATTTAACCATCCAAATCCATTAAGTT

Iowa_chro.30149 (61) ATGCTTTATTTAGAAGAGAAGGATCAAAGAGTGAATTTAACCATCCAAATCCATTAAGTT

moredun_chro.30149 (61) ATGCTTTATTTAGAAGAGAAGGATCAAAGAGTGAATTTAACCATCCAAATCCATTAAGTT

cp2_chro.30149 (61) ATGCTTTATTTAGAAGAGAAGGATCAAAGAGTGAATTTAACCATCCAAATCCATTAAGTT

cp3_chro.30149 (61) ATGCTTTATTTAGAAGAGAAGGATCAAAGAGTGAATTTAACCATCCAAATCCATTAAGTT

c.meleagridis_chro.30149 (61) ACGCTTTGTTCAGAAGAGAAGGATCAAAGAGTGAATTTAACCATCCAAACCCATTAAGTT

121 180

ch2_chro.30149 (121) ATATAAATGCTGATCATCTTCATTTCTTTAAATTTATTGGACGTATAATAGGGAAATGTA

ch3_chro.30149 (121) ATATAAATGCTGATCATCTTCATTTCTTTAAATTTATTGGACGTATAATAGGGAAATGTA

ch4_chro.30149 (121) ATATAAATGCTGATCATCTTCATTTCTTTAAATTTATTGGACGTATAATAGGGAAATGTA

TU502_chro.30149 (121) ATATAAATGCTGATCATCTTCATTTCTTTAAATTTATTGGACGTATAATAGGGAAATGTA

rabbit1_chro.30149 (121) ATATAAATGCTGATCATCTTCATTTCTTTAAATTTATTGGACGTATAATAGGGAAATGTA

rabbit2_chro.30149 (121) ATATAAATGCTGATCATCTTCATTTCTTTAAATTTATTGGACGTATAATAGGGAAATGTA

rabbit3_chro.30149 (121) ATATAAATGCTGATCATCTTCATTTCTTTAAATTTATTGGACGTATAATAGGGAAATGTA

rabbit4_chro.30149 (121) ATATAAATGCTGATCATCTTCATTTCTTTAAATTTATTGGACGTATAATAGGGAAATGTA

Iowa_chro.30149 (121) ATATAAATGCTGATCATCTTCATTTCTTTAAATTTATTGGACGTATAATAGGGAAATGTA

moredun_chro.30149 (121) ATATAAATGCTGATCATCTTCATTTCTTTAAATTTATTGGACGTATAATAGGGAAATGTA

cp2_chro.30149 (121) ATATAAATGCTGATCATCTTCATTTCTTTAAATTTATTGGACGTATAATAGGGAAATGTA

cp3_chro.30149 (121) ATATAAATGCTGATCATCTTCATTTCTTTAAATTTATTGGACGTATAATAGGGAAATGTA

c.meleagridis_chro.30149 (121) ATATAAATGCTGATCATCTTCATTTCTTTAAGTTTATCGGACGTATCATAGGTAAATGCA

181 240

ch2_chro.30149 (181) TTTATGATGGTCAACATTTGGATGCATGGTTTACTCGATCATTTTATAAGAATATGTTAG

ch3_chro.30149 (181) TTTATGATGGTCAACATTTGGATGCATGGTTTACTCGATCATTTTATAAGAATATGTTAG

ch4_chro.30149 (181) TTTATGATGGTCAACATTTGGATGCATGGTTTACTCGATCATTTTATAAGAATATGTTAG

TU502_chro.30149 (181) TTTATGATGGTCAACATTTGGATGCATGGTTTACTCGATCATTTTATAAGAATATGTTAG

rabbit1_chro.30149 (181) TTTATGATGGTCAACATTTGGATGCATGGTTTACTCGATCATTTTATAAGAATATGTTAG

rabbit2_chro.30149 (181) TTTATGATGGTCAACATTTGGATGCATGGTTTACTCGATCATTTTATAAGAATATGTTAG

rabbit3_chro.30149 (181) TTTATGATGGTCAACATTTGGATGCATGGTTTACTCGATCATTTTATAAGAATATGTTAG

rabbit4_chro.30149 (181) TTTATGATGGTCAACATTTGGATGCATGGTTTACTCGATCATTTTATAAGAATATGTTAG

Iowa_chro.30149 (181) TTTATGATGGTCAACATTTGGATGCATGGTTTACTCGATCATTTTATAAGAATATGTTAG

moredun_chro.30149 (181) TTTATGATGGTCAACATTTGGATGCATGGTTTACTCGATCATTTTATAAGAATATGTTAG

cp2_chro.30149 (181) TTTATGATGGTCAACATTTGGATGCATGGTTTACTCGATCATTTTATAAGAATATGTTAG

cp3_chro.30149 (181) TTTATGATGGTCAACATTTGGATGCATGGTTTACTCGATCATTTTATAAGAATATGTTAG

c.meleagridis_chro.30149 (181) TTTATGATGGTCAACATTTGGATGCATGGTTTACTCGATCATTTTATAAGAACATGTTAG

241 300

ch2_chro.30149 (241) GACAACCAATAACACCATCAGATGCAGAATCAATAGATCCTGAACTTTATAAGAATTTGA

ch3_chro.30149 (241) GACAACCAATAACACCATCAGATGCAGAATCAATAGATCCTGAACTTTATAAGAATTTGA

ch4_chro.30149 (241) GACAACCAATAACACCATCAGATGCAGAATCAATAGATCCTGAACTTTATAAGAATTTGA

TU502_chro.30149 (241) GACAACCAATAACACCATCAGATGCAGAATCAATAGATCCTGAACTTTATAAGAATTTGA

rabbit1_chro.30149 (241) GACAACCAATAACACCATCAGATGCAGAATCAATAGATCCTGAACTTTATAAGAATTTGA

rabbit2_chro.30149 (241) GACAACCAATAACACCATCAGATGCAGAATCAATAGATCCTGAACTTTATAAGAATTTGA

rabbit3_chro.30149 (241) GACAACCAATAACACCATCAGATGCAGAATCAATAGATCCTGAACTTTATAAGAATTTGA

rabbit4_chro.30149 (241) GACAACCAATAACACCATCAGATGCAGAATCAATAGATCCTGAACTTTATAAGAATTTGA

Iowa_chro.30149 (241) GACAACCAATAACACCATCAGATGCAGAATCAATAGATCCTGAACTTTATAAGAATTTGA

moredun_chro.30149 (241) GACAACCAATAACACCATCAGATGCAGAATCAATAGATCCTGAACTTTATAAGAATTTGA

cp2_chro.30149 (241) GACAACCAATAACACCATCAGATGCAGAATCAATAGATCCTGAACTTTATAAGAATTTGA

cp3_chro.30149 (241) GACAACCAATAACACCATCAGATGCAGAATCAATAGATCCTGAACTTTATAAGAATTTGA

c.meleagridis_chro.30149 (241) GACAGCCAATAACACCGTCAGACGCAGAATCAATAGATCCTGAACTTTATAAGAATTTGA

301

ch2_chro.30149 (301) ATGTAATG

ch3_chro.30149 (301) ATGTAATG

ch4_chro.30149 (301) ATGTAATG

TU502_chro.30149 (301) ATGTAATG

rabbit1_chro.30149 (301) ATGTAATG

rabbit2_chro.30149 (301) ATGTAATG

rabbit3_chro.30149 (301) ATGTAATG

rabbit4_chro.30149 (301) ATGTAATG

Iowa_chro.30149 (301) ATGTAATG

moredun_chro.30149 (301) ATGTAATG

cp2_chro.30149 (301) ATGTAATG

cp3_chro.30149 (301) ATGTAATG

c.meleagridis_chro.30149 (301) ATGTAATG

**Chro.20156 gene PCR products**

1 60

ch2_chro.20156 (1) TTTCGCTTGAAGCCGTAAACTTTAGGATGTTACCAAGGGCAGAGTCTCAAAAAGAATGTA

ch3_chro.20156 (1) TTTCGCTTGAAGCCGTAAACTTTAGGATGTTACCAAGGGCAGAGTCTCAAAAAGAATGTA

ch4_chro.20156 (1) TTTCGCTTGAAGCCGTAAACTTTAGGATGTTACCAAGGGCAGAGTCTCAAAAAGAATGTA

TU502_chro.20156 (1) TTTCGCTTGAAGCCGTAAACTTTAGGATGTTACCAAGGGCAGAGTCTCAAAAAGAATGTA

rabbit1_chro.20156 (1) TTTCGCTTGAAGCCGTAAACTTTAGGATGTTACCAAGGGCAGAGTCTCAAAAAGAATGTA

rabbit2_chro.20156 (1) TTTCGCTTGAAGCCGTAAACTTTAGGATGTTACCAAGGGCAGAGTCTCAAAAAGAATGTA

rabbit3_chro.20156 (1) TTTCGCTTGAAGCCGTAAACTTTAGGATGTTACCAAGGGCAGAGTCTCAAAAAGAATGTA

rabbit4_chro.20156 (1) TTTCGCTTGAAGCCGTAAACTTTAGGATGTTACCAAGGGCAGAGTCTCAAAAAGAATGTA

cp2_chro.20156 (1) TTTCGCTTGAAGCCGTAAACTTTAGGATGTTACCAAGGGCAGAGTCTCAAAAAGAATGTA

cp3_chro.20156 (1) TTTCGCTTGAAGCCGTAAACTTTAGGATGTTACCAAGGGCAGAGTCTCAAAAAGAATGTA

cp4_chro.20156 (1) TTTCGCTTGAAGCCGTAAACTTTAGGATGTTACCAAGGGCAGAGTCTCAAAAAGAATGTA

Iowa_chro.20156 (1) TTTCGCTTGAAGCCGTAAACTTTAGGATGTTACCAAGGGCAGAGTCTCAAAAAGAATGTA

Moredun_chro.20156 (1) TTTCGCTTGAAGCCGTAAACTTTAGGATGTTACCAAGGGCAGAGTCTCAAAAAGAATGTA

w65_chro.20156 (1) TTTCGCTTGAAGCCGTAAACTTTAGGATGTTACCAAGGGCAGAGTCTCAAAAAGAATGTA

w66_chro.20156 (1) TTTCGCTTGAAGCCGTAAACTTTAGGATGTTACCAAGGGCAGAGTCTCAAAAAGAATGTA

w67_chro.20156 (1) TTTCGCTTGAAGCCGTAAACTTTAGGATGTTACCAAGGGCAGAGTCTCAAAAAGAATGTA

w70_chro.20156 (1) TTTCGCTTGAAGCCGTAAACTTTAGGATGTTACCAAGGGCAGAGTCTCAAAAAGAATGTA

61 120

ch2_chro.20156 (61) AACTGCCAAGCGGAAAAAAGAGTAGTAGAATTTTATGTTTTACAACGGCAGGAAGACGGC

ch3_chro.20156 (61) AACTGCCAAGCGGAAAAAAGAGTAGTAGAATTTTATGTTTTACAACGGCAGGAAGACGGC

ch4_chro.20156 (61) AACTGCCAAGCGGAAAAAAGAGTAGTAGAATTTTATGTTTTACAACGGCAGGAAGACGGC

TU502_chro.20156 (61) AACTGCCAAGCGGAAAAAAGAGTAGTAGAATTTTATGTTTTACAACGGCAGGAAGACGGC

rabbit1_chro.20156 (61) AACTGCCAAGCGGAAAAAAGAGTAGTAGAATTTTATGTTTTACAACGGCAGGAAGACGGC

rabbit2_chro.20156 (61) AACTGCCAAGCGGAAAAAAGAGTAGTAGAATTTTATGTTTTACAACGGCAGGAAGACGGC

rabbit3_chro.20156 (61) AACTGCCAAGCGGAAAAAAGAGTAGTAGAATTTTATGTTTTACAACGGCAGGAAGACGGC

rabbit4_chro.20156 (61) AACTGCCAAGCGGAAAAAAGAGTAGTAGAATTTTATGTTTTACAACGGCAGGAAGACGGC

cp2_chro.20156 (61) AACTGCCAAGCGGAAAAAAGAGTAGTAGAATTTTATGTTTTACAACAGCAGGAAGACGGC

cp3_chro.20156 (61) AACTGCCAAGCGGAAAAAAGAGTAGTAGAATTTTATGTTTTACAACAGCAGGAAGACGGC

cp4_chro.20156 (61) AACTGCCAAGCGGAAAAAAGAGTAGTAGAATTTTATGTTTTACAACAGCAGGAAGACGGC

Iowa_chro.20156 (61) AACTGCCAAGCGGAAAAAAGAGTAGTAGAATTTTATGTTTTACAACAGCAGGAAGACGGC

Moredun_chro.20156 (61) AACTGCCAAGCGGAAAAAAGAGTAGTAGAATTTTATGTTTTACAACAGCAGGAAGACGGC

w65_chro.20156 (61) AACTGCCAAGCGGAAAAAAGAGTAGTAGAATTTTATGTTTTACAACAGCAGGAAGACGGC

w66_chro.20156 (61) AACTGCCAAGCGGAAAAAAGAGTAGTAGAATTTTATGTTTTACAACAGCAGGAAGACGGC

w67_chro.20156 (61) AACTGCCAAGCGGAAAAAAGAGTAGTAGAATTTTATGTTTTACAACAGCAGGAAGACGGC

w70_chro.20156 (61) AACTGCCAAGCGGAAAAAAGAGTAGTAGAATTTTATGTTTTACAACAGCAGGAAGACGGC

121 180

ch2_chro.20156 (121) ATATTGATCCAACCGAACAAATGATTGAGACAATGTCAATATCAAATCCTGAAGATATAG

ch3_chro.20156 (121) ATATTGATCCAACCGAACAAATGATTGAGACAATGTCAATATCAAATCCTGAAGATATAG

ch4_chro.20156 (121) ATATTGATCCAACCGAACAAATGATTGAGACAATGTCAATATCAAATCCTGAAGATATAG

TU502_chro.20156 (121) ATATTGATCCAACCGAACAAATGATTGAGACAATGTCAATATCAAATCCTGAAGATATAG

rabbit1_chro.20156 (121) ATATTGATCCAACCGAACAAATGATTGAGACAATGTCAATATCAAATCCTGAAGATATAG

rabbit2_chro.20156 (121) ATATTGATCCAACCGAACAAATGATTGAGACAATGTCAATATCAAATCCTGAAGATATAG

rabbit3_chro.20156 (121) ATATTGATCCAACCGAACAAATGATTGAGACAATGTCAATATCAAATCCTGAAGATATAG

rabbit4_chro.20156 (121) ATATTGATCCAACCGAACAAATGATTGAGACAATGTCAATATCAAATCCTGAAGATATAG

cp2_chro.20156 (121) ATATTGATCCAACCGAACAAATGATTGAGACAATGTCAATATCAAATCCTGAAGATATAG

cp3_chro.20156 (121) ATATTGATCCAACCGAACAAATGATTGAGACAATGTCAATATCAAATCCTGAAGATATAG

cp4_chro.20156 (121) ATATTGATCCAACCGAACAAATGATTGAGACAATGTCAATATCAAATCCTGAAGATATAG

Iowa_chro.20156 (121) ATATTGATCCAACCGAACAAATGATTGAGACAATGTCAATATCAAATCCTGAAGATATAG

Moredun_chro.20156 (121) ATATTGATCCAACCGAACAAATGATTGAGACAATGTCAATATCAAATCCTGAAGATATAG

w65_chro.20156 (121) ATATTGATCCAACCGAACAAATGATTGAGACAATGTCAATATCAAATCCTGAAGATATAG

w66_chro.20156 (121) ATATTGATCCAACCGAACAAATGATTGAGACAATGTCAATATCAAATCCTGAAGATATAG

w67_chro.20156 (121) ATATTGATCCAACCGAACAAATGATTGAGACAATGTCAATATCAAATCCTGAAGATATAG

w70_chro.20156 (121) ATATTGATCCAACCGAACAAATGATTGAGACAATGTCAATATCAAATCCTGAAGATATAG

181 240

ch2_chro.20156 (181) AAGAAAGTCCAGCTCAAGTAAATGAAGTCGAAAACATGGTCCCAGAAAACTTGCCTGGTA

ch3_chro.20156 (181) AAGAAAGTCCAGCTCAAGTAAATGAAGTCGAAAACATGGTCCCAGAAAACTTGCCTGGTA

ch4_chro.20156 (181) AAGAAAGTCCAGCTCAAGTAAATGAAGTCGAAAACATGGTCCCAGAAAACTTGCCTGGTA

TU502_chro.20156 (181) AAGAAAGTCCAGCTCAAGTAAATGAAGTCGAAAACATGGTCCCAGAAAACTTGCCTGGTA

rabbit1_chro.20156 (181) AAGAAAGTCCAGCTCTAGTAAATGAAGTCGAAAACATGGTCCCAGAAAACTTGCCTGGTA

rabbit2_chro.20156 (181) AAGAAAGTCCAGCTCTAGTAAATGAAGTCGAAAACATGGTCCCAGAAAACTTGCCTGGTA

rabbit3_chro.20156 (181) AAGAAAGTCCAGCTCTAGTAAATGAAGTCGAAAACATGGTCCCAGAAAACTTGCCTGGTA

rabbit4_chro.20156 (181) AAGAAAGTCCAGCTCTAGTAAATGAAGTCGAAAACATGGTCCCAGAAAACTTGCCTGGTA

cp2_chro.20156 (181) AAGAAGGTCCAGCTCAAGTAAATGAAGCCGAAAACATGGTCCCAGACCACTTGCCTGGTA

cp3_chro.20156 (181) AAGAAGGTCCAGCTCAAGTAAATGAAGCCGAAAACATGGTCCCAGACCACTTGCCTGGTA

cp4_chro.20156 (181) AAGAAGGTCCAGCTCAAGTAAATGAAGCCGAAAACATGGTCCCAGACCACTTGCCTGGTA

Iowa_chro.20156 (181) AAGAAGGTCCAGCTCAAGTAAATGAAGCCGAAAACATGGTCCCAGACCACTTGCCTGGTA

Moredun_chro.20156 (181) AAGAAGGTCCAGCTCAAGTAAATGAAGCCGAAAACATGGTCCCAGACCACTTGCCTGGTA

w65_chro.20156 (181) AAGAAGGTCCAGCTCAAGTAAATGAAGCCGAAAACATGGTCCCAGACCACTTGCCTGGTA

w66_chro.20156 (181) AAGAAGGTCCAGCTCAAGTAAATGAAGCCGAAAACATGGTCCCAGACCACTTGCCTGGTA

w67_chro.20156 (181) AAGAAGGTCCAGCTCAAGTAAATGAAGCCGAAAACATGGTCCCAGACCACTTGCCTGGTA

w70_chro.20156 (181) AAGAAGGTCCAGCTCAAGTAAATGAAGCCGAAAACATGGTCCCAGACCACTTGCCTGGTA

241

ch2_chro.20156 (241) TCAATGCC

ch3_chro.20156 (241) TCAATGCC

ch4_chro.20156 (241) TCAATGCC

TU502_chro.20156 (241) TCAATGCC

rabbit1_chro.20156 (241) TCAATGCC

rabbit2_chro.20156 (241) TCAATGCC

rabbit3_chro.20156 (241) TCAATGCC

rabbit4_chro.20156 (241) TCAATGCC

cp2_chro.20156 (241) TCAATGCC

cp3_chro.20156 (241) TCAATGCC

cp4_chro.20156 (241) TCAATGCC

Iowa_chro.20156 (241) TCAATGCC

Moredun_chro.20156 (241) TCAATGCC

w65_chro.20156 (241) TCAATGCC

w66_chro.20156 (241) TCAATGCC

w67_chro.20156 (241) TCAATGCC

w70_chro.20156 (241) TCAATGCC

**Chro.50317 gene PCR products**

ch2_chro.50317 (1) GGTCTCGGTCTGATCGCAAAAAAGAACCAAAGAGTGAGGATGACAGTGGAAATAATACTA

ch3_chro.50317 (1) GGTCTCGGTCTGATCGCAAAAAAGAACCAAAGAGTGAGGATGACAGTGGAAATAATACTA

ch4_chro.50317 (1) GGTCTCGGTCTGATCGCAAAAAAGAACCAAAGAGTGAGGATGACAGTGGAAATAATACTA

TU502_chro.50317 (1) GGTCTCGGTCTGATCGCAAAAAAGAACCAAAGAGTGAGGATGACAGTGGAAATAATACTA

rabbit1_chro.50317 (1) GGTCTCGGTCTGATCGCAAAAAAGAACCAAAGAGTGAGGATGACAGTGGAAATAATACTA

rabbit2_chro.50317 (1) GGTCTCGGTCTGATCGCAAAAAAGAACCAAAGAGTGAGGATGACAGTGGAAATAATACTA

rabbit3_chro.50317 (1) GGTCTCGGTCTGATCGCAAAAAAGAACCAAAGAGTGAGGATGACAGTGGAAATAATACTA

rabbit4_chro.50317 (1) GGTCTCGGTCTGATCGCAAAAAAGAACCAAAGAGTGAGGATGACAGTGGAAATAATACTA

cp2_chro.50317 (1) GGTCTCGGCCTGATCGCAAAAAAGAACCAAAGAGTGAGGATGACAGTGGAAATAATACTA

cp3_chro.50317 (1) GGTCTCGGCCTGATCGCAAAAAAGAACCAAAGAGTGAGGATGACAGTGGAAATAATACTA

cp4_chro.50317 (1) GGTCTCGGCCTGATCGCAAAAAAGAACCAAAGAGTGAGGATGACAGTGGAAATAATACTA

Iowa_chro.50317 (1) GGTCTCGGCCTGATCGCAAAAAAGAACCAAAGAGTGAGGATGACAGTGGAAATAATACTA

Moredun_chro.50317 (1) GGTCTCGGCCTGATCGCAAAAAAGAACCAAAGAGTGAGGATGACAGTGGAAATAATACTA

w65_chro.50317 (1) GGTCTCGGCCTGATCGCAAAAAAGAACCAAAGAGTGAGGATGACAGTGGAAATAATACTA

w66_chro.50317 (1) GGTCTCGGCCTGATCGCAAAAAAGAACCAAAGAGTGAGGATGACAGTGGAAATAATACTA

w67_chro.50317 (1) GGTCTCGGCCTGATCGCAAAAAAGAACCAAAGAGTGAGGATGACAGTGGAAATAATACTA

w70_chro.50317 (1) GGTCTCGGCCTGATCGCAAAAAAGAACCAAAGAGTGAGGATGACAGTGGAAATAATACTA

C.meleagridis_chro.50317 (1) -----------------------GAACCAAAGAGCGAGGATGACAATGGAAATAATACTA

61 120

ch2_chro.50317 (61) AAAAGGAAGAAATTGATGATGCAGATAATGATATGAATAATGAAATGGATGGAGATGATA

ch3_chro.50317 (61) AAAAGGAAGAAATTGATGATGCAGATAATGATATGAATAATGAAATGGATGGAGATGATA

ch4_chro.50317 (61) AAAAGGAAGAAATTGATGATGCAGATAATGATATGAATAATGAAATGGATGGAGATGATA

TU502_chro.50317 (61) AAAAGGAAGAAATTGATGATGCAGATAATGATATGAATAATGAAATGGATGGAGATGATA

rabbit1_chro.50317 (61) AAAAGGAAGAAATTGATGATGCAGATAATGATATGAATAATGAAATGGATGGAGATGATA

rabbit2_chro.50317 (61) AAAAGGAAGAAATTGATGATGCAGATAATGATATGAATAATGAAATGGATGGAGATGATA

rabbit3_chro.50317 (61) AAAAGGAAGAAATTGATGATGCAGATAATGATATGAATAATGAAATGGATGGAGATGATA

rabbit4_chro.50317 (61) AAAAGGAAGAAATTGATGATGCAGATAATGATATGAATAATGAAATGGATGGAGATGATA

cp2_chro.50317 (61) AAAAGGAAGAAATTGATGATGTAGATAATGATATGAATAATGAAATGGATGGAGATGATA

cp3_chro.50317 (61) AAAAGGAAGAAATTGATGATGTAGATAATGATATGAATAATGAAATGGATGGAGATGATA

cp4_chro.50317 (61) AAAAGGAAGAAATTGATGATGTAGATAATGATATGAATAATGAAATGGATGGAGATGATA

Iowa_chro.50317 (61) AAAAGGAAGAAATTGATGATGTAGATAATGATATGAATAATGAAATGGATGGAGATGATA

Moredun_chro.50317 (61) AAAAGGAAGAAATTGATGATGTAGATAATGATATGAATAATGAAATGGATGGAGATGATA

w65_chro.50317 (61) AAAAGGAAGAAATTGATGATGTAGATAATGATATGAATAATGAAATGGATGGAGATGATA

w66_chro.50317 (61) AAAAGGAAGAAATTGATGATGTAGATAATGATATGAATAATGAAATGGATGGAGATGATA

w67_chro.50317 (61) AAAAGGAAGAAATTGATGATGTAGATAATGATATGAATAATGAAATGGATGGAGATGATA

w70_chro.50317 (61) AAAAGGAAGAAATTGATGATGTAGATAATGATATGAATAATGAAATGGATGGAGATGATA

C.meleagridis_chro.50317 (38) AAAAAGAAGAGATGGATGATGGAGATAATGATATGAATAATGAAATGGACGGAGATGATA

121 180

ch2_chro.50317 (121) AAGATAAAAACGATGATGATGATAATTCATCGGTAGAATCTTCATCATCAACTTCCGAGG

ch3_chro.50317 (121) AAGATAAAAACGATGATGATGATAATTCATCGGTAGAATCTTCATCATCAACTTCCGAGG

ch4_chro.50317 (121) AAGATAAAAACGATGATGATGATAATTCATCGGTAGAATCTTCATCATCAACTTCCGAGG

TU502_chro.50317 (121) AAGATAAAAACGATGATGATGATAATTCATCGGTAGAATCTTCATCATCAACTTCCGAGG

rabbit1_chro.50317 (121) AAGATAAAAACGATGATGATGATAATTCATCGGTAGAATCTTCATCATCAACTTCCGAGG

rabbit2_chro.50317 (121) AAGATAAAAACGATGATGATGATAATTCATCGGTAGAATCTTCATCATCAACTTCCGAGG

rabbit3_chro.50317 (121) AAGATAAAAACGATGATGATGATAATTCATCGGTAGAATCTTCATCATCAACTTCCGAGG

rabbit4_chro.50317 (121) AAGATAAAAACGATGATGATGATAATTCATCGGTAGAATCTTCATCATCAACTTCCGAGG

cp2_chro.50317 (121) AAGATAAAAACGATGATGATGATAATTCATCGGTAGAATCTTCATCATCAACTTCCGAGG

cp3_chro.50317 (121) AAGATAAAAACGATGATGATGATAATTCATCGGTAGAATCTTCATCATCAACTTCCGAGG

cp4_chro.50317 (121) AAGATAAAAACGATGATGATGATAATTCATCGGTAGAATCTTCATCATCAACTTCCGAGG

Iowa_chro.50317 (121) AAGATAAAAACGATGATGATGATAATTCATCGGTAGAATCTTCATCATCAACTTCCGAGG

Moredun_chro.50317 (121) AAGATAAAAACGATGATGATGATAATTCATCGGTAGAATCTTCATCATCAACTTCCGAGG

w65_chro.50317 (121) AAGATAAAAACGATGATGATGATAATTCATCGGTAGAATCTTCATCATCAACTTCCGAGG

w66_chro.50317 (121) AAGATAAAAACGATGATGATGATAATTCATCGGTAGAATCTTCATCATCAACTTCCGAGG

w67_chro.50317 (121) AAGATAAAAACGATGATGATGATAATTCATCGGTAGAATCTTCATCATCAACTTCCGAGG

w70_chro.50317 (121) AAGATAAAAACGATGATGATGATAATTCATCGGTAGAATCTTCATCATCAACTTCCGAGG

C.meleagridis_chro.50317 (98) AAGATGAAAACGAAGATGATGATAATTCATCTTTAGAATCTTCATCATCAACTTCCGAGG

181 240

ch2_chro.50317 (181) AGGAAAATGGTAAAGATGATAGTGATGAAGAAACTGAAATGAATAATGGAAAATTTACTG

ch3_chro.50317 (181) AGGAAAATGGTAAAGATGATAGTGATGAAGAAACTGAAATGAATAATGGAAAATTTACTG

ch4_chro.50317 (181) AGGAAAATGGTAAAGATGATAGTGATGAAGAAACTGAAATGAATAATGGAAAATTTACTG

TU502_chro.50317 (181) AGGAAAATGGTAAAGATGATAGTGATGAAGAAACTGAAATGAATAATGGAAAATTTACTG

rabbit1_chro.50317 (181) AGGAAAATGGTAAAGATGATAGTGATGAAGAAACTGAAATGAATAATGGAAAATTTACTG

rabbit2_chro.50317 (181) AGGAAAATGGTAAAGATGATAGTGATGAAGAAACTGAAATGAATAATGGAAAATTTACTG

rabbit3_chro.50317 (181) AGGAAAATGGTAAAGATGATAGTGATGAAGAAACTGAAATGAATAATGGAAAATTTACTG

rabbit4_chro.50317 (181) AGGAAAATGGTAAAGATGATAGTGATGAAGAAACTGAAATGAATAATGGAAAATTTACTG

cp2_chro.50317 (181) AGGAAAATGGTAAAGATGATAGTGATGAAGAAACTGAAATGAATAATGGAAAATTTACTG

cp3_chro.50317 (181) AGGAAAATGGTAAAGATGATAGTGATGAAGAAACTGAAATGAATAATGGAAAATTTACTG

cp4_chro.50317 (181) AGGAAAATGGTAAAGATGATAGTGATGAAGAAACTGAAATGAATAATGGAAAATTTACTG

Iowa_chro.50317 (181) AGGAAAATGGTAAAGATGATAGTGATGAAGAAACTGAAATGAATAATGGAAAATTTACTG

Moredun_chro.50317 (181) AGGAAAATGGTAAAGATGATAGTGATGAAGAAACTGAAATGAATAATGGAAAATTTACTG

w65_chro.50317 (181) AGGAAAATGGTAAAGATGATAGTGATGAAGAAACTGAAATGAATAATAGAAAATTTACTG

w66_chro.50317 (181) AGGAAAATGGTAAAGATGATAGTGATGAAGAAACTGAAATGAATAATAGAAAATTTACTG

w67_chro.50317 (181) AGGAAAATGGTAAAGATGATAGTGATGAAGAAACTGAAATGAATAATAGAAAATTTACTG

w70_chro.50317 (181) AGGAAAATGGTAAAGATGATAGTGATGAAGAAACTGAAATGAATAATAGAAAATTTACTG

C.meleagridis_chro.50317 (155) AGGAAAATGGTAAAGATGATAGCGATGAAGAAAGTGAAGTTAATAATGGGAAATTTACAG

241 297

ch2_chro.50317 (241) AAGAGGCTAATGAGAATGATGAGTCTGGAAGTGAAGAAGATGGAGATTTAGTTAGGA

ch3_chro.50317 (241) AAGAGGCTAATGAGAATGATGAGTCTGGAAGTGAAGAAGATGGAGATTTAGTTAGGA

ch4_chro.50317 (241) AAGAGGCTAATGAGAATGATGAGTCTGGAAGTGAAGAAGATGGAGATTTAGTTAGGA

TU502_chro.50317 (241) AAGAGGCTAATGAGAATGATGAGTCTGGAAGTGAAGAAGATGGAGATTTAGTTAGGA

rabbit1_chro.50317 (241) AAGAGGCTAATGAGAATGATGAGTCTGGAAGTGAAGAAGATGGAGATTTAGTTAGGA

rabbit2_chro.50317 (241) AAGAGGCTAATGAGAATGATGAGTCTGGAAGTGAAGAAGATGGAGATTTAGTTAGGA

rabbit3_chro.50317 (241) AAGAGGCTAATGAGAATGATGAGTCTGGAAGTGAAGAAGATGGAGATTTAGTTAGGA

rabbit4_chro.50317 (241) AAGAGGCTAATGAGAATGATGAGTCTGGAAGTGAAGAAGATGGAGATTTAGTTAGGA

cp2_chro.50317 (241) AAGAAGCTAATGAGAATGATGAATCTGGAAGTGAAGAAGATGGAGATTTAGTTAGGA

cp3_chro.50317 (241) AAGAAGCTAATGAGAATGATGAATCTGGAAGTGAAGAAGATGGAGATTTAGTTAGGA

cp4_chro.50317 (241) AAGAAGCTAATGAGAATGATGAATCTGGAAGTGAAGAAGATGGAGATTTAGTTAGGA

Iowa_chro.50317 (241) AAGAAGCTAATGAGAATGATGAATCTGGAAGTGAAGAAGATGGAGATTTAGTTAGGA

Moredun_chro.50317 (241) AAGAAGCTAATGAGAATGATGAATCTGGAAGTGAAGAAGATGGAGATTTAGTTAGGA

w65_chro.50317 (241) AAGAAGCTAATGAGAATGATGAATCTGGAAGTGAAGAAGATGGAGATTTAGTTAGGA

w66_chro.50317 (241) AAGAAGCTAATGAGAATGATGAATCTGGAAGTGAAGAAGATGGAGATTTAGTTAGGA

w67_chro.50317 (241) AAGAAGCTAATGAGAATGATGAATCTGGAAGTGAAGAAGATGGAGATTTAGTTAGGA

w70_chro.50317 (241) AAGAAGCTAATGAGAATGATGAATCTGGAAGTGAAGAAGATGGAGATTTAGTTAGGA

C.meleagridis_chro.50317 (215) AAGAGGCTAATGAGAATGATGAGTCTGGAAGTGAAGAAGATGGAGATTTAGTCAGGA

298 360

ch2_chro.50317 (298) GGAGTTTAAATGGAAGGAATAATTTTACTGGTGAAAATCTAACGGAGAATGATTCAAAAA

ch3_chro.50317 (298) GGAGTTTAAATGGAAGGAATAATTTTACTGGTGAAAATCTAACGGAGAATGATTCAAAAA

ch4_chro.50317 (298) GGAGTTTAAATGGAAGGAATAATTTTACTGGTGAAAATCTAACGGAGAATGATTCAAAAA

TU502_chro.50317 (298) GGAGTTTAAATGGAAGGAATAATTTTACTGGTGAAAATCTAACGGAGAATGATTCAAAAA

rabbit1_chro.50317 (298) GGAGTTTAAATGCAAGGAATAATTTTACTGGTGAAAATCTAACGGAGAATGATTCAAAAA

rabbit2_chro.50317 (298) GGAGTTTAAATGCAAGGAATAATTTTACTGGTGAAAATCTAACGGAGAATGATTCAAAAA

rabbit3_chro.50317 (298) GGAGTTTAAATGCAAGGAATAATTTTACTGGTGAAAATCTAACGGAGAATGATTCAAAAA

rabbit4_chro.50317 (298) GGAGTTTAAATGCAAGGAATAATTTTACTGGTGAAAATCTAACGGAGAATGATTCAAAAA

cp2_chro.50317 (298) GGAGTTTAAGTGCAAGGAATAGTTTTACTGGTGAAAATCTAATGGAGAATGATTCAAAAA

cp3_chro.50317 (298) GGAGTTTAAGTGCAAGGAATAGTTTTACTGGTGAAAATCTAATGGAGAATGATTCAAAAA

cp4_chro.50317 (298) GGAGTTTAAGTGCAAGGAATAGTTTTACTGGTGAAAATCTAATGGAGAATGATTCAAAAA

Iowa_chro.50317 (298) GGAGTTTAAGTGCAAGGAATAGTTTTACTGGTGAAAATCTAATGGAGAATGATTCAAAAA

Moredun_chro.50317 (298) GGAGTTTAAGTGCAAGGAATAGTTTTACTGGTGAAAATCTAATGGAGAATGATTCAAAAA

w65_chro.50317 (298) GGAGTTTAAATGCAAGGAATAGTTTTACTGGTGAAAATCTAATGGAGAATGATTCAAAAA

w66_chro.50317 (298) GGAGTTTAAATGCAAGGAATAGTTTTACTGGTGAAAATCTAATGGAGAATGATTCAAAAA

w67_chro.50317 (298) GGAGTTTAAATGCAAGGAATAGTTTTACTGGTGAAAATCTAATGGAGAATGATTCAAAAA

w70_chro.50317 (298) GGAGTTTAAATGCAAGGAATAGTTTTACTGGTGAAAATCTAATGGAGAATGATTCAAAAA

C.meleagridis_chro.50317 (275) AAAGTTTAAATGCAAGGAATAGTTTTGCTGGTGGAAATCTAATGGAGAATGATTCAAAAA

361 420

ch2_chro.50317 (358) TACCATGCATAAACTTAAAAATCTTGACTAAAGATGTATTAAAACATGTCATTGATTTTA

ch3_chro.50317 (358) TACCATGCATAAACTTAAAAATCTTGACTAAAGATGTATTAAAACATGTCATTGATTTTA

ch4_chro.50317 (358) TACCATGCATAAACTTAAAAATCTTGACTAAAGATGTATTAAAACATGTCATTGATTTTA

TU502_chro.50317 (358) TACCATGCATAAACTTAAAAATCTTGACTAAAGATGTATTAAAACATGTCATTGATTTTA

rabbit1_chro.50317 (358) TACCATGCATAAACTTAAAAATCTTGACTAAAGATGTATTAAAACATGTCATTGATTTTA

rabbit2_chro.50317 (358) TACCATGCATAAACTTAAAAATCTTGACTAAAGATGTATTAAAACATGTCATTGATTTTA

rabbit3_chro.50317 (358) TACCATGCATAAACTTAAAAATCTTGACTAAAGATGTATTAAAACATGTCATTGATTTTA

rabbit4_chro.50317 (358) TACCATGCATAAACTTAAAAATCTTGACTAAAGATGTATTAAAACATGTCATTGATTTTA

cp2_chro.50317 (358) TACCATGCATAAACTTAAAAATCTTGACTAAAGATGTATTAAAACATGTCATTGATTTTA

cp3_chro.50317 (358) TACCATGCATAAACTTAAAAATCTTGACTAAAGATGTATTAAAACATGTCATTGATTTTA

cp4_chro.50317 (358) TACCATGCATAAACTTAAAAATCTTGACTAAAGATGTATTAAAACATGTCATTGATTTTA

Iowa_chro.50317 (358) TACCATGCATAAACTTAAAAATCTTGACTAAAGATGTATTAAAACATGTCATTGATTTTA

Moredun_chro.50317 (358) TACCATGCATAAACTTAAAAATCTTGACTAAAGATGTATTAAAACATGTCATTGATTTTA

w65_chro.50317 (358) TACCATGCATAAACTTAAAAATCTTGACTAAAGATGTATTAAAACATGTCATTGATTTTA

w66_chro.50317 (358) TACCATGCATAAACTTAAAAATCTTGACTAAAGATGTATTAAAACATGTCATTGATTTTA

w67_chro.50317 (358) TACCATGCATAAACTTAAAAATCTTGACTAAAGATGTATTAAAACATGTCATTGATTTTA

w70_chro.50317 (358) TACCATGCATAAACTTAAAAATCTTGACTAAAGATGTATTAAAACATGTCATTGATTTTA

C.meleagridis_chro.50317 (335) TACCATCCATAAACTTAAAAATCTTGACTAAAGATGTACTAAAACATGTCATTGACTTTA

421 480

ch2_chro.50317 (418) ACATTGGTAGAAAAACCAATATTTTGAGTATTAAATTGGGGTGGCCAGTTATAAGATGCC

ch3_chro.50317 (418) ACATTGGTAGAAAAACCAATATTTTGAGTATTAAATTGGGGTGGCCAGTTATAAGATGCC

ch4_chro.50317 (418) ACATTGGTAGAAAAACCAATATTTTGAGTATTAAATTGGGGTGGCCAGTTATAAGATGCC

TU502_chro.50317 (418) ACATTGGTAGAAAAACCAATATTTTGAGTATTAAATTGGGGTGGCCAGTTATAAGATGCC

rabbit1_chro.50317 (418) ACATTGATAGAAAAACCAATATTTTGAGTATTAAATTGGGGTGGCCAGTTATAAGATGCC

rabbit2_chro.50317 (418) ACATTGATAGAAAAACCAATATTTTGAGTATTAAATTGGGGTGGCCAGTTATAAGATGCC

rabbit3_chro.50317 (418) ACATTGATAGAAAAACCAATATTTTGAGTATTAAATTGGGGTGGCCAGTTATAAGATGCC

rabbit4_chro.50317 (418) ACATTGATAGAAAAACCAATATTTTGAGTATTAAATTGGGGTGGCCAGTTATAAGATGCC

cp2_chro.50317 (418) ACATTGATAGAAAAACCAATATTTTGAGTATTAAATTGGGGTGGCCGGTTATAAGATGCC

cp3_chro.50317 (418) ACATTGATAGAAAAACCAATATTTTGAGTATTAAATTGGGGTGGCCGGTTATAAGATGCC

cp4_chro.50317 (418) ACATTGATAGAAAAACCAATATTTTGAGTATTAAATTGGGGTGGCCGGTTATAAGATGCC

Iowa_chro.50317 (418) ACATTGATAGAAAAACCAATATTTTGAGTATTAAATTGGGGTGGCCGGTTATAAGATGCC

Moredun_chro.50317 (418) ACATTGATAGAAAAACCAATATTTTGAGTATTAAATTGGGGTGGCCGGTTATAAGATGCC

w65_chro.50317 (418) ACATTGATAGAAAAACCAATATTTTGAGTATTAAATTGGGGTGGCCGGTTATAAGATGCC

w66_chro.50317 (418) ACATTGATAGAAAAACCAATATTTTGAGTATTAAATTGGGGTGGCCGGTTATAAGATGCC

w67_chro.50317 (418) ACATTGATAGAAAAACCAATATTTTGAGTATTAAATTGGGGTGGCCGGTTATAAGATGCC

w70_chro.50317 (418) ACATTGATAGAAAAACCAATATTTTGAGTATTAAATTGGGGTGGCCGGTTATAAGATGCC

C.meleagridis_chro.50317 (395) ACATTGATAGAAAGACCAATATTTTGAGTATTAAATTGGGATGGCCGGTTATAAGATGTC

481 540

ch2_chro.50317 (478) CACATTATATTGATTTCTTACCTGTCTTAAAAAATTGTATTTTAAGGACAAATATCCAGT

ch3_chro.50317 (478) CACATTATATTGATTTCTTACCTGTCTTAAAAAATTGTATTTTAAGGACAAATATCCAGT

ch4_chro.50317 (478) CACATTATATTGATTTCTTACCTGTCTTAAAAAATTGTATTTTAAGGACAAATATCCAGT

TU502_chro.50317 (478) CACATTATATTGATTTCTTACCTGTCTTAAAAAATTGTATTTTAAGGACAAATATCCAGT

rabbit1_chro.50317 (478) CACATTATATTGATTTCTTACCTGTCTTAAAAAATTGTATTTTAAGGACAAATATCCAGT

rabbit2_chro.50317 (478) CACATTATATTGATTTCTTACCTGTCTTAAAAAATTGTATTTTAAGGACAAATATCCAGT

rabbit3_chro.50317 (478) CACATTATATTGATTTCTTACCTGTCTTAAAAAATTGTATTTTAAGGACAAATATCCAGT

rabbit4_chro.50317 (478) CACATTATATTGATTTCTTACCTGTCTTAAAAAATTGTATTTTAAGGACAAATATCCAGT

cp2_chro.50317 (478) CACATTATATTGATTTCTTACCTGTCTTAAAAAATTGTATTTTAAAGACAAATATCCAGT

cp3_chro.50317 (478) CACATTATATTGATTTCTTACCTGTCTTAAAAAATTGTATTTTAAAGACAAATATCCAGT

cp4_chro.50317 (478) CACATTATATTGATTTCTTACCTGTCTTAAAAAATTGTATTTTAAAGACAAATATCCAGT

Iowa_chro.50317 (478) CACATTATATTGATTTCTTACCTGTCTTAAAAAATTGTATTTTAAAGACAAATATCCAGT

Moredun_chro.50317 (478) CACATTATATTGATTTCTTACCTGTCTTAAAAAATTGTATTTTAAAGACAAATATCCAGT

w65_chro.50317 (478) CACATTATATTGATTTCTTACCTGTCTTAAAAAATTGTATTTTAAAGACAAATATTCAGT

w66_chro.50317 (478) CACATTATATTGATTTCTTACCTGTCTTAAAAAATTGTATTTTAAAGACAAATATTCAGT

w67_chro.50317 (478) CACATTATATTGATTTCTTACCTGTCTTAAAAAATTGTATTTTAAAGACAAATATTCAGT

w70_chro.50317 (478) CACATTATATTGATTTCTTACCTGTCTTAAAAAATTGTATTTTAAAGACAAATATTCAGT

C.meleagridis_chro.50317 (455) CACATTATATTGATTTTTTACCTGTCTTAAAAAATTGTATTTTAAAAACAAATATCCATT

541 600

ch2_chro.50317 (538) CAATTTCATGTTTGAAAAATGCAAGAATTACTAGGAACCAGAAAGAAACTTCAGACAAAA

ch3_chro.50317 (538) CAATTTCATGTTTGAAAAATGCAAGAATTACTAGGAACCAGAAAGAAACTTCAGACAAAA

ch4_chro.50317 (538) CAATTTCATGTTTGAAAAATGCAAGAATTACTAGGAACCAGAAAGAAACTTCAGACAAAA

TU502_chro.50317 (538) CAATTTCATGTTTGAAAAATGCAAGAATTACTAGGAACCAGAAAGAAACTTCAGACAAAA

rabbit1_chro.50317 (538) CAATTTCATGTTTGAAAAATGCAAGAATTACTAGGAACCAGAAAGAAACTTCAGACAAAA

rabbit2_chro.50317 (538) CAATTTCATGTTTGAAAAATGCAAGAATTACTAGGAACCAGAAAGAAACTTCAGACAAAA

rabbit3_chro.50317 (538) CAATTTCATGTTTGAAAAATGCAAGAATTACTAGGAACCAGAAAGAAACTTCAGACAAAA

rabbit4_chro.50317 (538) CAATTTCATGTTTGAAAAATGCAAGAATTACTAGGAACCAGAAAGAAACTTCAGACAAAA

cp2_chro.50317 (538) CAATTTCATGTTTGAAAAATGCAAGAATTACTAGGAACCAGAAAGAAACTTCAGACAAAA

cp3_chro.50317 (538) CAATTTCATGTTTGAAAAATGCAAGAATTACTAGGAACCAGAAAGAAACTTCAGACAAAA

cp4_chro.50317 (538) CAATTTCATGTTTGAAAAATGCAAGAATTACTAGGAACCAGAAAGAAACTTCAGACAAAA

Iowa_chro.50317 (538) CAATTTCATGTTTGAAAAATGCAAGAATTACTAGGAACCAGAAAGAAACTTCAGACAAAA

Moredun_chro.50317 (538) CAATTTCATGTTTGAAAAATGCAAGAATTACTAGGAACCAGAAAGAAACTTCAGACAAAA

w65_chro.50317 (538) CAATTTCATGTTTGAAAAATGCAAGAATTACTAGGAACCAGAAAGAAACTTCAGACAAAA

w66_chro.50317 (538) CAATTTCATGTTTGAAAAATGCAAGAATTACTAGGAACCAGAAAGAAACTTCAGACAAAA

w67_chro.50317 (538) CAATTTCATGTTTGAAAAATGCAAGAATTACTAGGAACCAGAAAGAAACTTCAGACAAAA

w70_chro.50317 (538) CAATTTCATGTTTGAAAAATGCAAGAATTACTAGGAACCAGAAAGAAACTTCAGACAAAA

C.meleagridis_chro.50317 (515) CAATTTCATGTTTGAAAAATGCAAGAATTACCAGGAACCAGAAGGAGACTTCAGACAAGA

601 660

ch2_chro.50317 (598) GTTTGTCTCAGTTTGAAGTTACTGTGGAGGGAACCAATGTAGGGCATGTTTTTAAGATTT

ch3_chro.50317 (598) GTTTGTCTCAGTTTGAAGTTACTGTGGAGGGAACCAATGTAGGGCATGTTTTTAAGATTT

ch4_chro.50317 (598) GTTTGTCTCAGTTTGAAGTTACTGTGGAGGGAACCAATGTAGGGCATGTTTTTAAGATTT

TU502_chro.50317 (598) GTTTGTCTCAGTTTGAAGTTACTGTGGAGGGAACCAATGTAGGGCATGTTTTTAAGATTT

rabbit1_chro.50317 (598) GTTTGTCTCAGTTTGAAGTTACTGTGGAGGGAACCAATGTAGGGCATGTTTTTAAGATTT

rabbit2_chro.50317 (598) GTTTGTCTCAGTTTGAAGTTACTGTGGAGGGAACCAATGTAGGGCATGTTTTTAAGATTT

rabbit3_chro.50317 (598) GTTTGTCTCAGTTTGAAGTTACTGTGGAGGGAACCAATGTAGGGCATGTTTTTAAGATTT

rabbit4_chro.50317 (598) GTTTGTCTCAGTTTGAAGTTACTGTGGAGGGAACCAATGTAGGGCATGTTTTTAAGATTT

cp2_chro.50317 (598) GTTTGTCTCAGTTTGAAGTTACCGTGGAGGGAACCAATGTAGGGCATATTTTTAAGATTT

cp3_chro.50317 (598) GTTTGTCTCAGTTTGAAGTTACCGTGGAGGGAACCAATGTAGGGCATATTTTTAAGATTT

cp4_chro.50317 (598) GTTTGTCTCAGTTTGAAGTTACCGTGGAGGGAACCAATGTAGGGCATATTTTTAAGATTT

Iowa_chro.50317 (598) GTTTGTCTCAGTTTGAAGTTACCGTGGAGGGAACCAATGTAGGGCATATTTTTAAGATTT

Moredun_chro.50317 (598) GTTTGTCTCAGTTTGAAGTTACCGTGGAGGGAACCAATGTAGGGCATATTTTTAAGATTT

w65_chro.50317 (598) GTTTGTCTCAGTTTGAAGTTACCGTGGAGGGAACCAATGTAGGGCATATTTTTAAGATTT

w66_chro.50317 (598) GTTTGTCTCAGTTTGAAGTTACCGTGGAGGGAACCAATGTAGGGCATATTTTTAAGATTT

w67_chro.50317 (598) GTTTGTCTCAGTTTGAAGTTACCGTGGAGGGAACCAATGTAGGGCATATTTTTAAGATTT

w70_chro.50317 (598) GTTTGTCTCAGTTTGAAGTTACCGTGGAGGGAACCAATGTAGGGCATATTTTTAAGATTT

C.meleagridis_chro.50317 (575) GTTTGTCTCAGTTTGAAGTTACTGTGGAGGGAACTAATGTGGGGCATATTTTTAAGATTT

661 720

ch2_chro.50317 (658) CGCCACGTTACATTAATCACGATAAAATTAGATTCAATGATATTCAAACAGTATATAAAT

ch3_chro.50317 (658) CGCCACGTTACATTAATCACGATAAAATTAGATTCAATGATATTCAAACAGTATATAAAT

ch4_chro.50317 (658) CGCCACGTTACATTAATCACGATAAAATTAGATTCAATGATATTCAAACAGTATATAAAT

TU502_chro.50317 (658) CGCCACGTTACATTAATCACGATAAAATTAGATTCAATGATATTCAAACAGTATATAAAT

rabbit1_chro.50317 (658) CGCCACGTTACATTAATCACGATAAAATTAGATTCAATGATATTCAAACAGTATATAAAT

rabbit2_chro.50317 (658) CGCCACGTTACATTAATCACGATAAAATTAGATTCAATGATATTCAAACAGTATATAAAT

rabbit3_chro.50317 (658) CGCCACGTTACATTAATCACGATAAAATTAGATTCAATGATATTCAAACAGTATATAAAT

rabbit4_chro.50317 (658) CGCCACGTTACATTAATCACGATAAAATTAGATTCAATGATATTCAAACAGTATATAAAT

cp2_chro.50317 (658) CGCCACGTTACATTAATCACGATAAAATTAGATTCAATGATATTCAAACAGTATATAAAT

cp3_chro.50317 (658) CGCCACGTTACATTAATCACGATAAAATTAGATTCAATGATATTCAAACAGTATATAAAT

cp4_chro.50317 (658) CGCCACGTTACATTAATCACGATAAAATTAGATTCAATGATATTCAAACAGTATATAAAT

Iowa_chro.50317 (658) CGCCACGTTACATTAATCACGATAAAATTAGATTCAATGATATTCAAACAGTATATAAAT

Moredun_chro.50317 (658) CGCCACGTTACATTAATCACGATAAAATTAGATTCAATGATATTCAAACAGTATATAAAT

w65_chro.50317 (658) CGCCACGTTACATTAATCACGATAAAATTAGATTCAATGATATTCAAACAGTATATAAAT

w66_chro.50317 (658) CGCCACGTTACATTAATCACGATAAAATTAGATTCAATGATATTCAAACAGTATATAAAT

w67_chro.50317 (658) CGCCACGTTACATTAATCACGATAAAATTAGATTCAATGATATTCAAACAGTATATAAAT

w70_chro.50317 (658) CGCCACGTTACATTAATCACGATAAAATTAGATTCAATGATATTCAAACAGTATATAAAT

C.meleagridis_chro.50317 (635) CGCCACGTTACATTAATCACGATAAAATTAGATTCAATGTT----------------

721

ch2_chro.50317 (718) ATTATGGTGT

ch3_chro.50317 (718) ATTATGGTGT

ch4_chro.50317 (718) ATTATGGTGT

TU502_chro.50317 (718) ATTATGGTGT

rabbit1_chro.50317 (718) ATTATGGTGT

rabbit2_chro.50317 (718) ATTATGGTGT

rabbit3_chro.50317 (718) ATTATGGTGT

rabbit4_chro.50317 (718) ATTATGGTGT

cp2_chro.50317 (718) ATTATGGTGT

cp3_chro.50317 (718) ATTATGGTGT

cp4_chro.50317 (718) ATTATGGTGT

Iowa_chro.50317 (718) ATTATGGTGT

Moredun_chro.50317 (718) ATTATGGTGT

w65_chro.50317 (718) ATTATGGTGT

w66_chro.50317 (718) ATTATGGTGT

w67_chro.50317 (718) ATTATGGTGT

w70_chro.50317 (718) ATTATGGTGT

C.meleagridis_chro.50317 (678) ----------

**Chro.50330 gene PCR products**

1 60

ch2_chro.50330 (1) CAGGTTCAGAAACTCTGGGAAGAGAGTCAAATATATGAGGCAGATGTAGATCCATCTCGT

ch3_chro.50330 (1) CAGGTTCAGAAACTCTGGGAAGAGAGTCAAATATATGAGGCAGATGTAGATCCATCTCGT

ch4_chro.50330 (1) CAGGTTCAGAAACTCTGGGAAGAGAGTCAAATATATGAGGCAGATGTAGATCCATCTCGT

TU502_chro.50330 (1) CAGGTTCAGAAACTCTGGGAAGAGAGTCAAATATATGAGGCAGATGTAGATCCATCTCGT

cp2_chro.50330 (1) CAGGTTCAAAAACTCTGGGAAGAGAGTCAAATATATGAGGCAGATGTAGATCCATCTCGT

cp3_chro.50330 (1) CAGGTTCAAAAACTCTGGGAAGAGAGTCAAATATATGAGGCAGATGTAGATCCATCTCGT

cp4_chro.50330 (1) CAGGTTCAAAAACTCTGGGAAGAGAGTCAAATATATGAGGCAGATGTAGATCCATCTCGT

Iowa_chro.50330 (1) CAGGTTCAAAAACTCTGGGAAGAGAGTCAAATATATGAGGCAGATGTAGATCCATCTCGT

Moredun_chro.50330 (1) CAGGTTCAAAAACTCTGGGAAGAGAGTCAAATATATGAGGCAGATGTAGATCCATCTCGT

w65_chro.50330 (1) CAGGTTCAAAAACTCTGGGAAGAGAGTCAAATATATGAGGCAGATGTAGATCCATCTCGT

w66_chro.50330 (1) CAGGTTCAAAAACTCTGGGAAGAGAGTCAAATATATGAGGCAGATGTAGATCCATCTCGT

w67_chro.50330 (1) CAGGTTCAAAAACTCTGGGAAGAGAGTCAAATATATGAGGCAGATGTAGATCCATCTCGT

w70_chro.50330 (1) CAGGTTCAAAAACTCTGGGAAGAGAGTCAAATATATGAGGCAGATGTAGATCCATCTCGT

rabbit1_chro.50330 (1) CAGGTTCAGAAACTCTGGGAAGAGAGTCAAATATATGAGGCAGATGTAGATCCATCTCGT

rabbit2_chro.50330 (1) CAGGTTCAGAAACTCTGGGAAGAGAGTCAAATATATGAGGCAGATGTAGATCCATCTCGT

rabbit3_chro.50330 (1) CAGGTTCAGAAACTCTGGGAAGAGAGTCAAATATATGAGGCAGATGTAGATCCATCTCGT

rabbit4_chro.50330 (1) CAGGTTCAGAAACTCTGGGAAGAGAGTCAAATATATGAGGCAGATGTAGATCCATCTCGT

61 120

ch2_chro.50330 (61) GAAAAATATATGATTACATTTCCATATCCATATATGAATGGAAGGTTACATTTGGGGCAT

ch3_chro.50330 (61) GAAAAATATATGATTACATTTCCATATCCATATATGAATGGAAGGTTACATTTGGGGCAT

ch4_chro.50330 (61) GAAAAATATATGATTACATTTCCATATCCATATATGAATGGAAGGTTACATTTGGGGCAT

TU502_chro.50330 (61) GAAAAATATATGATTACATTTCCATATCCATATATGAATGGAAGGTTACATTTGGGGCAT

cp2_chro.50330 (61) GAAAAATACATGATTACATTTCCATATCCATATATGAATGGAAGGTTACATTTGGGGCAT

cp3_chro.50330 (61) GAAAAATACATGATTACATTTCCATATCCATATATGAATGGAAGGTTACATTTGGGGCAT

cp4_chro.50330 (61) GAAAAATACATGATTACATTTCCATATCCATATATGAATGGAAGGTTACATTTGGGGCAT

Iowa_chro.50330 (61) GAAAAATACATGATTACATTTCCATATCCATATATGAATGGAAGGTTACATTTGGGGCAT

Moredun_chro.50330 (61) GAAAAATACATGATTACATTTCCATATCCATATATGAATGGAAGGTTACATTTGGGGCAT

w65_chro.50330 (61) GAAAAATACATGATTACATTTCCATATCCATATATGAATGGAAGGTTACATTTGGGGCAT

w66_chro.50330 (61) GAAAAATACATGATTACATTTCCATATCCATATATGAATGGAAGGTTACATTTGGGGCAT

w67_chro.50330 (61) GAAAAATACATGATTACATTTCCATATCCATATATGAATGGAAGGTTACATTTGGGGCAT

w70_chro.50330 (61) GAAAAATACATGATTACATTTCCATATCCATATATGAATGGAAGGTTACATTTGGGGCAT

rabbit1_chro.50330 (61) GAAAAATATATGATTACATTTCCATATCCATATATGAATGGAAGGTTACATTTGGGGCAT

rabbit2_chro.50330 (61) GAAAAATATATGATTACATTTCCATATCCATATATGAATGGAAGGTTACATTTGGGGCAT

rabbit3_chro.50330 (61) GAAAAATATATGATTACATTTCCATATCCATATATGAATGGAAGGTTACATTTGGGGCAT

rabbit4_chro.50330 (61) GAAAAATATATGATTACATTTCCATATCCATATATGAATGGAAGGTTACATTTGGGGCAT

121 180

ch2_chro.50330 (121) GCCTTTACGTTGACAAAAGCAGATTTTCAAGCAAGATTCCAAAGAATGAATAATAAAAAT

ch3_chro.50330 (121) GCCTTTACGTTGACAAAAGCAGATTTTCAAGCAAGATTCCAAAGAATGAATAATAAAAAT

ch4_chro.50330 (121) GCCTTTACGTTGACAAAAGCAGATTTTCAAGCAAGATTCCAAAGAATGAATAATAAAAAT

TU502_chro.50330 (121) GCCTTTACGTTGACAAAAGCAGATTTTCAAGCAAGATTCCAAAGAATGAATAATAAAAAT

cp2_chro.50330 (121) GCCTTTACGTTGACAAAAGCAGATTTTCAAGCAAGATTCCAAAGAATGAATAATAAAAAT

cp3_chro.50330 (121) GCCTTTACGTTGACAAAAGCAGATTTTCAAGCAAGATTCCAAAGAATGAATAATAAAAAT

cp4_chro.50330 (121) GCCTTTACGTTGACAAAAGCAGATTTTCAAGCAAGATTCCAAAGAATGAATAATAAAAAT

Iowa_chro.50330 (121) GCCTTTACGTTGACAAAAGCAGATTTTCAAGCAAGATTCCAAAGAATGAATAATAAAAAT

Moredun_chro.50330 (121) GCCTTTACGTTGACAAAAGCAGATTTTCAAGCAAGATTCCAAAGAATGAATAATAAAAAT

w65_chro.50330 (121) GCCTTTACGTTGACAAAAGCAGATTTTCAAGCAAGATTCCAAAGAATGAATAATAAAAAT

w66_chro.50330 (121) GCCTTTACGTTGACAAAAGCAGATTTTCAAGCAAGATTCCAAAGAATGAATAATAAAAAT

w67_chro.50330 (121) GCCTTTACGTTGACAAAAGCAGATTTTCAAGCAAGATTCCAAAGAATGAATAATAAAAAT

w70_chro.50330 (121) GCCTTTACGTTGACAAAAGCAGATTTTCAAGCAAGATTCCAAAGAATGAATAATAAAAAT

rabbit1_chro.50330 (121) GCCTTTACGTTGACAAAAGCAGATTTTCAAGCAAGATTCCAAAGAATGAATAATAAAAAT

rabbit2_chro.50330 (121) GCCTTTACGTTGACAAAAGCAGATTTTCAAGCAAGATTCCAAAGAATGAATAATAAAAAT

rabbit3_chro.50330 (121) GCCTTTACGTTGACAAAAGCAGATTTTCAAGCAAGATTCCAAAGAATGAATAATAAAAAT

rabbit4_chro.50330 (121) GCCTTTACGTTGACAAAAGCAGATTTTCAAGCAAGATTCCAAAGAATGAATAATAAAAAT

181 240

ch2_chro.50330 (181) GTATTATTTCCATTTGGTTTTCATTGTACAGGAATGCCAATTTGTGCAAGCGCAGATAAG

ch3_chro.50330 (181) GTATTATTTCCATTTGGTTTTCATTGTACAGGAATGCCAATTTGTGCAAGCGCAGATAAG

ch4_chro.50330 (181) GTATTATTTCCATTTGGTTTTCATTGTACAGGAATGCCAATTTGTGCAAGCGCAGATAAG

TU502_chro.50330 (181) GTATTATTTCCATTTGGTTTTCATTGTACAGGAATGCCAATTTGTGCAAGCGCAGATAAG

cp2_chro.50330 (181) GTATTATTTCCATTTGGTTTTCATTGTACGGGAATGCCAATTTGTGCAAGCGCAGATAAG

cp3_chro.50330 (181) GTATTATTTCCATTTGGTTTTCATTGTACGGGAATGCCAATTTGTGCAAGCGCAGATAAG

cp4_chro.50330 (181) GTATTATTTCCATTTGGTTTTCATTGTACGGGAATGCCAATTTGTGCAAGCGCAGATAAG

Iowa_chro.50330 (181) GTATTATTTCCATTTGGTTTTCATTGTACGGGAATGCCAATTTGTGCAAGCGCAGATAAG

Moredun_chro.50330 (181) GTATTATTTCCATTTGGTTTTCATTGTACGGGAATGCCAATTTGTGCAAGCGCAGATAAG

w65_chro.50330 (181) GTATTATTTCCATTTGGTTTTCATTGTACGGGAATGCCAATTTGTGCAAGCGCAGATAAG

w66_chro.50330 (181) GTATTATTTCCATTTGGTTTTCATTGTACGGGAATGCCAATTTGTGCAAGCGCAGATAAG

w67_chro.50330 (181) GTATTATTTCCATTTGGTTTTCATTGTACGGGAATGCCAATTTGTGCAAGCGCAGATAAG

w70_chro.50330 (181) GTATTATTTCCATTTGGTTTTCATTGTACGGGAATGCCAATTTGTGCAAGCGCAGATAAG

rabbit1_chro.50330 (181) GTATTATTTCCATTTGGTTTTCATTGTACAGGAATGCCAATTTGTGCAAGCGCAGATAAG

rabbit2_chro.50330 (181) GTATTATTTCCATTTGGTTTTCATTGTACAGGAATGCCAATTTGTGCAAGCGCAGATAAG

rabbit3_chro.50330 (181) GTATTATTTCCATTTGGTTTTCATTGTACAGGAATGCCAATTTGTGCAAGCGCAGATAAG

rabbit4_chro.50330 (181) GTATTATTTCCATTTGGTTTTCATTGTACAGGAATGCCAATTTGTGCAAGCGCAGATAAG

241 300

ch2_chro.50330 (241) TTAAAGATGGAGTTGAATTCACCAAAGTTAGCTGAAACTGATGATGATAAAGAACAAGAG

ch3_chro.50330 (241) TTAAAGATGGAGTTGAATTCACCAAAGTTAGCTGAAACTGATGATGATAAAGAACAAGAG

ch4_chro.50330 (241) TTAAAGATGGAGTTGAATTCACCAAAGTTAGCTGAAACTGATGATGATAAAGAACAAGAG

TU502_chro.50330 (241) TTAAAGATGGAGTTGAATTCACCAAAGTTAGCTGAAACTGATGATGATAAAGAACAAGAG

cp2_chro.50330 (241) TTAAAGATGGAGTTGAATTCACCAAAGTTAGCTGAAACTGATGATGATAAAGAACAAGAG

cp3_chro.50330 (241) TTAAAGATGGAGTTGAATTCACCAAAGTTAGCTGAAACTGATGATGATAAAGAACAAGAG

cp4_chro.50330 (241) TTAAAGATGGAGTTGAATTCACCAAAGTTAGCTGAAACTGATGATGATAAAGAACAAGAG

Iowa_chro.50330 (241) TTAAAGATGGAGTTGAATTCACCAAAGTTAGCTGAAACTGATGATGATAAAGAACAAGAG

Moredun_chro.50330 (241) TTAAAGATGGAGTTGAATTCACCAAAGTTAGCTGAAACTGATGATGATAAAGAACAAGAG

w65_chro.50330 (241) TTAAAGATGGAGTTGAATTCACCAAAGTTAGCTGAAACTGATGATGATAAAGAACAAGAG

w66_chro.50330 (241) TTAAAGATGGAGTTGAATTCACCAAAGTTAGCTGAAACTGATGATGATAAAGAACAAGAG

w67_chro.50330 (241) TTAAAGATGGAGTTGAATTCACCAAAGTTAGCTGAAACTGATGATGATAAAGAACAAGAG

w70_chro.50330 (241) TTAAAGATGGAGTTGAATTCACCAAAGTTAGCTGAAACTGATGATGATAAAGAACAAGAG

rabbit1_chro.50330 (241) TTAAAGATGGAGTTGAATTCACCAAAGTTAGCTGAAACTGATGATGATAAAGAACAAGAG

rabbit2_chro.50330 (241) TTAAAGATGGAGTTGAATTCACCAAAGTTAGCTGAAACTGATGATGATAAAGAACAAGAG

rabbit3_chro.50330 (241) TTAAAGATGGAGTTGAATTCACCAAAGTTAGCTGAAACTGATGATGATAAAGAACAAGAG

rabbit4_chro.50330 (241) TTAAAGATGGAGTTGAATTCACCAAAGTTAGCTGAAACTGATGATGATAAAGAACAAGAG

301 346

ch2_chro.50330 (301) ACAAGCCAAGTACAACTTAAAAGTAAGGTTGCAGCTAAAACTGGGG

ch3_chro.50330 (301) ACAAGCCAAGTACAACTTAAAAGTAAGGTTGCAGCTAAAACTGGGG

ch4_chro.50330 (301) ACAAGCCAAGTACAACTTAAAAGTAAGGTTGCAGCTAAAACTGGGG

TU502_chro.50330 (301) ACAAGCCAAGTACAACTTAAAAGTAAGGTTGCAGCTAAAACTGGGG

cp2_chro.50330 (301) ACAAGCCAAGTACAACTTAAAAGTAAGGTTGCAGCTAAAACTGGGG

cp3_chro.50330 (301) ACAAGCCAAGTACAACTTAAAAGTAAGGTTGCAGCTAAAACTGGGG

cp4_chro.50330 (301) ACAAGCCAAGTACAACTTAAAAGTAAGGTTGCAGCTAAAACTGGGG

Iowa_chro.50330 (301) ACAAGCCAAGTACAACTTAAAAGTAAGGTTGCAGCTAAAACTGGGG

Moredun_chro.50330 (301) ACAAGCCAAGTACAACTTAAAAGTAAGGTTGCAGCTAAAACTGGGG

w65_chro.50330 (301) ACAAGCCAAGTACAACTTAAAAGTAAGGTTGCAGCTAAAACTGGGG

w66_chro.50330 (301) ACAAGCCAAGTACAACTTAAAAGTAAGGTTGCAGCTAAAACTGGGG

w67_chro.50330 (301) ACAAGCCAAGTACAACTTAAAAGTAAGGTTGCAGCTAAAACTGGGG

w70_chro.50330 (301) ACAAGCCAAGTACAACTTAAAAGTAAGGTTGCAGCTAAAACTGGGG

rabbit1_chro.50330 (301) ACAAGCCAAGTACAACTTAAAAGTAAGGTTGCAGCTAAAACTGGGG

rabbit2_chro.50330 (301) ACAAGCCAAGTACAACTTAAAAGTAAGGTTGCAGCTAAAACTGGGG

rabbit3_chro.50330 (301) ACAAGCCAAGTACAACTTAAAAGTAAGGTTGCAGCTAAAACTGGGG

rabbit4_chro.50330 (301) ACAAGCCAAGTACAACTTAAAAGTAAGGTTGCAGCTAAAACTGGGG

**Chro.50457 gene PCR products**

1 60

ch2_chro.50457 (1) TCCCGAATATAGTATTAATAATGATAAACTCGAGCTTGATAAATTAAAAGAAAATTATTC

ch3_chro.50457 (1) TCCCGAATATAGTATTAATAATGATAAACTCGAGCTTGATAAATTAAAAGAAAATTATTC

ch4_chro.50457 (1) TCCCGAATATAGTATTAATAATGATAAACTCGAGCTTGATAAATTAAAAGAAAATTATTC

TU502_chro.50457 (1) TCCCGAATATAGTATTAATAATGATAAACTCGAGCTTGATAAATTAAAAGAAAATTATTC

cp2_chro.50457 (1) TCCCGAATATAGTATTAATAATGATAAACTCGAGCTTGATAAATCAAAGGAAAATTATTC

cp3_chro.50457 (1) TCCCGAATATAGTATTAATAATGATAAACTCGAGCTTGATAAATCAAAGGAAAATTATTC

cp4_chro.50457 (1) TCCCGAATATAGTATTAATAATGATAAACTCGAGCTTGATAAATCAAAGGAAAATTATTC

Iowa_chro.50457 (1) TCCCGAATATAGTATTAATAATGATAAACTCGAGCTTGATAAATCAAAGGAAAATTATTC

Moredun_chro.50457 (1) TCCCGAATATAGTATTAATAATGATAAACTCGAGCTTGATAAATCAAAGGAAAATTATTC

w65_chro.50457 (1) TCCCGAATATAGTATTAATAATGATAAACTCGAGCTTGATAAATCAAAGGAAAATTATTC

w66_chro.50457 (1) TCCCGAATATAGTATTAATAATGATAAACTCGAGCTTGATAAATCAAAGGAAAATTATTC

w67_chro.50457 (1) TCCCGAATATAGTATTAATAATGATAAACTCGAGCTTGATAAATCAAAGGAAAATTATTC

w70_chro.50457 (1) TCCCGAATATAGTATTAATAATGATAAACTCGAGCTTGATAAATCAAAGGAAAATTATTC

rabbit1_chro.50457 (1) TCCCGAATATAGTATTAATAATGATAAACTCGAGCTTGATAAATTAAAAGAAAATTATTC

rabbit2_chro.50457 (1) TCCCGAATATAGTATTAATAATGATAAACTCGAGCTTGATAAATTAAAAGAAAATTATTC

rabbit3_chro.50457 (1) TCCCGAATATAGTATTAATAATGATAAACTCGAGCTTGATAAATTAAAAGAAAATTATTC

rabbit4_chro.50457 (1) TCCCGAATATAGTATTAATAATGATAAACTCGAGCTTGATAAATTAAAAGAAAATTATTC

C.meleagridis_chro.50457 (1) TCCCGAATATAACATTAATAATGATAAGCTTGAGCTTAATAAGACAAAAATAAATTATTC

61 120

ch2_chro.50457 (61) ACAAAATAATGAATTACTTCCATATTCATCTTTATGCGTTGTTAAAAAGAAGCCTCTCCC

ch3_chro.50457 (61) ACAAAATAATGAATTACTTCCATATTCATCTTTATGCGTTGTTAAAAAGAAGCCTCTCCC

ch4_chro.50457 (61) ACAAAATAATGAATTACTTCCATATTCATCTTTATGCGTTGTTAAAAAGAAGCCTCTCCC

TU502_chro.50457 (61) ACAAAATAATGAATTACTTCCATATTCATCTTTATGCGTTGTTAAAAAGAAGCCTCTCCC

cp2_chro.50457 (61) ACAAAATAATGAATTACTTCCATATTCATCTTTATGCATTGTTAAAAAGAAGCCTCTCCC

cp3_chro.50457 (61) ACAAAATAATGAATTACTTCCATATTCATCTTTATGCATTGTTAAAAAGAAGCCTCTCCC

cp4_chro.50457 (61) ACAAAATAATGAATTACTTCCATATTCATCTTTATGCATTGTTAAAAAGAAGCCTCTCCC

Iowa_chro.50457 (61) ACAAAATAATGAATTACTTCCATATTCATCTTTATGCATTGTTAAAAAGAAGCCTCTCCC

Moredun_chro.50457 (61) ACAAAATAATGAATTACTTCCATATTCATCTTTATGCATTGTTAAAAAGAAGCCTCTCCC

w65_chro.50457 (61) ACAAAATAATGAATTACTTCCATATTCATCTTTATGCATTGTTAAAAAGAAGCCTCTCCC

w66_chro.50457 (61) ACAAAATAATGAATTACTTCCATATTCATCTTTATGCATTGTTAAAAAGAAGCCTCTCCC

w67_chro.50457 (61) ACAAAATAATGAATTACTTCCATATTCATCTTTATGCATTGTTAAAAAGAAGCCTCTCCC

w70_chro.50457 (61) ACAAAATAATGAATTACTTCCATATTCATCTTTATGCATTGTTAAAAAGAAGCCTCTCCC

rabbit1_chro.50457 (61) ACAAAATAATGAATTACTTCCATATTCATCTTTATGCGTTGTTAAAAAGAAGCCTCTCCC

rabbit2_chro.50457 (61) ACAAAATAATGAATTACTTCCATATTCATCTTTATGCGTTGTTAAAAAGAAGCCTCTCCC

rabbit3_chro.50457 (61) ACAAAATAATGAATTACTTCCATATTCATCTTTATGCGTTGTTAAAAAGAAGCCTCTCCC

rabbit4_chro.50457 (61) ACAAAATAATGAATTACTTCCATATTCATCTTTATGCGTTGTTAAAAAGAAGCCTCTCCC

C.meleagridis_chro.50457 (61) ACAAAATAATGAGTTACTTCCATACTCATCCTTGTGTGTTGTTAAAAGGAAGCCTCTCCC

121 180

ch2_chro.50457 (121) TGTTATATCTTTTTGGTTAAGTCAAACTGATTCGAATGATAATAAAGTCGAGCCTATAAA

ch3_chro.50457 (121) TGTTATATCTTTTTGGTTAAGTCAAACTGATTCGAATGATAATAAAGTCGAGCCTATAAA

ch4_chro.50457 (121) TGTTATATCTTTTTGGTTAAGTCAAACTGATTCGAATGATAATAAAGTCGAGCCTATAAA

TU502_chro.50457 (121) TGTTATATCTTTTTGGTTAAGTCAAACTGATTCGAATGATAATAAAGTCGAGCCTATAAA

cp2_chro.50457 (121) TGTTATATCTTTTTGGTTAAGTCAAACTGATTCAAATGATGGTAAAGTTGAGCCTATAAA

cp3_chro.50457 (121) TGTTATATCTTTTTGGTTAAGTCAAACTGATTCAAATGATGGTAAAGTTGAGCCTATAAA

cp4_chro.50457 (121) TGTTATATCTTTTTGGTTAAGTCAAACTGATTCAAATGATGGTAAAGTTGAGCCTATAAA

Iowa_chro.50457 (121) TGTTATATCTTTTTGGTTAAGTCAAACTGATTCAAATGATGGTAAAGTTGAGCCTATAAA

Moredun_chro.50457 (121) TGTTATATCTTTTTGGTTAAGTCAAACTGATTCAAATGATGGTAAAGTTGAGCCTATAAA

w65_chro.50457 (121) TGTTATATCTTTTTGGTTAAGTCAAACTGATTCAAATGATGGTAAAGTTGAGCCTATAAA

w66_chro.50457 (121) TGTTATATCTTTTTGGTTAAGTCAAACTGATTCAAATGATGGTAAAGTTGAGCCTATAAA

w67_chro.50457 (121) TGTTATATCTTTTTGGTTAAGTCAAACTGATTCAAATGATGGTAAAGTTGAGCCTATAAA

w70_chro.50457 (121) TGTTATATCTTTTTGGTTAAGTCAAACTGATTCAAATGATGGTAAAGTTGAGCCTATAAA

rabbit1_chro.50457 (121) TGTTATATCTTTTTGGTTAAGTCAAACTGATTCGAATGATAATAAAGTCGAGCCTATAAA

rabbit2_chro.50457 (121) TGTTATATCTTTTTGGTTAAGTCAAACTGATTCGAATGATAATAAAGTCGAGCCTATAAA

rabbit3_chro.50457 (121) TGTTATATCTTTTTGGTTAAGTCAAACTGATTCGAATGATAATAAAGTCGAGCCTATAAA

rabbit4_chro.50457 (121) TGTTATATCTTTTTGGTTAAGTCAAACTGATTCGAATGATAATAAAGTCGAGCCTATAAA

C.meleagridis_chro.50457 (121) TGTTATATCTTTTTGGTTAAGCCAAGCTGATTCAAATAATGATAAAGTGGAGCCTATGAA

181 240

ch2_chro.50457 (181) GATCTTTTTGGATGATTATATTATTCATTTTAATAATATTGACTATCTATGTGTAATCAA

ch3_chro.50457 (181) GATCTTTTTGGATGATTATATTATTCATTTTAATAATATTGACTATCTATGTGTAATCAA

ch4_chro.50457 (181) GATCTTTTTGGATGATTATATTATTCATTTTAATAATATTGACTATCTATGTGTAATCAA

TU502_chro.50457 (181) GATCTTTTTGGATGATTATATTATTCATTTTAATAATATTGACTATCTATGTGTAATCAA

cp2_chro.50457 (181) GATCTTTTTGGATGATTATATTATTCATTTTAATAATATTGACTATCTATGTGTAATTAA

cp3_chro.50457 (181) GATCTTTTTGGATGATTATATTATTCATTTTAATAATATTGACTATCTATGTGTAATTAA

cp4_chro.50457 (181) GATCTTTTTGGATGATTATATTATTCATTTTAATAATATTGACTATCTATGTGTAATTAA

Iowa_chro.50457 (181) GATCTTTTTGGATGATTATATTATTCATTTTAATAATATTGACTATCTATGTGTAATTAA

Moredun_chro.50457 (181) GATCTTTTTGGATGATTATATTATTCATTTTAATAATATTGACTATCTATGTGTAATTAA

w65_chro.50457 (181) GATCTTTTTGGATGATTATATTATTCATTTTAATAATATTGACTATCTATGTGTAATTAA

w66_chro.50457 (181) GATCTTTTTGGATGATTATATTATTCATTTTAATAATATTGACTATCTATGTGTAATTAA

w67_chro.50457 (181) GATCTTTTTGGATGATTATATTATTCATTTTAATAATATTGACTATCTATGTGTAATTAA

w70_chro.50457 (181) GATCTTTTTGGATGATTATATTATTCATTTTAATAATATTGACTATCTATGTGTAATTAA

rabbit1_chro.50457 (181) GATCTTTTTGGATGATTATATTATTCATTTTAATAATATTGACTATCTATGTGTAATCAA

rabbit2_chro.50457 (181) GATCTTTTTGGATGATTATATTATTCATTTTAATAATATTGACTATCTATGTGTAATCAA

rabbit3_chro.50457 (181) GATCTTTTTGGATGATTATATTATTCATTTTAATAATATTGACTATCTATGTGTAATCAA

rabbit4_chro.50457 (181) GATCTTTTTGGATGATTATATTATTCATTTTAATAATATTGACTATCTATGTGTAATCAA

C.meleagridis_chro.50457 (181) GATCTTTTTGGATGACTATATTATTCATTTTAATAATATTGACTATTTGTGTGTAATTAA

241 300

ch2_chro.50457 (241) TTCAACACTAGGTACTTTTGGAAAAGTTAATTACATACCTTACAGTGAAGGTAATAGTTA

ch3_chro.50457 (241) TTCAACACTAGGTACTTTTGGAAAAGTTAATTACATACCTTACAGTGAAGGTAATAGTTA

ch4_chro.50457 (241) TTCAACACTAGGTACTTTTGGAAAAGTTAATTACATACCTTACAGTGAAGGTAATAGTTA

TU502_chro.50457 (241) TTCAACACTAGGTACTTTTGGAAAAGTTAATTACATACCTTACAGTGAAGGTAATAGTTA

cp2_chro.50457 (241) TTCAACACTAGGTACTTTTGGAAAAATTAATTACATACCTTACAGTGAAGGTAATAGTTA

cp3_chro.50457 (241) TTCAACACTAGGTACTTTTGGAAAAATTAATTACATACCTTACAGTGAAGGTAATAGTTA

cp4_chro.50457 (241) TTCAACACTAGGTACTTTTGGAAAAATTAATTACATACCTTACAGTGAAGGTAATAGTTA

Iowa_chro.50457 (241) TTCAACACTAGGTACTTTTGGAAAAATTAATTACATACCTTACAGTGAAGGTAATAGTTA

Moredun_chro.50457 (241) TTCAACACTAGGTACTTTTGGAAAAATTAATTACATACCTTACAGTGAAGGTAATAGTTA

w65_chro.50457 (241) TTCAACACTAGGTACTTTTGGAAAAATTAATTACATACCTTACAGTGAAGGTAATAGTTA

w66_chro.50457 (241) TTCAACACTAGGTACTTTTGGAAAAATTAATTACATACCTTACAGTGAAGGTAATAGTTA

w67_chro.50457 (241) TTCAACACTAGGTACTTTTGGAAAAATTAATTACATACCTTACAGTGAAGGTAATAGTTA

w70_chro.50457 (241) TTCAACACTAGGTACTTTTGGAAAAATTAATTACATACCTTACAGTGAAGGTAATAGTTA

rabbit1_chro.50457 (241) TTCAACACTAGGTACTTTTGGAAAAGTTAATTACATACCTTACAGTGAAGGTAATAGTTA

rabbit2_chro.50457 (241) TTCAACACTAGGTACTTTTGGAAAAGTTAATTACATACCTTACAGTGAAGGTAATAGTTA

rabbit3_chro.50457 (241) TTCAACACTAGGTACTTTTGGAAAAGTTAATTACATACCTTACAGTGAAGGTAATAGTTA

rabbit4_chro.50457 (241) TTCAACACTAGGTACTTTTGGAAAAGTTAATTACATACCTTACAGTGAAGGTAATAGTTA

C.meleagridis_chro.50457 (241) CTCAACTCTAGGTACTTTTGGAAAGGTCAACTACATACCTTATAGTGAAGGTAATAGTTA

301 360

ch2_chro.50457 (301) TAACTACTTCACTCCTTATTATTCATCCTCTAATACTCCCTTGATACTCTTTGGTTCTCT

ch3_chro.50457 (301) TAACTACTTCACTCCTTATTATTCATCCTCTAATACTCCCTTGATACTCTTTGGTTCTCT

ch4_chro.50457 (301) TAACTACTTCACTCCTTATTATTCATCCTCTAATACTCCCTTGATACTCTTTGGTTCTCT

TU502_chro.50457 (301) TAACTACTTCACTCCTTATTATTCATCCTCTAATACTCCCTTGATACTCTTTGGTTCTCT

cp2_chro.50457 (301) TAACTACTTTACTCCTTATTATTCATCCTCTAATACTCCTTTGATACTCCTTGGTTCTCT

cp3_chro.50457 (301) TAACTACTTTACTCCTTATTATTCATCCTCTAATACTCCTTTGATACTCCTTGGTTCTCT

cp4_chro.50457 (301) TAACTACTTTACTCCTTATTATTCATCCTCTAATACTCCTTTGATACTCCTTGGTTCTCT

Iowa_chro.50457 (301) TAACTACTTTACTCCTTATTATTCATCCTCTAATACTCCTTTGATACTCCTTGGTTCTCT

Moredun_chro.50457 (301) TAACTACTTTACTCCTTATTATTCATCCTCTAATACTCCTTTGATACTCCTTGGTTCTCT

w65_chro.50457 (301) TAACTACTTTACTCCTTATTATTCATCCTCTAATACTCCTTTGATACTCCTTGGTTCTCT

w66_chro.50457 (301) TAACTACTTTACTCCTTATTATTCATCCTCTAATACTCCTTTGATACTCCTTGGTTCTCT

w67_chro.50457 (301) TAACTACTTTACTCCTTATTATTCATCCTCTAATACTCCTTTGATACTCCTTGGTTCTCT

w70_chro.50457 (301) TAACTACTTTACTCCTTATTATTCATCCTCTAATACTCCTTTGATACTCCTTGGTTCTCT

rabbit1_chro.50457 (301) TAACTACTTCACTCCTTATTATTCATCCTCTAATACTCCCTTGATACTCTTTGGTTCTCT

rabbit2_chro.50457 (301) TAACTACTTCACTCCTTATTATTCATCCTCTAATACTCCCTTGATACTCTTTGGTTCTCT

rabbit3_chro.50457 (301) TAACTACTTCACTCCTTATTATTCATCCTCTAATACTCCCTTGATACTCTTTGGTTCTCT

rabbit4_chro.50457 (301) TAACTACTTCACTCCTTATTATTCATCCTCTAATACTCCCTTGATACTCTTTGGTTCTCT

C.meleagridis_chro.50457 (301) TAACTATTTTACCCCGTATTATTCATCCTCCAATGCTCCTTTGATACTCTTTGGTTCTCT

361 373

ch2_chro.50457 (361) TTTTCTCAAATCA

ch3_chro.50457 (361) TTTTCTCAAATCA

ch4_chro.50457 (361) TTTTCTCAAATCA

TU502_chro.50457 (361) TTTTCTCAAATCA

cp2_chro.50457 (361) TTTTCTCAAATCA

cp3_chro.50457 (361) TTTTCTCAAATCA

cp4_chro.50457 (361) TTTTCTCAAATCA

Iowa_chro.50457 (361) TTTTCTCAAATCA

Moredun_chro.50457 (361) TTTTCTCAAATCA

w65_chro.50457 (361) TTTTCTCAAATCA

w66_chro.50457 (361) TTTTCTCAAATCA

w67_chro.50457 (361) TTTTCTCAAATCA

w70_chro.50457 (361) TTTTCTCAAATCA

rabbit1_chro.50457 (361) TTTTCTCAAATCA

rabbit2_chro.50457 (361) TTTTCTCAAATCA

rabbit3_chro.50457 (361) TTTTCTCAAATCA

rabbit4_chro.50457 (361) TTTTCTCAAATCA

C.meleagridis_chro.50457 (361) TTTTCTCAAATCA

**Cgd6_5020 gene PCR products**

1 60

ch2_cgd6_5020 (1) ACGATTGCTCTTTATCTTTATGGGATCTCAGAACAAGTTGTACTGATGAGCCAATAATGA

ch3_cgd6_5020 (1) ACGATTGCTCTTTATCTTTATGGGATCTCAGAACAAGTTGTACTGATGAGCCAATAATGA

ch4_cgd6_5020 (1) ACGATTGCTCTTTATCTTTATGGGATCTCAGAACAAGTTGTACTGATGAGCCAATAATGA

TU502_cgd6_5020 (1) ACGATTGCTCTTTATCTTTATGGGATCTCAGAACAAGTTGTACTGATGAGCCAATAATGA

rabbit1_cgd6_5020 (1) ACGATTGCTCTTTATCTTTATGGGATCTCAGAACAAGTTGTACTGATGAGCCAATAATGA

rabbit2_cgd6_5020 (1) ACGATTGCTCTTTATCTTTATGGGATCTCAGAACAAGTTGTACTGATGAGCCAATAATGA

rabbit3_cgd6_5020 (1) ACGATTGCTCTTTATCTTTATGGGATCTCAGAACAAGTTGTACTGATGAGCCAATAATGA

rabbit4_cgd6_5020 (1) ACGATTGCTCTTTATCTTTATGGGATCTCAGAACAAGTTGTACTGATGAGCCAATAATGA

cp2_cgd6_5020 (1) ACGATTGCTCTTTATCTTTATGGGATCTCAGAACAAGTTGTATTGATGAGCCAATAATGA

cp3_cgd6_5020 (1) ACGATTGCTCTTTATCTTTATGGGATCTCAGAACAAGTTGTATTGATGAGCCAATAATGA

cp4_cgd6_5020 (1) ACGATTGCTCTTTATCTTTATGGGATCTCAGAACAAGTTGTATTGATGAGCCAATAATGA

Iowa_cgd6_5020 (1) ACGATTGCTCTTTATCTTTATGGGATCTCAGAACAAGTTGTATTGATGAGCCAATAATGA

Moredun_cgd6_5020 (1) ACGATTGCTCTTTATCTTTATGGGATCTCAGAACAAGTTGTATTGATGAGCCAATAATGA

w65_cgd6_5020 (1) ACGATTGCTCTTTATCTTTATGGGATCTCAGAACAAGTTGTATTGATGAGCCAATAATGA

w66_cgd6_5020 (1) ACGATTGCTCTTTATCTTTATGGGATCTCAGAACAAGTTGTATTGATGAGCCAATAATGA

w67_cgd6_5020 (1) ACGATTGCTCTTTATCTTTATGGGATCTCAGAACAAGTTGTATTGATGAGCCAATAATGA

w70_cgd6_5020 (1) ACGATTGCTCTTTATCTTTATGGGATCTCAGAACAAGTTGTATTGATGAGCCAATAATGA

C.meleagridis_cgd6_5020 (1) ACGATTGCTCTTTATCTTTATGGGACCTCAGAACAAGTTTTACTGATGAGCCGATAATGA

61 120

ch2_cgd6_5020 (61) AGAACAAGAAATCCCATTCTATGGGTGTAACTTGTATCCAAAAGTCGAATAGAAATCATC

ch3_cgd6_5020 (61) AGAACAAGAAATCCCATTCTATGGGTGTAACTTGTATCCAAAAGTCGAATAGAAATCATC

ch4_cgd6_5020 (61) AGAACAAGAAATCCCATTCTATGGGTGTAACTTGTATCCAAAAGTCGAATAGAAATCATC

TU502_cgd6_5020 (61) AGAACAAGAAATCCCATTCTATGGGTGTAACTTGTATCCAAAAGTCGAATAGAAATCATC

rabbit1_cgd6_5020 (61) AGAACAAGAAATCCCATTCTATGGGTGTAACTTGTATCCAAAAGTCGAATAGAAATCATC

rabbit2_cgd6_5020 (61) AGAACAAGAAATCCCATTCTATGGGTGTAACTTGTATCCAAAAGTCGAATAGAAATCATC

rabbit3_cgd6_5020 (61) AGAACAAGAAATCCCATTCTATGGGTGTAACTTGTATCCAAAAGTCGAATAGAAATCATC

rabbit4_cgd6_5020 (61) AGAACAAGAAATCCCATTCTATGGGTGTAACTTGTATCCAAAAGTCGAATAGAAATCATC

cp2_cgd6_5020 (61) AGAACAAGAAATCCCATTCTATGGGTGTAACTTGTATCCAAAAGTCGAATAGAAATCATC

cp3_cgd6_5020 (61) AGAACAAGAAATCCCATTCTATGGGTGTAACTTGTATCCAAAAGTCGAATAGAAATCATC

cp4_cgd6_5020 (61) AGAACAAGAAATCCCATTCTATGGGTGTAACTTGTATCCAAAAGTCGAATAGAAATCATC

Iowa_cgd6_5020 (61) AGAACAAGAAATCCCATTCTATGGGTGTAACTTGTATCCAAAAGTCGAATAGAAATCATC

Moredun_cgd6_5020 (61) AGAACAAGAAATCCCATTCTATGGGTGTAACTTGTATCCAAAAGTCGAATAGAAATCATC

w65_cgd6_5020 (61) AGAACAAGAAATCCCATTCTATGGGTGTAACTTGTATCCAAAAGTCGAATAGAAATCATC

w66_cgd6_5020 (61) AGAACAAGAAATCCCATTCTATGGGTGTAACTTGTATCCAAAAGTCGAATAGAAATCATC

w67_cgd6_5020 (61) AGAACAAGAAATCCCATTCTATGGGTGTAACTTGTATCCAAAAGTCGAATAGAAATCATC

w70_cgd6_5020 (61) AGAACAAGAAATCCCATTCTATGGGTGTAACTTGTATCCAAAAGTCGAATAGAAATCATC

C.meleagridis_cgd6_5020 (61) AGAACAAGAAATCCCATTCTATGGGTGTAACTTGTATCCAAAAGTCGAATAGAAATCATC

121 180

ch2_cgd6_5020 (121) AATTTTGGTCAGGTAGTTACGACGAAACCCTTAGATTCTGGGATTTTAGGATGATAAACT

ch3_cgd6_5020 (121) AATTTTGGTCAGGTAGTTACGACGAAACCCTTAGATTCTGGGATTTTAGGATGATAAACT

ch4_cgd6_5020 (121) AATTTTGGTCAGGTAGTTACGACGAAACCCTTAGATTCTGGGATTTTAGGATGATAAACT

TU502_cgd6_5020 (121) AATTTTGGTCAGGTAGTTACGACGAAACCCTTAGATTCTGGGATTTTAGGATGATAAACT

rabbit1_cgd6_5020 (121) AATTTTGGTCAGGTAGTTACGACGAAACCCTTAGATTCTGGGATTTTAGGATGATAAACT

rabbit2_cgd6_5020 (121) AATTTTGGTCAGGTAGTTACGACGAAACCCTTAGATTCTGGGATTTTAGGATGATAAACT

rabbit3_cgd6_5020 (121) AATTTTGGTCAGGTAGTTACGACGAAACCCTTAGATTCTGGGATTTTAGGATGATAAACT

rabbit4_cgd6_5020 (121) AATTTTGGTCAGGTAGTTACGACGAAACCCTTAGATTCTGGGATTTTAGGATGATAAACT

cp2_cgd6_5020 (121) AATTTTGGTCAGGAAGTTACGACGAAACCCTTAGATTCTGGGATTTTAGGATGATAAACT

cp3_cgd6_5020 (121) AATTTTGGTCAGGAAGTTACGACGAAACCCTTAGATTCTGGGATTTTAGGATGATAAACT

cp4_cgd6_5020 (121) AATTTTGGTCAGGAAGTTACGACGAAACCCTTAGATTCTGGGATTTTAGGATGATAAACT

Iowa_cgd6_5020 (121) AATTTTGGTCAGGAAGTTACGACGAAACCCTTAGATTCTGGGATTTTAGGATGATAAACT

Moredun_cgd6_5020 (121) AATTTTGGTCAGGAAGTTACGACGAAACCCTTAGATTCTGGGATTTTAGGATGATAAACT

w65_cgd6_5020 (121) AATTTTGGTCAGGAAGTTACGACGAAACCCTTAGATTCTGGGATTTTAGGATGATAAACT

w66_cgd6_5020 (121) AATTTTGGTCAGGAAGTTACGACGAAACCCTTAGATTCTGGGATTTTAGGATGATAAACT

w67_cgd6_5020 (121) AATTTTGGTCAGGAAGTTACGACGAAACCCTTAGATTCTGGGATTTTAGGATGATAAACT

w70_cgd6_5020 (121) AATTTTGGTCAGGAAGTTACGACGAAACCCTTAGATTCTGGGATTTTAGGATGATAAACT

C.meleagridis_cgd6_5020 (121) AATTTTGGTCAGGAAGTTACGACGAAACCCTTAGATTCTGGGATTCTAGGATGATAAACT

181 240

ch2_cgd6_5020 (181) CTCCTATTTATGAGCACAAAACTAATGGAGGAATTTGGAGAATTAACCAATTTGAAGATT

ch3_cgd6_5020 (181) CTCCTATTTATGAGCACAAAACTAATGGAGGAATTTGGAGAATTAACCAATTTGAAGATT

ch4_cgd6_5020 (181) CTCCTATTTATGAGCACAAAACTAATGGAGGAATTTGGAGAATTAACCAATTTGAAGATT

TU502_cgd6_5020 (181) CTCCTATTTATGAGCACAAAACTAATGGAGGAATTTGGAGAATTAACCAATTTGAAGATT

rabbit1_cgd6_5020 (181) CTCCTATTTATGAGCACAAAACTAATGGAGGAATTTGGAGAATTAACCAATTTGAAGATT

rabbit2_cgd6_5020 (181) CTCCTATTTATGAGCACAAAACTAATGGAGGAATTTGGAGAATTAACCAATTTGAAGATT

rabbit3_cgd6_5020 (181) CTCCTATTTATGAGCACAAAACTAATGGAGGAATTTGGAGAATTAACCAATTTGAAGATT

rabbit4_cgd6_5020 (181) CTCCTATTTATGAGCACAAAACTAATGGAGGAATTTGGAGAATTAACCAATTTGAAGATT

cp2_cgd6_5020 (181) CTCCTATTTATGAGCACAAAACTAATGGAGGAATTTGGAGAATTAACCAATTTGAAGATT

cp3_cgd6_5020 (181) CTCCTATTTATGAGCACAAAACTAATGGAGGAATTTGGAGAATTAACCAATTTGAAGATT

cp4_cgd6_5020 (181) CTCCTATTTATGAGCACAAAACTAATGGAGGAATTTGGAGAATTAACCAATTTGAAGATT

Iowa_cgd6_5020 (181) CTCCTATTTATGAGCACAAAACTAATGGAGGAATTTGGAGAATTAACCAATTTGAAGATT

Moredun_cgd6_5020 (181) CTCCTATTTATGAGCACAAAACTAATGGAGGAATTTGGAGAATTAACCAATTTGAAGATT

w65_cgd6_5020 (181) CTCCTATTTATGAGCACAAAACTAATGGAGGAATTTGGAGAATTAACCAATTTGAAGATT

w66_cgd6_5020 (181) CTCCTATTTATGAGCACAAAACTAATGGAGGAATTTGGAGAATTAACCAATTTGAAGATT

w67_cgd6_5020 (181) CTCCTATTTATGAGCACAAAACTAATGGAGGAATTTGGAGAATTAACCAATTTGAAGATT

w70_cgd6_5020 (181) CTCCTATTTATGAGCACAAAACTAATGGAGGAATTTGGAGAATTAACCAATTTGAAGATT

C.meleagridis_cgd6_5020 (181) TTCCTATTTATGAGCACAAAACTAATGGAGGAATTTGGAGAATTAACCAATTTGAAGATT

241

ch2_cgd6_5020 (241) ACCTTGGA

ch3_cgd6_5020 (241) ACCTTGGA

ch4_cgd6_5020 (241) ACCTTGGA

TU502_cgd6_5020 (241) ACCTTGGA

rabbit1_cgd6_5020 (241) ACCTTGGA

rabbit2_cgd6_5020 (241) ACCTTGGA

rabbit3_cgd6_5020 (241) ACCTTGGA

rabbit4_cgd6_5020 (241) ACCTTGGA

cp2_cgd6_5020 (241) ACCTTGGA

cp3_cgd6_5020 (241) ACCTTGGA

cp4_cgd6_5020 (241) ACCTTGGA

Iowa_cgd6_5020 (241) ACCTTGGA

Moredun_cgd6_5020 (241) ACCTTGGA

w65_cgd6_5020 (241) ACCTTGGA

w66_cgd6_5020 (241) ACCTTGGA

w67_cgd6_5020 (241) ACCTTGGA

w70_cgd6_5020 (241) ACCTTGGA

C.meleagridis_cgd6_5020 (241) ACCTTGGA

**COWP gene PCR products**

1 60

ch2_COWP (1) CAGGCATTATCTTGTAATACTGTACCTGGAGGGCAGACAGGTTGAGTTGGAGCAGAACTA

ch3_COWP (1) CAGGCATTATCTTGTAATACTGTACCTGGAGGGCAGACAGGTTGAGTTGGAGCAGAACTA

ch4_COWP (1) CAGGCATTATCTTGTAATACTGTACCTGGAGGGCAGACAGGTTGAGTTGGAGCAGAACTA

TU502_COWP (1) CAGGCATTATCTTGTAATACTGTACCTGGAGGGCAGACAGGTTGAGTTGGAGCAGAACTA

rabbit1_COWP (1) CAGGCATTATCTTGTAATACTGTACCTGGAGGGCAGACAGGTTGAGTTGGAGCAGAACTA

rabbit2_COWP (1) CAGGCATTATCTTGTAATACTGTACCTGGAGGGCAGACAGGTTGAGTTGGAGCAGAACTA

rabbit3_COWP (1) CAGGCATTATCTTGTAATACTGTACCTGGAGGGCAGACAGGTTGAGTTGGAGCAGAACTA

rabbit4_COWP (1) CAGGCATTATCTTGTAATACTGTACCTGGAGGGCAGACAGGTTGAGTTGGAGCAGAACTA

cp2_COWP (1) CAGGCATTATCTTGTAATACTGTACCTGGAGGGCAGACAGGTTGAGTTGGAGCAGAACTA

cp3_COWP (1) CAGGCATTATCTTGTAATACTGTACCTGGAGGGCAGACAGGTTGAGTTGGAGCAGAACTA

cp4_COWP (1) CAGGCATTATCTTGTAATACTGTACCTGGAGGGCAGACAGGTTGAGTTGGAGCAGAACTA

Iowa_COWP (1) CAGGCATTATCTTGTAATACTGTACCTGGAGGGCAGACAGGTTGAGTTGGAGCAGAACTA

Moredun_COWP (1) CAGGCATTATCTTGTAATACTGTACCTGGAGGGCAGACAGGTTGAGTTGGAGCAGAACTA

w65_COWP (1) CAGGCATTATCTTGTAATACTGTACCTGGAGGGCAGACAGGTTGAGTTGGAGCAGAACTA

w66_COWP (1) CAGGCATTATCTTGTAATACTGTACCTGGAGGGCAGACAGGTTGAGTTGGAGCAGAACTA

w67_COWP (1) CAGGCATTATCTTGTAATACTGTACCTGGAGGGCAGACAGGTTGAGTTGGAGCAGAACTA

w70_COWP (1) CAGGCATTATCTTGTAATACTGTACCTGGAGGGCAGACAGGTTGAGTTGGAGCAGAACTA

C.meleagridis_COWP (1) CAGGCATTATCTTGTAGCACTGTACCTGTAGGGCATACAGGTTGTGTTGGAGCGGAACTA

61 120

ch2_COWP (61) GTTTGAATACACTGGAGGCCTTGTAAAATGAAATTTGGTGGGCATTCCTTTGCAGGAGCT

ch3_COWP (61) GTTTGAATACACTGGAGGCCTTGTAAAATGAAATTTGGTGGGCATTCCTTTGCAGGAGCT

ch4_COWP (61) GTTTGAATACACTGGAGGCCTTGTAAAATGAAATTTGGTGGGCATTCCTTTGCAGGAGCT

TU502_COWP (61) GTTTGAATACACTGGAGGCCTTGTAAAATGAAATTTGGTGGGCATTCCTTTGCAGGAGCT

rabbit1_COWP (61) GTTTGAATACACTGGAGGCCTTGTAAAATGAAATTTGGTGGGCATTCCTTTGCAGGAGCT

rabbit2_COWP (61) GTTTGAATACACTGGAGGCCTTGTAAAATGAAATTTGGTGGGCATTCCTTTGCAGGAGCT

rabbit3_COWP (61) GTTTGAATACACTGGAGGCCTTGTAAAATGAAATTTGGTGGGCATTCCTTTGCAGGAGCT

rabbit4_COWP (61) GTTTGAATACACTGGAGGCCTTGTAAAATGAAATTTGGTGGGCATTCCTTTGCAGGAGCT

cp2_COWP (61) GTTTGTATACATTGGAGGCCTTGTAAAATGAAATTTGGTGGGCATTCCTTTGCAGGAGCT

cp3_COWP (61) GTTTGTATACATTGGAGGCCTTGTAAAATGAAATTTGGTGGGCATTCCTTTGCAGGAGCT

cp4_COWP (61) GTTTGTATACATTGGAGGCCTTGTAAAATGAAATTTGGTGGGCATTCCTTTGCAGGAGCT

Iowa_COWP (61) GTTTGTATACATTGGAGGCCTTGTAAAATGAAATTTGGTGGGCATTCCTTTGCAGGAGCT

Moredun_COWP (61) GTTTGTATACATTGGAGGCCTTGTAAAATGAAATTTGGTGGGCATTCCTTTGCAGGAGCT

w65_COWP (61) GTTTGTATACATTGGAGGCCTTGTAAAATGAAATTTGGTGGGCATTCCTTTGCAGGAGCT

w66_COWP (61) GTTTGTATACATTGGAGGCCTTGTAAAATGAAATTTGGTGGGCATTCCTTTGCAGGAGCT

w67_COWP (61) GTTTGTATACATTGGAGGCCTTGTAAAATGAAATTTGGTGGGCATTCCTTTGCAGGAGCT

w70_COWP (61) GTTTGTATACATTGGAGGCCTTGTAAAATGAAATTTGGTGGGCATTCCTTTGCAGGAGCT

C.meleagridis_COWP (61) GTTTGAATACACTGGAGGCCTTGTAATATGAAATTTGGTGGGCATTCCTTTGCAGGAGCT

121 180

ch2_COWP (121) ACATATAGTACACAATCATCTCCTGAATCTGTATATCCTGGTGGGCAGACCATATCAATA

ch3_COWP (121) ACATATAGTACACAATCATCTCCTGAATCTGTATATCCTGGTGGGCAGACCATATCAATA

ch4_COWP (121) ACATATAGTACACAATCATCTCCTGAATCTGTATATCCTGGTGGGCAGACCATATCAATA

TU502_COWP (121) ACATATAGTACACAATCATCTCCTGAATCTGTATATCCTGGTGGGCAGACCATATCAATA

rabbit1_COWP (121) ACATATAGTACACAATCATCTCCTGAATCTGTATATCCTGGTGGGCAGACCATATCAATA

rabbit2_COWP (121) ACATATAGTACACAATCATCTCCTGAATCTGTATATCCTGGTGGGCAGACCATATCAATA

rabbit3_COWP (121) ACATATAGTACACAATCATCTCCTGAATCTGTATATCCTGGTGGGCAGACCATATCAATA

rabbit4_COWP (121) ACATATAGTACACAATCATCTCCTGAATCTGTATATCCTGGTGGGCAGACCATATCAATA

cp2_COWP (121) ACATATAGTACACAATCATCTCCTGAGTCTGTATATCCTGGTGGGCAGACCATATCAATA

cp3_COWP (121) ACATATAGTACACAATCATCTCCTGAGTCTGTATATCCTGGTGGGCAGACCATATCAATA

cp4_COWP (121) ACATATAGTACACAATCATCTCCTGAGTCTGTATATCCTGGTGGGCAGACCATATCAATA

Iowa_COWP (121) ACATATAGTACACAATCATCTCCTGAGTCTGTATATCCTGGTGGGCAGACCATATCAATA

Moredun_COWP (121) ACATATAGTACACAATCATCTCCTGAGTCTGTATATCCTGGTGGGCAGACCATATCAATA

w65_COWP (121) ACATATAGTACACAATCATCTCCTGAGTCTGTATATCCTGGTGGGCAGACCATATCAATA

w66_COWP (121) ACATATAGTACACAATCATCTCCTGAGTCTGTATATCCTGGTGGGCAGACCATATCAATA

w67_COWP (121) ACATATAGTACACAATCATCTCCTGAGTCTGTATATCCTGGTGGGCAGACCATATCAATA

w70_COWP (121) ACATATAGTACACAATCATCTCCTGAGTCTGTATATCCTGGTGGGCAGACCATATCAATA

C.meleagridis_COWP (121) ACATATAGAACACAATCATCTCCTGAGTCTGTATATCCTGGTGGGCAGACCATATCAATA

181 240

ch2_COWP (181) TTTTTAATCACTTTACATTTTCCATTTTCAAATATTGAATTAGGTGGGCATGTCGATTCT

ch3_COWP (181) TTTTTAATCACTTTACATTTTCCATTTTCAAATATTGAATTAGGTGGGCATGTCGATTCT

ch4_COWP (181) TTTTTAATCACTTTACATTTTCCATTTTCAAATATTGAATTAGGTGGGCATGTCGATTCT

TU502_COWP (181) TTTTTAATCACTTTACATTTTCCATTTTCAAATATTGAATTAGGTGGGCATGTCGATTCT

rabbit1_COWP (181) TTTTTAATCACTTTACATTTTCCATTTTCAAATATTGAATTAGGTGGGCATGTCGATTCT

rabbit2_COWP (181) TTTTTAATCACTTTACATTTTCCATTTTCAAATATTGAATTAGGTGGGCATGTCGATTCT

rabbit3_COWP (181) TTTTTAATCACTTTACATTTTCCATTTTCAAATATTGAATTAGGTGGGCATGTCGATTCT

rabbit4_COWP (181) TTTTTAATCACTTTACATTTTCCATTTTCAAATATTGAATTAGGTGGGCATGTCGATTCT

cp2_COWP (181) TTTTTAATCACTTTACATTTTCCATTTTCAAATATTGAATTAGGTGGGCATGTCGATTCT

cp3_COWP (181) TTTTTAATCACTTTACATTTTCCATTTTCAAATATTGAATTAGGTGGGCATGTCGATTCT

cp4_COWP (181) TTTTTAATCACTTTACATTTTCCATTTTCAAATATTGAATTAGGTGGGCATGTCGATTCT

Iowa_COWP (181) TTTTTAATCACTTTACATTTTCCATTTTCAAATATTGAATTAGGTGGGCATGTCGATTCT

Moredun_COWP (181) TTTTTAATCACTTTACATTTTCCATTTTCAAATATTGAATTAGGTGGGCATGTCGATTCT

w65_COWP (181) TTTTTAATCACTTTACATTTTCCATTTTCAAATATTGAATTAGGTGGGCATGTCGATTCT

w66_COWP (181) TTTTTAATCACTTTACATTTTCCATTTTCAAATATTGAATTAGGTGGGCATGTCGATTCT

w67_COWP (181) TTTTTAATCACTTTACATTTTCCATTTTCAAATATTGAATTAGGTGGGCATGTCGATTCT

w70_COWP (181) TTTTTAATCACTTTACATTTTCCATTTTCAAATATTGAATTAGGTGGGCATGTCGATTCT

C.meleagridis_COWP (181) TTTTTAATCACTTTGCATTTTCCATTTTCAAATATTGAATTAGGTGGGCATGTAGATTCT

241 300

ch2_COWP (241) AATTCAGCTGATTCTGGTGCCATACATTGTTGTCCTGACAAATTGAATCCAGGAGGACAG

ch3_COWP (241) AATTCAGCTGATTCTGGTGCCATACATTGTTGTCCTGACAAATTGAATCCAGGAGGACAG

ch4_COWP (241) AATTCAGCTGATTCTGGTGCCATACATTGTTGTCCTGACAAATTGAATCCAGGAGGACAG

TU502_COWP (241) AATTCAGCTGATTCTGGTGCCATACATTGTTGTCCTGACAAATTGAATCCAGGAGGACAG

rabbit1_COWP (241) AATTCAGCTGATTCTGGTGCCATACATTGTTGTCCTGACAAATTGAATCCAGGAGGACAG

rabbit2_COWP (241) AATTCAGCTGATTCTGGTGCCATACATTGTTGTCCTGACAAATTGAATCCAGGAGGACAG

rabbit3_COWP (241) AATTCAGCTGATTCTGGTGCCATACATTGTTGTCCTGACAAATTGAATCCAGGAGGACAG

rabbit4_COWP (241) AATTCAGCTGATTCTGGTGCCATACATTGTTGTCCTGACAAATTGAATCCAGGAGGACAG

cp2_COWP (241) AATTCAGCTGATTCTGGTGCCATACATTGTTGTCCTGACAAATTGAATCCAGGAGGACAG

cp3_COWP (241) AATTCAGCTGATTCTGGTGCCATACATTGTTGTCCTGACAAATTGAATCCAGGAGGACAG

cp4_COWP (241) AATTCAGCTGATTCTGGTGCCATACATTGTTGTCCTGACAAATTGAATCCAGGAGGACAG

Iowa_COWP (241) AATTCAGCTGATTCTGGTGCCATACATTGTTGTCCTGACAAATTGAATCCAGGAGGACAG

Moredun_COWP (241) AATTCAGCTGATTCTGGTGCCATACATTGTTGTCCTGACAAATTGAATCCAGGAGGACAG

w65_COWP (241) AATTCAGCTGATTCTGGTGCCATACATTGTTGTCCTGACAAATTGAATCCAGGAGGACAG

w66_COWP (241) AATTCAGCTGATTCTGGTGCCATACATTGTTGTCCTGACAAATTGAATCCAGGAGGACAG

w67_COWP (241) AATTCAGCTGATTCTGGTGCCATACATTGTTGTCCTGACAAATTGAATCCAGGAGGACAG

w70_COWP (241) AATTCAGCTGATTCTGGTGCCATACATTGTTGTCCTGACAAATTGAATCCAGGAGGACAG

C.meleagridis_COWP (241) AATTCAGCAGATTCTGGTGCCATACATTGTTGTCCTGACAAATTAAATCCAGGAGGACAG

301 360

ch2_COWP (301) ATTTTGTTTGCAGGGAGATATTGAACACATCTATTTCCTTCTTCAACAAAACCAGAAGGA

ch3_COWP (301) ATTTTGTTTGCAGGGAGATATTGAACACATCTATTTCCTTCTTCAACAAAACCAGAAGGA

ch4_COWP (301) ATTTTGTTTGCAGGGAGATATTGAACACATCTATTTCCTTCTTCAACAAAACCAGAAGGA

TU502_COWP (301) ATTTTGTTTGCAGGGAGATATTGAACACATCTATTTCCTTCTTCAACAAAACCAGAAGGA

rabbit1_COWP (301) ATTTTGTTTGCAGGGAGATATTGAACACATCTATTTCCTTCTTCAACAAAACCAGAAGGA

rabbit2_COWP (301) ATTTTGTTTGCAGGGAGATATTGAACACATCTATTTCCTTCTTCAACAAAACCAGAAGGA

rabbit3_COWP (301) ATTTTGTTTGCAGGGAGATATTGAACACATCTATTTCCTTCTTCAACAAAACCAGAAGGA

rabbit4_COWP (301) ATTTTGTTTGCAGGGAGATATTGAACACATCTATTTCCTTCTTCAACAAAACCAGAAGGA

cp2_COWP (301) ATTTTATTTGCAGGGAGATATTGAACACATCTATTTCCTTCTTCAACAAAACCAGAAGGA

cp3_COWP (301) ATTTTATTTGCAGGGAGATATTGAACACATCTATTTCCTTCTTCAACAAAACCAGAAGGA

cp4_COWP (301) ATTTTATTTGCAGGGAGATATTGAACACATCTATTTCCTTCTTCAACAAAACCAGAAGGA

Iowa_COWP (301) ATTTTATTTGCAGGGAGATATTGAACACATCTATTTCCTTCTTCAACAAAACCAGAAGGA

Moredun_COWP (301) ATTTTATTTGCAGGGAGATATTGAACACATCTATTTCCTTCTTCAACAAAACCAGAAGGA

w65_COWP (301) ATTTTATTTGCAGGGAGATATTGAACACATCTATTTCCTTCTTCAACAAAACCAGAAGGA

w66_COWP (301) ATTTTATTTGCAGGGAGATATTGAACACATCTATTTCCTTCTTCAACAAAACCAGAAGGA

w67_COWP (301) ATTTTATTTGCAGGGAGATATTGAACACATCTATTTCCTTCTTCAACAAAACCAGAAGGA

w70_COWP (301) ATTTTATTTGCAGGGAGATATTGAACACATCTATTTCCTTCTTCAACAAAACCAGAAGGA

C.meleagridis_COWP (301) ATTTTGTTTGCAGGGAGATATTGAACACATCTATTTCCTTCTTCAACAAAACCAGAAGGA

361 420

ch2_COWP (361) CAAACGGTATCAATTTGTTGAATTAATTTACATGTGCCATTTTCCAGTATAGTACCTGGA

ch3_COWP (361) CAAACGGTATCAATTTGTTGAATTAATTTACATGTGCCATTTTCCAGTATAGTACCTGGA

ch4_COWP (361) CAAACGGTATCAATTTGTTGAATTAATTTACATGTGCCATTTTCCAGTATAGTACCTGGA

TU502_COWP (361) CAAACGGTATCAATTTGTTGAATTAATTTACATGTGCCATTTTCCAGTATAGTACCTGGA

rabbit1_COWP (361) CAAACGGTATCAATTTGTTGAATTAATTTACATGTGCCATTTTCCAGTATAGTACCTGGA

rabbit2_COWP (361) CAAACGGTATCAATTTGTTGAATTAATTTACATGTGCCATTTTCCAGTATAGTACCTGGA

rabbit3_COWP (361) CAAACGGTATCAATTTGTTGAATTAATTTACATGTGCCATTTTCCAGTATAGTACCTGGA

rabbit4_COWP (361) CAAACGGTATCAATTTGTTGAATTAATTTACATGTGCCATTTTCCAGTATAGTACCTGGA

cp2_COWP (361) CAAACGGTATCAATTTGTTGAATTAATTTACATGTGCCATTCTCCAGTATAGTGCCTGGA

cp3_COWP (361) CAAACGGTATCAATTTGTTGAATTAATTTACATGTGCCATTCTCCAGTATAGTGCCTGGA

cp4_COWP (361) CAAACGGTATCAATTTGTTGAATTAATTTACATGTGCCATTCTCCAGTATAGTGCCTGGA

Iowa_COWP (361) CAAACGGTATCAATTTGTTGAATTAATTTACATGTGCCATTCTCCAGTATAGTGCCTGGA

Moredun_COWP (361) CAAACGGTATCAATTTGTTGAATTAATTTACATGTGCCATTCTCCAGTATAGTGCCTGGA

w65_COWP (361) CAAACGGTATCAATTTGTTGAATTAATTTACATGTGCCATTCTCCAGTATAGTGCCTGGA

w66_COWP (361) CAAACGGTATCAATTTGTTGAATTAATTTACATGTGCCATTCTCCAGTATAGTGCCTGGA

w67_COWP (361) CAAACGGTATCAATTTGTTGAATTAATTTACATGTGCCATTCTCCAGTATAGTGCCTGGA

w70_COWP (361) CAAACGGTATCAATTTGTTGAATTAATTTACATGTGCCATTCTCCAGTATAGTGCCTGGA

C.meleagridis_COWP (361) CAAACGGTATCAATTTGTTGAATTAATTTACATGTACCATTCTCTAGTATAGTGCCTGGA

421 480

ch2_COWP (421) GGACATTCTGGATTAGGAGGAGCTGTGTCTGATTGAACACATTGTTTTCCAGAAAAGACG

ch3_COWP (421) GGACATTCTGGATTAGGAGGAGCTGTGTCTGATTGAACACATTGTTTTCCAGAAAAGACG

ch4_COWP (421) GGACATTCTGGATTAGGAGGAGCTGTGTCTGATTGAACACATTGTTTTCCAGAAAAGACG

TU502_COWP (421) GGACATTCTGGATTAGGAGGAGCTGTGTCTGATTGAACACATTGTTTTCCAGAAAAGACG

rabbit1_COWP (421) GGACATTCTGGATTAGGAGGAGCTGTGTCTGATTGAACACATTGTTTTCCAGAAAAGACG

rabbit2_COWP (421) GGACATTCTGGATTAGGAGGAGCTGTGTCTGATTGAACACATTGTTTTCCAGAAAAGACG

rabbit3_COWP (421) GGACATTCTGGATTAGGAGGAGCTGTGTCTGATTGAACACATTGTTTTCCAGAAAAGACG

rabbit4_COWP (421) GGACATTCTGGATTAGGAGGAGCTGTGTCTGATTGAACACATTGTTTTCCAGAAAAGACG

cp2_COWP (421) GGACATTCTGGATTAGGAGGAGCTGTGTCTGATTGAACACATTGTTTTCCAGAAAAAACG

cp3_COWP (421) GGACATTCTGGATTAGGAGGAGCTGTGTCTGATTGAACACATTGTTTTCCAGAAAAAACG

cp4_COWP (421) GGACATTCTGGATTAGGAGGAGCTGTGTCTGATTGAACACATTGTTTTCCAGAAAAAACG

Iowa_COWP (421) GGACATTCTGGATTAGGAGGAGCTGTGTCTGATTGAACACATTGTTTTCCAGAAAAAACG

Moredun_COWP (421) GGACATTCTGGATTAGGAGGAGCTGTGTCTGATTGAACACATTGTTTTCCAGAAAAAACG

w65_COWP (421) GGACATTCTGGATTAGGAGGAGCTGTGTCTGATTGAACACATTGTTTTCCAGAAAAAACG

w66_COWP (421) GGACATTCTGGATTAGGAGGAGCTGTGTCTGATTGAACACATTGTTTTCCAGAAAAAACG

w67_COWP (421) GGACATTCTGGATTAGGAGGAGCTGTGTCTGATTGAACACATTGTTTTCCAGAAAAAACG

w70_COWP (421) GGACATTCTGGATTAGGAGGAGCTGTGTCTGATTGAACACATTGTTTTCCAGAAAAAACG

C.meleagridis_COWP (421) GGACATTCTGGATTAGGAGGAGCTGTGTCTGATTGAACACATTGCTTTCCAGAAAAAACG

481 529

ch2_COWP (481) AATCCTGGGGGACATGATTTTTCAGGCATAGTGAATGCAACACAATCTC

ch3_COWP (481) AATCCTGGGGGACATGATTTTTCAGGCATAGTGAATGCAACACAATCTC

ch4_COWP (481) AATCCTGGGGGACATGATTTTTCAGGCATAGTGAATGCAACACAATCTC

TU502_COWP (481) AATCCTGGGGGACATGATTTTTCAGGCATAGTGAATGCAACACAATCTC

rabbit1_COWP (481) AATCCTGGGGGACATGATTTTTCAGGCATAGTGAATGCAACACAATCTC

rabbit2_COWP (481) AATCCTGGGGGACATGATTTTTCAGGCATAGTGAATGCAACACAATCTC

rabbit3_COWP (481) AATCCTGGGGGACATGATTTTTCAGGCATAGTGAATGCAACACAATCTC

rabbit4_COWP (481) AATCCTGGGGGACATGATTTTTCAGGCATAGTGAATGCAACACAATCTC

cp2_COWP (481) AATCCTGGGGGACATGATTTTTCAGGCATAGTGAATGCAACACAATCTC

cp3_COWP (481) AATCCTGGGGGACATGATTTTTCAGGCATAGTGAATGCAACACAATCTC

cp4_COWP (481) AATCCTGGGGGACATGATTTTTCAGGCATAGTGAATGCAACACAATCTC

Iowa_COWP (481) AATCCTGGGGGACATGATTTTTCAGGCATAGTGAATGCAACACAATCTC

Moredun_COWP (481) AATCCTGGGGGACATGATTTTTCAGGCATAGTGAATGCAACACAATCTC

w65_COWP (481) AATCCTGGGGGACATGATTTTTCAGGCATAGTGAATGCAACACAATCTC

w66_COWP (481) AATCCTGGGGGACATGATTTTTCAGGCATAGTGAATGCAACACAATCTC

w67_COWP (481) AATCCTGGGGGACATGATTTTTCAGGCATAGTGAATGCAACACAATCTC

w70_COWP (481) AATCCTGGGGGACATGATTTTTCAGGCATAGTGAATGCAACACAATCTC

C.meleagridis_COWP (481) AATCCTGGAGGACATGATTTTTCAGGCATAGTAAATGCAACACAATCTC
